# Regulation of diel locomotor activity and retinal responses of *Anopheles stephensi* by ingested histamine and serotonin is temperature- and infection-dependent

**DOI:** 10.1371/journal.ppat.1013139

**Published:** 2025-04-28

**Authors:** Kevin O. Ochwedo, Xiaodi Wang, Nora Céspedes, Ronald E. Bentil, Ryan Wild, Emily Hernandez, Amy Hernandez, Hannah L. Kaylor, Yared Debebe, Jyotishka Datta, Michael A. Robert, Jeffrey A. Riffell, Edwin E. Lewis, Shirley Luckhart

**Affiliations:** 1 Department of Entomology, Plant Pathology, and Nematology, University of Idaho, Moscow, Idaho, United States of America; 2 Department of Biology, University of Washington, Seattle, Washington, United States of America; 3 Department of Statistics and Center of Biostatics and Health Data Science, Virginia Tech, Blacksburg, Virginia, United States of America; 4 Department of Mathematics, Center for the Mathematics of Biosystems; and Center for Emerging, Zoonotic, and Arthropod-Borne Pathogens (CeZAP), Virginia Tech, Blacksburg, Virginia, United States of America; 5 Department of Biological Sciences, University of Idaho, Moscow, Idaho, United States of America; National Institutes of Health, UNITED STATES OF AMERICA

## Abstract

Disrupting behaviors linked to movement of primary mosquito vectors, such as diel locomotor activity and visual sensitivity, is a novel and plausible malaria control intervention. Diel locomotor activity is an output of arthropod circadian activity and is influenced by factors such as light, temperature, and infection status. The biogenic amines histamine and serotonin (5-HT) are ingested with blood and differ between healthy hosts and those with severe malaria. They regulate malaria parasite infection in *Anopheles stephensi*, but the degree to which aging, temperature, and infection interact with ingested biogenic amines to influence mosquito behavior was unknown prior to these studies. We provisioned *A. stephensi* with histamine and 5-HT at healthy- and malaria-associated levels to examine diel locomotor activity of uninfected *A. stephensi* across lifespan, at temperatures that *A. stephensi* could encounter within its range, and on *Plasmodium yoelii*-infected mosquitoes during sporogony. We further evaluated treatment effects on retinal sensitivity of uninfected mosquitoes during light and dark periods typically associated with low and high activity for this crepuscular species. Treatment with malaria-associated levels of histamine and 5-HT significantly increased the locomotor activity of *A. stephensi* across lifespan and enhanced retinal sensitivity to a broad spectrum of wavelengths at the onset of light. This treatment in combination with higher temperatures also increased activity levels and broadened the peak hours of activity of *A. stephensi*. Notably, these effects were infection dependent. Together, our data suggest that histaminergic and serotonergic signaling within the gut-brain axis of *A. stephensi* could be targeted to alter mosquito activity and visual sensitivity as the basis for novel transmission-blocking strategies for malaria control.

## Summary

This study explores histaminergic and serotonergic signaling-dependent diel locomotor activity and visual sensitivity in the highly invasive malaria vector *Anopheles stephensi*. Histamine and 5-HT blood levels are substantially altered in malaria, yielding multiple possible outcomes of ingested biogenic amines and parasite infection in *A. stephensi*. Ingested malaria-associated levels of histamine and 5-HT altered *A. stephensi* locomotor activity across lifespan in patterns that were temperature- and infection-dependent. Malaria-associated treatment also increased retinal sensitivity at the onset of light, an inverse pattern to that of control treatment for this crepuscular mosquito. These findings suggest that disruption of histaminergic and serotonergic signaling using attractive targeted bait station delivery of specific antagonists could substantially alter uninfected and infected vector behavior as a novel malaria control strategy.

## Introduction

Malaria remains a global health burden, with sub-Saharan Africa being the most impacted [1]. Efforts to achieve the global objective of eliminating malaria in 30 countries by 2030 [2] are under threat due to emerging challenges such as climate change, anthropogenic changes to the landscape, and the recent arrival and distribution of invasive *Anopheles stephensi* mosquitoes across sub-Saharan Africa [3,4]. These challenges necessitate innovative strategies that can supplement current ones. Understanding the biology and behaviors of malaria vectors, particularly behavior associated with movement, is necessary for reducing the spread of the disease. Circadian rhythms are essential biological processes that regulate behavior, development, and survival of malaria vectors [5]. Diel locomotor activity, which is an output of circadian activity, is modulated externally by factors such as light, which entrains and synchronizes the circadian clock, and temperature, as well as internally by infection status [6,7]. In *Anopheles* mosquitoes, circadian rhythms of activity influence mating swarm formation, host-seeking, oviposition site selection, migration, and blood feeding, all of which directly impact malaria parasite transmission [8,9].

During feeding, *Anopheles* mosquitoes ingest human blood containing biogenic amines, including histamine and serotonin (5-HT), at concentrations that are dependent on host infection and disease status. Individuals with severe malaria can present with high blood histamine levels [10,11] and low levels of 5-HT [12]. Specifically, histamine levels can increase to 10-fold above healthy levels or as high as 10 nM, while 5-HT levels can decline by 10-fold to as low 0.15 µM compared to levels in healthy adults and children [13]. We previously demonstrated that histamine and 5-HT at concentrations associated with severe malaria alter *A. stephensi* blood feeding, infection, flight activity, and response to visual stimuli [14–16].

Histamine and 5-HT exert their effects in animals through histaminergic and serotonergic signaling pathways. Histamine plays a key role in photoreception, mechanosensitivity, temperature sensing, and circadian rhythm in insects. In the visual system, light stimulates photoreceptor neurons to depolarize and release histamine, which then binds to histamine 1 (H1) receptors (G-protein coupled receptors) in postsynaptic neurons, facilitating the transmission of visual information to the arthropod brain [17,18]. The regulation of histamine release from photoreceptor neurons enables arthropods to detect variations in light intensity [17]. Histaminergic neurons also connect the ventral nerve cord to the insect brain. When stimulated by external sensorimotor inputs, these neurons convey information to the brain, which then refines the motor response, resulting in specific flight behavior and wing motion [19]. In addition, histidine decarboxylase (hdc), an enzyme that converts histidine to histamine, and two additional histamine receptors, *ora transientless* (*ort*) and histamine-gated chloride channel subunit 1 (*hisCl1*), facilitate the acclimatization of arthropods to extreme temperatures [20,21]. This is also observed in mammalian cells, where elevated temperatures enhance hdc activity, resulting in increased histamine concentrations that facilitate positive adaptation [22,23]. *Ort* and *hisCl1* also function in circadian entrainment and have a role in resting-activity cycling in the fruit fly *Drosophila melanogaster* [24]. In the Madera cockroach *Rhyparobia maderae*, histamine elicited a phase shift in rhythms of locomotory activity rhythm that mirrored light-induced delays when injected into the compound eye [25]. Histamine also contributes to energy production and stimulates the glycolytic pathway in mammalian cells in a dose-dependent manner, leading to energy production via the activation of H1 and H2 receptors [26,27].

5-HT, unlike histamine that directly affects photoreceptors, indirectly regulates photoreception in arthropods [28]. 5-HT modulates voltage-gated potassium channels, including *Shaker*, by reducing their activity or rapidly closing the channels at elevated levels [28]. Rapid closure of the *Shaker* channel can induce a more depolarized state or prolong the action potential impacting neuronal function. 5-HT receptors are also involved in mechanosensory processes and are induced by external cues that initiate motor activity and locomotion [29–33]. Low levels of 5-HT in serotonergic neurons in the ventral nerve cord signal hunger and activate the motor circuit for locomotion, whereas elevated levels suppress these processes [32,33]. The presence of 5-HT in insect eyes affects circadian rhythm entrainment by inducing phase shifts, primarily causing phase delays in biological clock timing and locomotor activity [34,35]. The phase shift effect occurs when 5-HT elevates cyclic adenosine monophosphate (cAMP) levels, which then mediate the phase delay at certain intervals in the circadian rhythm [35]. Under thermal stress such as cold exposure, 5-HT transporter and receptor 7 (5-HT7) genes are usually upregulated, indicating their role in facilitating organismal acclimatization [36]. 5-HT also induces passive or active physiological alterations in muscles in response to temperature-dependent neural input, hence expanding the temperature range for eliciting motor action or movements [37]. In adverse conditions such as winter, high levels of 5-HT are critical for diapause in arthropods [38]. As opposed to histamine, 5-HT levels are influenced by aging, and whole body titers in *D. melanogaster* decline with age [39]. The impact of the combined effects of histamine and 5-HT on diel locomotor activity and retinal response of *A. stephensi* is currently undetermined, as is the interaction of these effects on locomotor activity with aging, temperature and infection.

To understand the effects of treatment, temperature, age, and infection in our studies, it is critical to revisit possible outcomes of mosquito feeding on human hosts. Our experimental design is based on published observations in falciparum malaria as are the following statements. Mosquitoes can feed on human hosts that are uninfected (S1 Fig). In this context, mosquitoes ingest healthy level of blood histamine and 5-HT and remain uninfected. Other mosquitoes can feed on human hosts with asymptomatic parasitemia and transmissible gametocytes [40]. Feeding on a host with transmissible gametocytes, however, does not guarantee mosquito infection, with reports that only 34–76% of mosquitoes become infected after feeding on infectious human host blood [41]. In this case, some mosquitoes would be infected while others would not. Regardless of infection, however, mosquitoes that feed on human hosts with asymptomatic parasitemia would ingest blood histamine and 5-HT at levels more consistent with a healthy state (S1 Fig). This is evidenced by levels of human basophil reactivity, a marker of histamine release and hence histamine-dependent downregulation of serotonin, in infected individuals without signs of severe falciparum disease that were not different from healthy controls[42–44]. Yet other mosquitoes can feed on human hosts with symptomatic, perhaps severe, malaria and transmissible gametocytes. Again, feeding on these hosts does not guarantee mosquito infection, so some mosquitoes would be infected, and others would not, but all would ingest blood histamine and 5-HT at levels consistent with malarial disease (S1 Fig). Accordingly, we have examined the effects of ingesting healthy- and malaria-associated levels of histamine and 5-HT on both infected and uninfected *A. stephensi*. Our parameters included activity throughout lifespan, at temperatures that *A. stephensi* could encounter within its range and in the presence of parasite infection, to identify significant interactions that could enhance vector survival, *Plasmodium* development and transmission.

## Results

### Weekly provisioning of healthy- or malaria-associated levels of histamine + 5-HT had no effect on uninfected *A.stephensi* lifespan

**Fig 1:**
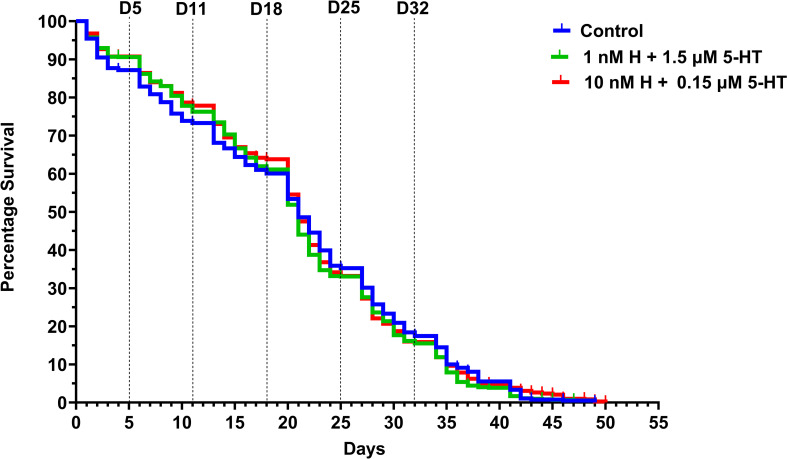
Median survival of uninfected *A. stephensi* provisioned with histamine + 5-HT. Mosquitoes were provisioned weekly with healthy-associated 1 nM histamine (H) + 1.5 µM 5-HT (green), malaria-associated 10 nM H + 0.15 µM 5-HT (red), or water-soaked cotton balls (blue) from day 0 to day 3, followed by bloodmeals with no supplement. N = 4 biological replicates (control n = 1018, healthy n = 988, malaria n = 985). Kruskal-Wallis test with Dunn’s multiple comparisons test revealed no significant differences.

Median survival was compared among mosquitoes provisioned with healthy-associated levels of biogenic amines [1 nM histamine (H) + 1.5 µM 5-HT], malaria-associated levels [10 nM H + 0.15 µM 5-HT,] or water-soaked cotton balls (control). Survival data were analyzed using the Kaplan-Meier test followed by log-rank or Mantel-Cox test. Overall, treatment effects on median survival were not significant across replicates (Fig 1). This was likely due to high levels of variation among replicates within treatments (S2A–D Fig and S1 Table).

### Weekly provisioning of malaria-associated levels of histamine + 5-HT increased uninfected *A. stephensi* locomotor activity over lifespan

We next asked whether biogenic treatment altered diel locomotor activity and whether effects might change with increasing *A. stephensi* age. To address this, control and treated mosquitoes (n = 300 per group) were provisioned with biogenic amine treatments (primed) or water only (control) for three days each week. After priming, eight females from each group for each replicate were removed for locomotor activity monitoring. The remaining females were given bloodmeals and kept for activity monitoring weekly for the remaining four weeks.

**Fig 2:**
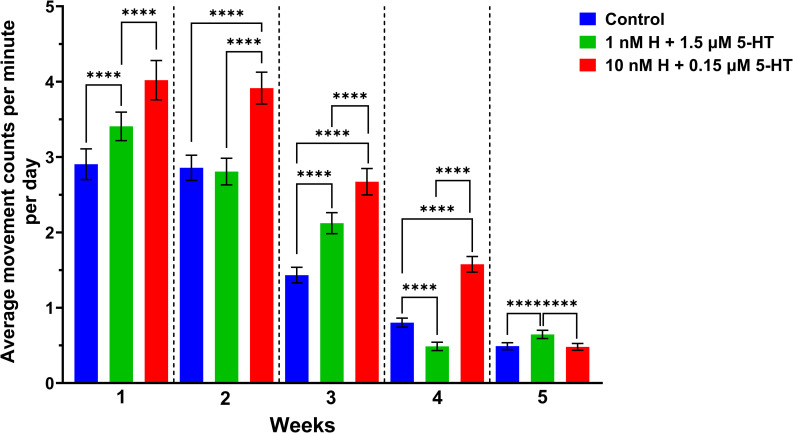
Diel locomotor activity over lifespan of uninfected *Anopheles stephensi* provisioned with histamine + 5-HT. The bars represent mean of movement counts or beam breaks ± standard errors of the mean per minute of four biological replicates at week 1: control (n = 27, 2.90 ± 0.20), healthy (n = 31, 3.41 ± 0.19), malaria (n = 27, 4.02 ± 0.26), at week 2: control (n = 31, 2.86 ± 0.17), healthy (n = 28, 2.81 ± 0.18), malaria (n = 29, 3.92 ± 0.21), at week 3: control (n = 31, 1.43 ± 0.10), healthy (n = 32, 2.12 ± 0.14), malaria (n = 30, 2.67 ± 0.17), at week 4: control (n = 29, 0.80 ± 0.06), healthy (n = 29; 0.49 ± 0.06), malaria (n = 30, 1.58 ± 0.10), and at week 5: control (n = 24, 0.49 ± 0.05), healthy (n = 25, 0.65 ± 0.06), malaria (n = 27, 0.48 ± 0.05). Mosquitoes provisioned with malaria-associated biogenic amines (10 nM H + 0.15 µM 5-HT; red bars) displayed significantly greater overall activity in weeks 1-4 when compared to those provisioned with healthy-associated biogenic amines (1 nM H + 1.5 µM 5-HT; green bars). Pairwise comparisons within weeks were performed using Tukey HSD. P values ≤ 0.05 were considered significant. * P ≤ 0.05, ** P ≤ 0.01, ***P ≤ 0.001, **** P ≤ 0.00001.

As expected, locomotor activity levels of uninfected *A. stephensi* declined across all treatment groups as mosquitoes aged (Fig 2 and S2 Table). Mosquitoes primed with the malaria-associated biogenic amine treatment, however, had significantly higher (P < 0.0001) overall activity compared with mosquitoes primed with the healthy-associated biogenic amine treatment or controls across weeks 1–4 (Fig 2 and S2 Table). A generalized linear mixed model (GLMM) with Poisson distributed response was used to examine the effect of age and treatments on locomotor activity. The two main effects, age (χ2 = 11788.39, df = 4, P < 0.0001) and treatment (χ2 = 769.54, df = 4, P < 0.0001), had significant impacts on locomotor activity of uninfected *A. stephensi*. The interaction between the main effects was also significant (χ2 = 743.571, df = 8, P < 0.0001).

To further examine the effects of biogenic amine treatments, we quantified the active periods during the 12-hour light and dark cycles by converting activity counts recorded per minute to a binary format (1 = activity, 0 = no activity) and averaged these for light and dark cycles (S3 Fig). In general, all groups showed higher levels of activity during darkness across lifespan with an exception for control mosquitoes at week 5 (S3 Fig). From weeks 2–5, the malaria-associated treatment group exhibited more light cycle activity compared to mosquitoes provided with the healthy-associated biogenic amine treatment, with these differences statistically significant in weeks 2, 4, and 5 (S3 Fig and S3 Table). During dark cycles, however, the two treatment groups varied, for which the malaria-associated treatment group exhibited significantly higher activity in weeks 2 and 4 (S3 Fig and S3 Table).

**Fig 3:**
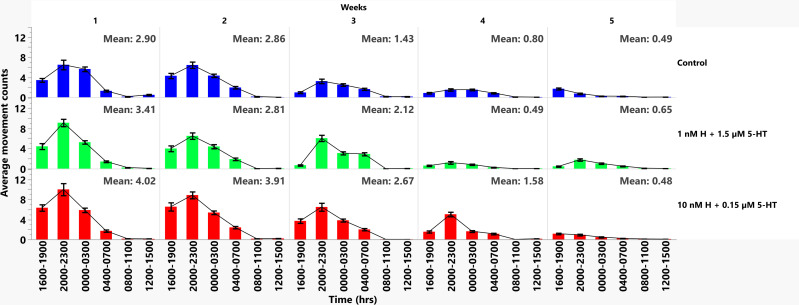
Mean movement counts over the lifespan of uninfected *A. stephensi* provisioned with histamine + 5-HT. The blue (control), green (1nM H + 1.5 µM 5-HT) and red (10nM H + 0.15 µM 5-HT) bars represent mean movement counts and standard errors of the mean at three-hour intervals within 24 hours for four biological replicates (week1; control n = 27, healthy n = 31, malaria n = 27, week 2; control n = 31, healthy n = 28, malaria n = 29, week 3; control n = 31, healthy n = 32, malaria n = 30, week 4; control n = 29, healthy n = 29, malaria n = 30, week 5; control n = 24, healthy n = 25, malaria n = 27). Peak activity in uninfected mosquitoes provisioned with malaria-associated levels of biogenic amines (10 nM H + 0.15 µM HT) occurred in late evening (2000-2300 h) followed by early evening (1600-1900 h), with minor peaks at midnight (0000-0300 h), and dawn (0400-0700 h) over lifespan. Mosquitoes provisioned with healthy-associated levels (1 nM H + 1.5 µM 5-HT) and water (control) had similar patterns of activity with peak activity in both treatment groups being observed in late evening (2000-2300 h), followed by midnight (0000-0300 h), then early evening (1600-1900 h), and dawn (0400-0700 h) over lifespan.

To identify the periods with the highest levels of activity by week, we analyzed the data from Fig 2 in three-hour increments over a 24-hour time period (Fig 3). Within-week comparisons of malaria-associated and healthy-associated groups revealed notable activity differences. In weeks 1 and 2, activity levels in the early evening (1600–1900 h), late evening (2000–2300 h) and midnight (0000–0300 h) in the malaria-associated group were higher than those in corresponding periods in the healthy-associated group (Fig 3 and S4 Table). By week 3, increased activity in the malaria-associated group relative to the healthy group was restricted to early evening (1600–1900 h), with reduced activity in the malaria- versus healthy-associated group at dawn (0400–0700 h; Fig 3 and S4 Table). By week 4, the malaria-associated group had increased activity relative to the healthy-associated group in late evening (2000–2300 h) and dawn (0400–0700 h; Fig 3 and S4 Table). The greatest relative difference in mean movement counts across treatment groups was also observed in week 4 (malaria associated = 1.58, healthy associated = 0.49; Fig 3). By week 5, there were no significant differences in activity across time periods between malaria- and healthy-associated groups and mean activity in the healthy-associated group (0.65) was higher than mean activity of the malaria-associated group (0.48; Fig 3 and S4 Table). Based on these observations, we inferred that increased light cycle activity of the malaria-associated group relative to the healthy-associated group from weeks 1–4 (S3 Fig) was occurring primarily in the early evening (1600–1900 h) and late evening (2000–2300 h).

We fitted a generalized linear model with a Poisson link function to the movement data where movement counts were modeled as a function of treatment levels, week, and time in hours, discretized into 3-hour categories (S4–S7 Figs and S5 Table). In comparison to the healthy-associated treatment, the malaria-associated treatment significantly increased the incidence rate ratios (IRRs) of movement counts across lifespan by 1.34 (95% CI: 1.31, 1.37), while controlling for the other variables (S5 Fig). Due to the zero inflated nature of the data, a zero-inflated Poisson regression with a two-parts model, a logit for the probability of zeroes and a Poisson GLM for the counts was used to account for the excess zeroes. The IRR estimates for the probability of excess zeroes (which is also considered as periods of inactivity or resting) exhibited an inverse trend compared to the movement counts; For instance, the odds of excess zeroes for the control group versus healthy group was 1.05, and the malaria group versus healthy group was 0.75. This indicated more zeroes in the control group compared to the healthy group, and higher incidence of zeroes in the healthy group compared to the malaria group across lifespan (S7 Fig).

### Weekly provisioning of malaria-associated levels of histamine + 5-HT enhanced retinal responses after the onset of light in uninfected *A. stephensi*

**Fig 4:**
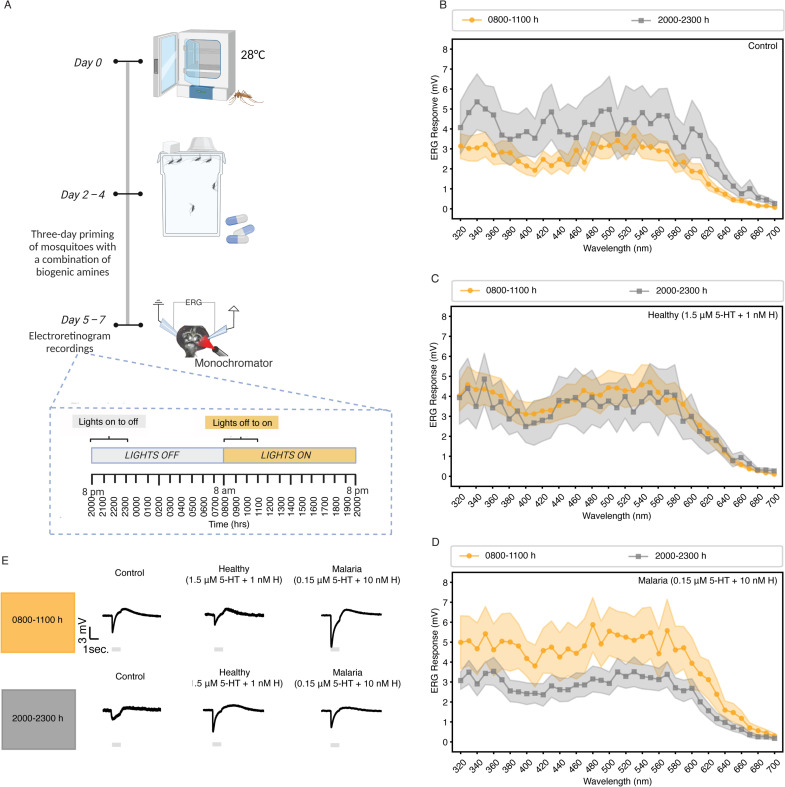
Electroretinogram recordings (ERG) under different light conditions of uninfected female *A. stephensi* provisioned with histamine + 5-HT. **(A)** Schematic of experimental protocol. Female *A. stephensi* were maintained in an incubator set to 28 °C and provisioned with biogenic amines for three days (created in BioRender, https://BioRender.com/w95g695). ERG recordings were performed using normalized light stimuli ranging from 320 to 700 nm in 10 nm increments within 3 h of light transition (on or off) for each group, where lights off to on = 0800-1100 h and lights on to off = 2000-2300 h. (B–D) ERG responses (Y-axis) to a range of light stimuli (X-axis) for mosquitoes provisioned with water (B, control), healthy-associated biogenic amines (C), and malaria-associated biogenic amines (D). Mean responses across individuals (n = 7–11 per treatment) are shown as dots or squares, and shaded areas indicate the standard error of the mean. Grey represents light transitions from on to off (2000-2300 h), and yellow indicates off to on (0800-1100 h). (E) Representative ERG trace for a single mosquito at 550 nm across treatment and light transition conditions.

Since malaria-associated biogenic amine treatment enhanced locomotor activity of uninfected *A. stephensi* at peak activity after lights off (2000–2300 h), we asked if mosquito retinal responses in this late evening period (2000–2300 h) relative to the first light period (0800–1100 h) were altered by biogenic amine treatments. We primed 5–7 day old uninfected female *A. stephensi* for three days with healthy- or malaria-associated biogenic amine treatments, while controls were primed with water (n = 7–11 per group). Mosquitoes were maintained at 28 °C and their retinal responses were measured by electroretinogram (ERG). Measurements reflected the total receptor neuron responses in the eye to different light wavelengths (320–700 nm). Overall, our recordings showed that uninfected female *A. stephensi* had peak or near peak retinal responses at 330–360 nm and 480–570 nm, regardless of biogenic amine treatment (Fig 4). Evaluating effects of biogenic amine treatments over these time periods with a GLMM model revealed that neither the malaria treatment (*β*=1.545, SE = 1.024, P = 1.508) nor the healthy treatment (*β*=0.860, SE = 0.977, P = 0.881) alone had a significant effect on retinal responses compared to the control group (S6 Table). Compared to the period after the onset of light (0800-1100h), the period after onset of dark (2000–2300 h) did not show significant effects (*β*=1.754, SE = 1.096, P = 0.109). However, our results showed that malaria-associated biogenic amine treatment led to higher ERG amplitudes after the onset of light (0800–1100 h) than the healthy-associated treatment or control (*β*=-3.158, SE = 1.522, P = 0.038; Fig 4). From our observations, the highest activity of the malaria-associated treatment group relative to healthy group occurred after the onset of darkness (2000–2300 h; Fig 3), while higher retinal responses in the malaria-associated treatment group were measured after the onset of light (0800–1100 h; Fig 4).

### Weekly provisioning of malaria-associated levels of histamine + 5-HT was associated with higher light and dark period activity of *A. stephensi* at higher temperature

Given that *A. stephensi* can experience a range of environmental temperatures in the field, we asked whether the effects of biogenic amine treatment on locomotor activity were altered by temperature. To address this, we prepared control and treatment groups that were primed for three days at four constant temperatures. We selected temperatures within a typical natural range for transmission of malaria parasites by *A. stephensi* [45]. After three days of priming, diel locomotor activity of eight mosquitoes from each group for each replicate was monitored.

**Fig 5:**
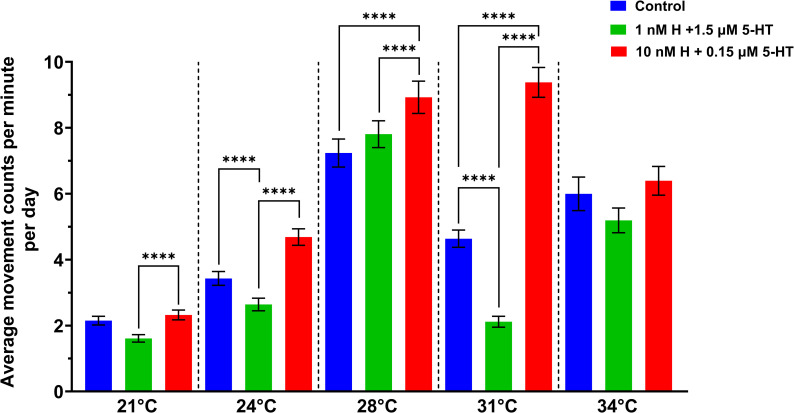
Diel locomotor activity at different temperatures for uninfected *A. stephensi* provisioned with histamine + 5-HT. The bars represent mean of movement counts or beam brakes ± standard errors of the mean per minute of 3 biological replicates at 21 °C: control (n = 23, 2.15 ± 0.13), healthy (n = 22, 1.61 ± 0.11), malaria (n = 24, 2.32 ± 0.15), at 24 °C: control (n = 24, 3.43 ± 0.21), healthy (n = 24, 2.64 ± 0.19), malaria (n = 24, 4.69 ± 0.25), at 28 °C: control (n = 23, 7.24 ± 0.43), healthy (n = 21, 7.81 ± 0.40), malaria (n = 22, 8.93 ± 0.49), at 31 °C: control (n = 24, 4.64 ± 0.26), healthy (n = 22, 2.12 ± 0.17), malaria (n = 24, 9.38 ± 0.45), and at 34 °C: control (n = 21, 6.00 ± 0.51), healthy (n = 19, 5.19 ± 0.38), malaria (n = 22, 6.39 ± 0.44). Mosquitoes provisioned with malaria-associated biogenic amines (10 nM H + 0.15 µM HT; red bars) displayed increasing activity to 31 °C whereas those provisioned with healthy-associated biogenic amines (1 nM H + 1.5 µM 5-HT; green bars) or water (controls, blue bars) plateaued at 28 °C. Mosquitoes treated with malaria-associated biogenic amines exhibited significantly increased locomotor activity relative to mosquitoes treated with healthy-associated concentrations at all temperatures except 34 °C, where no significant differences among treatments were observed. Pairwise comparisons of the means were performed with Tukey HSD. P values ≤ 0.05 were considered significant. * P ≤ 0.05, ** P ≤ 0.01, ***P ≤ 0.001, **** P ≤ 0.00001.

Mosquitoes treated with malaria-associated levels of biogenic amines were the most active relative to control or both control and healthy groups (P < 0.0001) at all temperatures except for 34 °C (Fig 5). To determine whether temperature interacted with treatment to influence locomotor activity, we used a GLMM (Poisson regression) with temperature and treatments as the main effects. Both temperature (χ^2^ = 16969.87, df = 4, P < 0.0001) and treatment (χ^2^ = 4030.73, df = 2, P < 0.0001) had significant effects on the activity of uninfected *A. stephensi*. Temperature interacted with biogenic amine treatments significantly (χ^2^ = 3977.01, df = 8, P < 0.0001). Across treatment groups, the lower temperatures of 21 °C and 24 °C were associated with low activity whereas the effect of high temperature (31 °C) was dependent on treatment. Comparing 28 °C and 31 °C, the activity of the malaria-associated treatment group, unlike the other two groups, was not impacted negatively at the higher temperature. Instead, the malaria-associated treatment group exhibited steadily increasing levels of activity from 21 °C to 31 °C, with reduced activity at 34 °C (Fig 5). In contrast, healthy and control groups displayed the highest levels of activity at 28 °C (our colony rearing temperature), with reduced activity levels at 31 °C and 34 °C (Fig 5).

**Fig 6:**
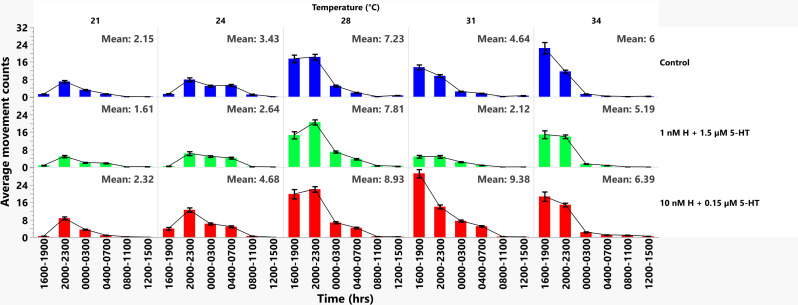
Mean movement counts at different temperatures for uninfected *A. stephensi* provisioned with histamine + 5-HT. The blue (control), green (1nM H + 1.5 µM 5-HT) and red (10nM H + 0.15 µM 5-HT) bars represents mean movement counts and standard errors of the mean at three-hour intervals within 24 hours for three biological replicates (21 °C; control n = 23, healthy n = 22, malaria n = 24, 24 °C; control n = 24, healthy n = 24, malaria n = 24, 28 °C; control n = 23, healthy n = 21, malaria n = 22, 31 °C; control n = 4, healthy n = 22, malaria n = 24, 34 °C; control n = 21, healthy n = 19, malaria n = 22). Treatments at lower temperatures 21 °C and 24 °C were associated with later peaks (2000–2300 h), whereas standard rearing temperature (28 °C) and higher temperatures of 31 and 34 °C were associated with earlier peaks (1600-1900 h). Mosquitoes treated with malaria-associated biogenic amines exhibited generally higher activity versus mosquitoes treated with healthy-associated biogenic amines.

To identify the periods with highest activity by treatment at each temperature, we analyzed the data from Fig 5 in three-hour increments over a 24-hour time period (Fig 6). Among mosquitoes provisioned with malaria-associated biogenic amine treatment, the highest active periods at the lowest temperatures (21 and 24 °C) were late evening (2000–2300 h), followed by 0000–0300 h, 0400–0700 h, and 1600–1900 h, respectively, with significant differences in activity between these time periods (S7 Table). At 28 °C, activity was increased in early evening (1600–1900 h), followed by late evening (2000–2300 h). Contrary to lower temperatures, at 31 °C and 34 °C the highest active period was in early evening (1600–1900 h), then late evening (2000–2300 h), midnight (0000–0300 h) and dawn (0400–0700 h), respectively (S7 Table). The differences in activity patterns between the time points at 31 °C and 34 °C were significant for the malaria-associated treatment group (S7 Table). When we compared activity across treatment groups, the malaria-associated treatment group had significantly higher activity compared to the healthy group at early evening (1600–1900 h) at 24 °C and 31 °C, late evening (2000–2300 h) at 21 °C and 31 °C, midnight (0000–0300 h) at 31 °C and 34 °C and dawn (0400–0700 h) at 31 °C (S8 Table). Although activity levels were very low from 0800-1100 h and 1200–1500 h, the malaria-associated treatment group had significantly higher activity than the healthy-associated group at 24 °C (0800–1100 h), 31 °C (0800–1100 h) and 34 °C (0800–1100 h, 1200–1500 h; S8 Table). The healthy-associated treatment group exhibited significantly higher activity than the malaria group only at dawn (0400–0700 h) at 21 °C (S8 Table). Based on these observations, the broadest period of increased activity in the malaria-associated treatment group compared with the healthy group (1600–1100 h) occurred at 31 °C, the temperature at which mean movement counts across time periods (mean = 9.38) were also most different from the healthy-associated treatment group (mean = 2.12, Fig 6).

To further examine the effects of biogenic amines and temperature on active periods during the 12-hour light and dark periods, activity counts per minute were converted to a binary format (1 = activity, 0 = no activity) and totaled for light and dark cycles (S8 Fig). Dark period activity levels for all treatments were higher than light period activity levels at all temperatures (S8 Fig and S9 Table). Mosquitoes provisioned with the malaria-associated biogenic amine treatment had increasing activity levels in both light and dark periods up to 31 °C, with notably lower dark activity at 34 °C compared to peak dark activity at 31 °C. In contrast, overall dark and light period activity levels of controls and mosquitoes treated with healthy-associated levels of biogenic amines were higher at 28 °C relative to lower and higher temperatures (S8 Fig and S9 Table).

Similar to lifespan versus activity data, we fitted a generalized linear model with a Poisson link function to the movement data where movement counts were modeled as a function of treatment levels, temperature, and time in hours, discretized into 3-hour categories (S9–S12 Figs and S10 Table). In comparison to healthy treatment, the provisioned malaria-associated treatment significantly increased the IRRs of movement counts across temperature range by 1.64 (95% CI: 1.61, 1.66), while controlling for the other variables (S10 Fig). The probability of excess zeroes (resting or inactivity) for the control group compared to the healthy group was 0.76, while the malaria group compared to the healthy group was 0.46 across the temperature range. This showed more zeroes or resting periods in the healthy group compared to both the control and malaria group (S12 Fig).

### Malaria-associated histamine + 5-HT treatment increased locomotor activity of *A. stephensi* regardless of infection status, but these effects varied with time post-bloodmeal and time

In our previous work, we observed that *A. stephensi* primed with malaria-associated histamine and 5-HT in combination exhibited higher mean *Plasmodium yoelii* oocyst infection intensity (oocysts per midgut) and salivary gland infection prevalence (presence of detectable sporozoites) relative to mosquitoes treated with healthy-associated biogenic amines [15]. Based on the effects of biogenic amines described above on uninfected *A. stephensi* behavior, we asked whether parasite infection might modify the effects of biogenic amine treatment on *A. stephensi* locomotor activity. To address this question, we prepared four biological replicates of two sets of the three treatment groups described for experiments above: control mosquitoes and mosquitoes treated with healthy- and malaria-associated biogenic amines (n = 120 female mosquitoes per group). Each group was primed for three days with the appropriate treatment, after which one set was allowed to feed on uninfected mice, and the other set was allowed to feed on *P. yoelii-*infected mice. Diel locomotor activity of 8–16 mosquitoes per group was monitored on days 4, 10, and 14 post bloodmeal, which for infected mosquitoes corresponded to early oocyst, mature oocyst and sporozoite developmental stages of *P. yoelii* parasites, respectively.

**Fig 7:**
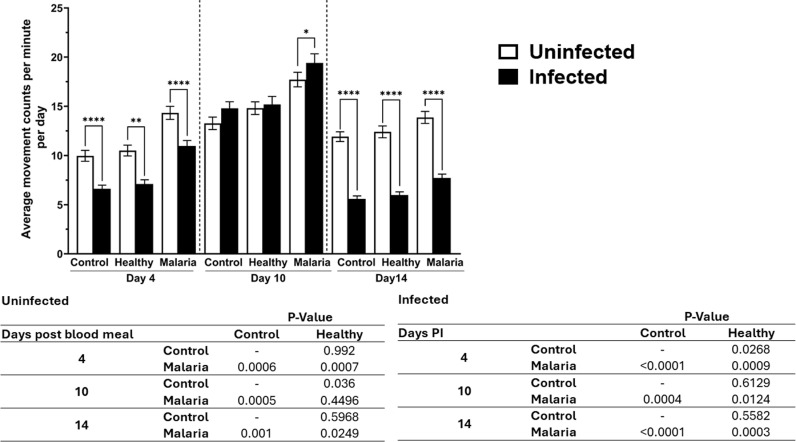
Diel locomotor activity of uninfected and *P. yoelii*-infected *A. stephensi* provisioned with histamine + 5-HT. The bars represent mean of movement counts or beam breaks ± standard errors of the mean per minute of five biological replicates of uninfected mosquitoes at day 4: control (n = 48, 9.97 ± 0.55), healthy (n = 47, 10.51 ± 0.55), malaria (n = 57, 14.33 ± 0.67), at day 10: control (n = 48, 13.27 ± 0.64), healthy (n = 53, 14.81 ± ,0.65), malaria (n = 54, 17.72 ± 0.74), at day 14: control (n = 51, 11.91 ± 0.50), healthy (n = 51, 12.4 ± 0.60), malaria (n = 50, 13.87 ± 0.61), and for infected mosquitoes at day 4: control (n = 43, 6.63 ± 0.36), healthy (n = 44, 7.10 ± 0.42), malaria (n = 46, 10.98 ± 0.55), at day 10: control (n = 63, 14.8 ± 0.66), healthy (n = 64, 15.19 ± 0.82), malaria (n = 70, 19.42 ± 0.93), and at day 14: control (n = 34, 5.58 ± 0.31), healthy (n = 35, 5.99 ± 0.32), malaria (n = 26, 7.72 ± 0.39). Mosquitoes provisioned with malaria-associated (10 nM H + 0.15 µM HT) or healthy-associated (1 nM H + 1.5 µM 5-HT) biogenic amines or water (control) for three days were subsequently allowed to feed on uninfected or *P. yoelii*-infected mice, then assayed at 4, 10, or 14 days post-feeding for diel locomotor activity. At 4- and 14-days post-feeding, infected mosquitoes in all groups were significantly less active than uninfected mosquitoes. This pattern was not observed at day 10 post-feeding, as mosquitoes harboring *P. yoelii*. Oocysts trended towards higher locomotor activity, with significantly higher activity than uninfected mosquitoes in the malaria group on this day. Malaria-associated treatment groups were associated with higher locomotor activity in both infected and uninfected groups at all timepoints compared to healthy-associated treatment groups (1 nM H + 1.5 µM 5-HT) and control. Pairwise comparisons (table) were done using Tukey HSD. P values ≤ 0.05 were considered significant. * P ≤ 0.05, ** P ≤ 0.01, ***P ≤ 0.001, **** P ≤ 0.00001.

Across all time points post bloodmeal, both uninfected and infected mosquitoes treated with malaria-associated biogenic amines tended to have higher activity levels compared to uninfected and infected mosquitoes treated with healthy-associated biogenic amines and controls (Fig 7). On days 4 and 14, diel locomotor activity of infected mosquitoes was significantly lower than uninfected mosquitoes in all groups, a pattern not observed at day 10 (Fig 7). On day 10, only infected mosquitoes treated with malaria-associated biogenic amines had significantly higher levels of activity than uninfected mosquitoes (Fig 7). To examine the interaction of treatment with infection status, we used a GLMM (Poisson regression) with these parameters as main effects. Both infection status (χ2 = 4470.19, df = 1, P < 0.0001) and treatment (χ2 = 5650.8, df = 2, P < 0.0001) had significant effects on the activity of *A. stephensi* and the interaction was significant (χ2 = 151.83, df = 2, P < 0.0001). As noted, infection had a negative impact on the interaction (*β* = -0.263, 95% CI [-0.277, -0.250], P < 0.0001) at 4 and 14 days (Fig 7). Both malaria-associated treatment (*β* = 0.267, 95% CI [0.256, 0.279], P < 0.0001) and healthy-associated treatment (*β* = 0.071, 95% CI [0.58, 0.83], p < 0.0001) had a positive effect on the interaction.

**Fig 8:**
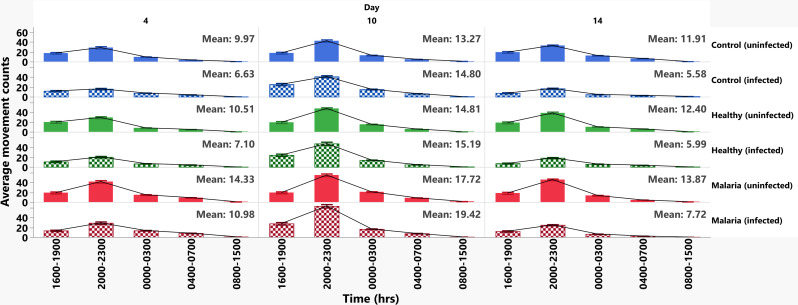
Mean movement counts of uninfected and *P. yoelii*-infected *A. stephensi* provisioned with histamine + 5-HT. The blue (control), green (healthy-treated mosquitoes) and red (malaria-treated mosquitoes) solid and hatched bars represent mean movement counts and standard errors of the mean for uninfected and infected mosquitoes, respectively. The mean movement counts are plotted at three-hour intervals within 24 hours for five biological replicates (uninfected day 4; control n = 48, healthy n = 47, malaria n = 57, uninfected day 10; control n = 48, healthy n = 53, malaria n = 54, uninfected day 14; control n = 51, healthy n = 51, malaria n = 50, infected day 4; control n = 43, healthy n = 44, malaria n = 46, infected day 10; control n = 63, healthy n = 64, malaria n = 70, infected day 14; control n = 34, healthy n = 35, malaria n = 26). Among uninfected and infected groups, *A. stephensi* provisioned with malaria-associated treatment had higher movement counts than the control and healthy-associated treatment groups. Uninfected mosquitoes provided with water (control), healthy-, or malaria-associated treatment exhibited higher activity than infected mosquitoes provided with the same treatment at days 4 and 14 post bloodmeal. Pairwise comparisons of the means were performed with Tukey HSD. P values ≤ 0.05 were considered significant. * P ≤ 0.05, ** P ≤ 0.01, ***P ≤ 0.001, **** P ≤ 0.00001.

To identify the periods with highest activity levels for each timepoint post-bloodmeal, we analyzed the data from Fig 7 in three-hour increments over a 24-hour time period (Fig 8). Across all days and groups, activity levels were highest in late evening (2000–2300 h), followed by early evening (1600–1900 h), midnight (0000–0300 h) and dawn (0400–0700 h; S11 Table). Among infected mosquitoes, activity levels were higher in the malaria-associated treatment group relative to the healthy-associated treatment group from 2000-0700 h at 4 and 10 days post-bloodmeal (Fig 8). At day 14 post-bloodmeal, activity levels were higher in the malaria-associated treatment group relative to the healthy group at 2000–2300 h (Fig 8 and S12 Table). For infected malaria- and healthy-associated treatment groups, mean activity levels for these days were consistent with these observations (day 4 = 10.98 vs 7.10; day 10 = 19.42 vs 15.19; day 14 = 7.72 vs 5.99; Fig 8). Among uninfected mosquitoes, activity levels from 2000-0700 h at 4, 10 and 14 days were also higher in mosquitoes treated with malaria-associated biogenic amines relative to those treated with healthy-associated levels (Fig 8 and S12 Table), consistent with observations in Fig 3. Comparing uninfected and infected females, uninfected females at days 4 and 14 tended to have relatively higher activity levels than infected females in the same treatment group (e.g., malaria vs malaria, healthy vs healthy) and time (Fig 8 and S13 Table). At day 10, however, patterns of activity tended to be reversed as in Fig 7, with mean activity levels trending higher in infected females than uninfected females in all treatment groups (control infected vs control uninfected = 14.80 vs 13.27; healthy infected vs healthy uninfected = 15.19 vs 14.81; malaria infected vs malaria uninfected = 19.42 vs 17.72; Fig 8). Notably, infected females with malaria-associated treatment exhibited higher activity than uninfected females with malaria-associated treatment during the early evening (1600–1900 h) and late evening (2000–2300 h; Fig 8), time periods that appeared to account for the significant difference in infected versus uninfected mosquito activity in the malaria-associated treatment group at day 10 in Fig 7.

## Discussion

We previously reported that ingested histamine and 5-HT at levels comparable to those in the blood of human hosts with severe malaria altered *A. stephensi* reproduction, blood feeding, flight and infection [15]. These behaviors are critical determinants of vectorial capacity of *A. stephensi* and are influenced by circadian rhythms [46,47] and retinal responses [48]. In insects, circadian activity is influenced by lifespan [49–52], temperature [6,53–55], and infection [56] which interact with biogenic amine signaling [16,39]. Prior to our studies, there were no established connections between histaminergic and serotonergic signaling with diel locomotor activity (an output of circadian activity) and retinal responses in *A. stephensi*. Further, there was little to no evidence to support effects of biogenic amine signaling on mosquito locomotor activity across lifespan, at different temperatures, or in the presence of parasite infection. Our findings address these knowledge gaps, revealing that exposure of both uninfected and infected *A. stephensi* to histamine and 5-HT at levels associated with severe malaria exhibited greater and broadened periods of activity than mosquitoes that ingested healthy-associated biogenic amines. These treatment effects were also associated with increased ERG amplitudes at 0800–1100 h relative to 2000–2300 h, time periods that correspond to very low and very high activity, respectively. The effect on locomotor activity was observed across lifespan, at different temperatures, and days post-bloodmeal or infection. These results highlight the potential for *Plasmodium* manipulation of human biosynthesis pathways to increase the likelihood of parasite transmission by *A. stephensi*.

Aging is known to affect key aspects of physiology that underlie insect behavior [57]. As expected, we observed that locomotor activity of uninfected *A. stephensi* declined across lifespan in all treatment groups. This reduction in activity with increasing age is a compensatory mechanism adopted to manage the metabolic costs associated with movement, thereby enhancing the chances of survival [58,59]. The rate of decay in activity was slower, however, in mosquitoes treated with malaria-associated biogenic amines than in those treated with healthy levels. Mosquitoes treated with malaria-associated biogenic amines also had greater locomotor activity levels at weeks 1–4 as compared to controls or mosquitoes treated with healthy-associated levels. This result underscores the significance of gut-brain axis communication in regulating *A. stephensi* diel locomotor activity, an output of circadian rhythms [6,45,58], and regular oscillation of internal clock transcripts genes in mosquito brains [60].

The effect of malaria-associated levels of biogenic amines in slowing the rate of the age-related decline in activity in *A. stephensi* affirms our earlier inference that biogenic amines, in particular histamine, play a role in offsetting the physiological and behavioral declines associated with aging [16]. Hylton *et al.* [61] and Rowley and Graham [62] identified the degeneration of striated flight muscles and alteration of biochemical pathways that inhibit mosquitoes from utilizing glycogen for flight energy as primary causes for reduced movement over lifespan. Given that histamine dose-dependently stimulates H1- and H2 receptor-dependent glycogen breakdown in cultured rat astrocytes [27] and in human endothelial cells [26] and that H1 and H2 receptors are expressed in the midgut of *Anopheles* mosquitoes [63,64], malaria-associated levels of histamine could restimulate glycogenolysis to support higher activity relative to mosquitoes provided with healthy levels of this biogenic amine.

In contrast to the indirect effects of histamine, 5-HT-induced activity of serotonergic neurons in the ventral nerve cord can directly modify the activity of locomotor neural networks [30–32]. A high concentration of 5-HT in serotonergic neurons can decrease the desire for food and inhibit the motor circuit in *Caenorhabditis elegans* [33]. Hence, it is plausible that elevated 5-HT in the healthy-associated treatment could signal a reduction in the urge to feed and inhibition of the motor circuit and locomotor activity at times that should be marked by activity [33]. In contrast to these effects of higher levels of 5-HT, Hersey et al. [65], and Schwörer et al. [66] showed that histamine at high levels inhibits 5-HT release by binding or cross-reacting to 5-HT receptors. Taken together, these observations suggest that elevated histamine levels in the malaria-associated treatment could antagonize the serotonergic signaling pathway, resulting in reduced 5-HT levels and increased motor activity in *A. stephensi*.

Contrary to our expectations for night active mosquitoes, *A. stephensi* provisioned with malaria-associated treatment exhibited more light cycle activity than the healthy-associated treatment group, with significant differences in weeks 2, 4, and 5 with most activity occurring in the early evening (1600–1900 h) before lights off at 2000 h. These mosquitoes exhibited the broadest range of increased activity from early evening (1600–1900 h) to dawn (0400–0700 h) in week 1, higher activity in the early evening (1600–1900 h) in week 3 and late evening (2000–2300 h) and dawn (0400–0700 h) in week 4. Our findings related to *A. stephensi* peak active period (1600–0700 h) do not depart from the reported peak biting time of anophelines in the field [67–70]. However, by broadening the range of peak activity period in week 1, the effects of malaria-associated biogenic amines could support longer flight activity, enabling *A. stephensi* to locate suitable oviposition sites or a second bloodmeal to boost early oocyst development and shorten extrinsic incubation period [71] or to become infected if the first bloodmeal did not result in infection. Increasing light cycle activity and an early evening activity peak in later weeks could also be beneficial to the parasite through increased transmission, posing a greater risk to humans. Early uptake of *Plasmodium* gametocytes (1600–1900 h) and the resulting interaction with ambient temperature could also improve the likelihood of mosquito infection [72]. Finally, early evening activity could reduce the effects of indoor interventions such as insecticide-treated nets on infected *A. stephensi*, increasing host-vector interactions and the probability of transmission [73,74].

The compound eyes of mosquitoes are crucial to detecting movement and potential hosts [75] and convey photic information to the central circadian clock for synchronization with environmental light cycles [76]. Mosquitoes possess a range of photoreceptors that respond differently to various wavelengths of light at different times of the day. The observed reduction in electrical activity of the retina in *A. stephensi* at light onset (0800–1100 h) compared to dark onset (2000–2300 h) in the control group aligns with its known crepuscular behavior and corroborates previous findings on *A. gambiae* [77]. However, enhanced retinal responses were observed at light onset (0800–1100 h) in *A. stephensi* treated with malaria-associated biogenic amines relative to those measured at the onset of darkness (2000–2300 h), a complete reversal of light sensitivity relative to control. The circadian clock orchestrates the time-dependent modulation of photoreceptor sensitivity and synaptic transmission through the rhythmic expression of key genes involved in visual transduction pathways (e.g., rhodopsin genes *GPROP1*, *ninaA*) and neurotransmitter signaling (e.g., *SNMP* gene) [76,77], which could explain this observation. The increased retinal response at light onset in the malaria-associated treatment group suggests that these signals extend the duration of activity, which could increase the likelihood of a subsequent bloodmeal for a species like *A. stephensi* that feeds both indoors and outdoors [78]. In support, malaria-associated biogenic amine treatment could change the temporal profile of photoreceptor sensitivity in *A. stephensi* or influence diel behaviors through molecular clock timing. For example, Baik et al. [60] showed that circadian neuron circuits modulate light-evoked attraction and avoidance behaviors driven by molecular clock timing. In *Drosophila*, 5-HT receptor 1B expressed in clock neurons modulates molecular and behavioral responses of the clock to light [79], while HisCl1 histamine receptors act on photoreceptors to synchronize circadian behavioral rhythms [24].

Temperature influences circadian activity patterns of diverse insect species [6,80,81] and both histaminergic and serotonergic signaling are known to be altered by temperature in a variety of organisms [23,37,82]. Temperature alone and the interaction of temperature with malaria-associated biogenic amine treatment altered the locomotor activity of uninfected *A. stephensi*. Specifically, uninfected *A. stephensi* treated with malaria-associated biogenic amines exhibited greater activity at all temperatures compared to the healthy treatment group. At low temperatures (21 °C), uninfected *A. stephensi* exhibited late evening peaks, whereas at high temperatures (31–34 °C), the peaks were during early evening. Mosquitoes provisioned with malaria-associated treatment exhibited peak activity at 31 °C, whereas peak activity in the healthy-associated treatment group was at 28 °C (our colony-rearing temperature). At 31 °C, the malaria-associated treatment group also exhibited the broadest period of increased activity from early evening to early morning (1600–1100 h) compared to the healthy-associated group.

Histamine can modulate temperature preference and control insect tolerance to low and high temperatures [20]. Serotonin effects, on the other hand, are dependent on neural input and may cause motor relaxation at lower or activation at higher temperatures to regulate locomotor activity [37]. Treatment with malaria-associated histamine and 5-HT increased *A. stephensi* activity up to 31 °C, which could allow niche expansion along with earlier evening activity and a longer period of heightened activity. With recent work predicting that *A. stephensi* suitability for *P. falciparum* infection will extend across a very broad temperature range (15.3 °C to 37.2 °C) [83], our observations support an increased likelihood of human-vector contact and *Plasmodium* transmission, particularly at higher temperatures. These findings affirm serious challenges to malaria elimination campaigns in the context of climate-induced temperature shifts, particularly in epidemic prone zones [84].

Our data affirm that *Plasmodium* infection modulates diel locomotor activity of *A. stephensi*. These effects could translate into biting patterns that could either enhance parasite development or increase transmission to human hosts [81,82]. Infection on day 4 (early oocysts) and day 14 (mature sporozoites) reduced mosquito activity relative to uninfected mosquitoes regardless of treatment. This corroborates flight data collected from flight mill studies [85] and actinograms [56] on *P. cynomolgi-* and *P. yoelii-*infected *A. stephensi*. We presume these decreases could derive from the diversion of energy to repair damage to tissue epithelial or basement membranes or to fuel defense responses to infection [56,86]. Typically, epithelial cells are damaged during ookinete migration from the midgut lumen to the basal lamina, where they remain sessile as early oocysts [87,88]. Sporozoites, on the other hand, cause basement membrane damage when traversing secretory cells, secretory cavities, and the larger central lumen to enter the salivary duct of *A. stephensi* [86]. As oocysts are sessile, they mature and acquire nutrients from the host [56,85,89], competing with the host for resources. For successful transmission to humans, malaria parasites and the mosquito host must strike a balance for survival. The impact of competing nutritional needs to ensure survival could explain the increased activity of infected *A. stephensi* relative to uninfected mosquitoes at the peak of oocyst maturation at 10 days post-infection, with malaria-associated biogenic amine treatment reinforcing and enhancing this activity. Indeed, this increased activity occurs during early evening (1600–1900 h) and late evening (2000–2300 h), perhaps enhancing periods of greatest activity to increase the likelihood of feeding.

In general, the effects of infection amplified malaria-associated biogenic amine treatment across all days post-infection, suggesting that parasite manipulation of the human host results in altered mosquito behavior to potentiate transmission. Closer examination of *A. stephensi* behavior over time revealed that malaria-associated biogenic amine treatment increased activity levels from late evening to dawn (2000–0700 h) at days 4 and 10 post-bloodmeal for infected mosquitoes. In uninfected mosquitoes, malaria-associated biogenic amine treatment also increased activity levels from late evening to dawn (2000–0700 h) at days 4, 10, and 14 days post-bloodmeal. If increased activity were translated to increased host feeding, this extended activity could increase the likelihood of infection of uninfected *A. stephensi* and the likelihood of parasite transmission by infected *A. stephensi*.

Our findings on the tripartite interactions (parasite-human, human-vector, parasite-vector) help to define parasite-induced alterations in blood histamine and 5-HT and their effects on *A. stephensi* as investments by the parasite in infection and development within the vector to sustain its own transmission. The responsiveness of *A. stephensi* gut-brain axis to ingested biogenic amines suggests an open system defined by ingested blood factors, temperature, and parasites that modulate the vectorial capacity of *A. stephensi* [63]. We believe that attractive targeted bait station strategies for the delivery of specific histaminergic or serotonergic signaling antagonists to anopheline mosquitoes could synergize with current vector and infection control strategies for improved malaria control in endemic regions.

## Materials and methods

### Ethics statement

The study was conducted under approval by the Institutional Animal Care and Use Committee of the University of Idaho (protocol IACUC-2023-08, approved 27 February 2023).

### Mosquito rearing

*Anopheles stephensi* (Indian wild type strain) was maintained in an Arthropod Containment Level 2 (ACL2)/Biosafety Level 2 (BSL2) insectary based at University of Idaho Laboratory Animal Research Facility (LARF) as described [14–16]. For regular husbandry, adults and immature stages were maintained at 28 °C, with 80% humidity and a light–dark cycle 12:12 (0800–2000). A day prior to the start of an experiment, 2–3-day old female mosquitoes were aspirated from the main colony and housed in labeled 2-liter polypropylene cartons with mesh screening and then moved to respective incubators. All experiments were performed in automated Darwin Chambers Company environmental chambers set at 80% humidity, and a light–dark cycle 12:12 (lights on from 0800–2000 h), with pre-set temperatures appropriate for the experiment.

### Provisioning of histamine and 5-HT and artificial bloodmeals to *A. stephensi* for lifespan studies

One hundred 2–3-day old *A. stephensi* were aspirated into each of three separate cartons and maintained at 28 °C, with 80% humidity and a light–dark cycle 12:12 (0800–2000). Each carton was provided with sugar cubes and soaked cotton pads with one of the three biogenic amine treatments: healthy-associated biogenic amines (1 nM histamine + 1.5 µM 5-HT in water), malaria-associated biogenic amines (10 nM histamine + 0.15 µM 5-HT in water), or water-only from day 0 to day 3 as described [15]. This procedure was repeated weekly across lifespan. The numbers of dead mosquitoes were recorded daily for the lifespan study beginning on day 1 after treatment. Thirty minutes before the first bloodmeal, sugar cubes and soaked cotton balls were removed from the cartons, the numbers of dead mosquitoes were recorded, and eight *A. stephensi* from each carton were aspirated individually into Pyrex glass tubes for diel locomotor activity monitoring. These eight aspirated mosquitoes were excluded from survival analysis. Bloodmeal delivery to the remaining mosquitoes in the cartons was completed as described [14–16]. Briefly, a bloodmeal of washed human red blood cells and serum at a ratio of 1:1 vol:vol was delivered for 30 min through glass bell jar feeders heated to 37 °C by continuously circulating water. Cartons of bloodfed mosquitoes were kept in an incubator at 28 °C with 80% humidity and a light–dark cycle 12:12 (lights on from 0800–2000 h). Egg cups were added on the second day after blood feeding for oviposition. The procedure (3-day provisioning followed by a bloodmeal) was repeated weekly throughout the lifespan. The study was replicated with four separate cohorts of *A. stephensi*.

### Monitoring diel locomotor activity of uninfected *A. stephensi* provisioned with histamine and 5-HT over lifespan

Locomotor activity was monitored using a Locomotor Activity Monitor 2 system (LAM25H; TriKinetics Inc., Waltham, MA USA). The LAM25H system is composed of activity monitors each holding 32 Pyrex glass tubes (2.5 cm × 12.5 cm) and fitted with nine pairs of infrared beams bisecting the middle of each tube [9,76,90,91]. Eight female *A. stephensi* were aspirated weekly from each carton after 3 days of provisioning with water and biogenic amines and prior to bloodmeal delivery. Each mosquito was transferred into vertically stationed Pyrex glass tubes on LAM25H monitors with labeled rows, then covered at the bottom and top with a thin layer of cotton wool plug soaked in 10% sucrose, allowing for unrestricted air movement. The LAM25H monitors with mosquitoes from each treatment group maintained at 28 °C with 80% humidity and a light–dark cycle 12:12 (0800–2000 h). The experiment was set up 2 h before data collection to allow the mosquitoes to acclimate. Mosquito movement was detected as beam breaks and recorded as counts per minute in 24 h by *Drosophila* Activity Monitor (DAM) System 311 software [90]. The procedure was repeated for five consecutive weeks across the lifespan and replicated with four separate cohorts of *A. stephensi*.

### Measuring retinal responses of uninfected *A. stephensi* during the initial stages of light and dark phases following provisioning of histamine and 5-HT

Female *A. stephensi* from 5–7 days old that were provisioned as above for 3 days with malaria-associated and healthy-associated histamine and 5-HT were used for electroretinogram (ERG) recordings [48]. To immobilize the mosquitoes for ERG recordings, they were briefly anesthetized at 4 °C, after which their legs and wings were removed. Mosquitoes were then mounted ventrally on slides using double-sided tape to hold the abdomen, thorax, and cervix in place, and secured with Bondic glue to ensure immobilization. Mosquitoes were dark-adapted for 30 min before stimulation.

The reference electrode, an electrolytically sharpened tungsten wire (Tungsten rod 0.04 in x 3 in, A-M Systems, Sequim, WA USA), was carefully inserted into the center of the compound eye using a thin layer of electronic gel (Spectra 360, Parker Laboratories, NJ, USA). The recording electrode with a resistance of 1–10 MΩ (Quartz glass with Filament, 100-70-10, Sutter Instrument, Novato, CA, USA) was pulled using a micropipette puller (P-2000, Sutter Instrument, Novato, CA, USA). It was then loaded with mosquito saline solution and carefully placed on the surface of the contralateral eye. The mosquito saline solution was prepared by dissolving 150 mM NaCl, 3.35 mM KCl, 1.7 mM CaCl₂, 0.95 mM MgCl₂, 25 mM HEPES, and 1.8 mM NaHCO₃ in Milli-Q water, adjusting the pH to 7.0 with 1 M NaOH, and adding 3 mM sucrose for experiments.

In the experiments, mosquitoes were exposed to 1-second light pulses from a xenon light source (SLS205, Thorlabs, Newton, NJ USA), ranging from 320 to 700 nm in 10 nm intervals. Each treatment group included 7–11 mosquitoes, collected from over five different cohorts. Each pulse was separated by 30 seconds of darkness (i.e., no light). Each wavelength was presented randomly and tested twice per mosquito. Wavelengths were controlled using a fiber optic scanning monochromator (MonoScan 2000, Ocean Insight, Orlando, FL, USA). The light was delivered through optical fibers (QP600–025-UV, Ocean Optics, Orlando, FL, USA) and its intensity was uniformly set to light intensity = 1 × 10^15^ μW/cm^2^/nm by customized neutral density filters (Thorlabs, Newton, NJ, USA) across all wavelengths. For accurate scaling of the irradiance for each visual stimulus, a cosine-corrected spectrophotometer (USB2000+, Ocean Optics, Orlando, FL USA) was placed immediately next to the recording preparation for calibration. The experiments lasted less than 1.5 h per mosquito. Recordings commenced before the light transitions and concluded no later than 3 h after the lights turned on or off.

The signal was captured using a preamplifier (Universal AC/DC Probe, Gain of 10X, Ockenfels Syntech GmbH, Germany) and digitized with a data acquisition system (Axon Digidata 1550B, Molecular Devices, San Jose, CA USA). Data analysis was performed in R (R Core Team, 2021) using code adapted from Peach and Blake [92]. Statistical analyses were conducted using Python version 3.11 (Python Software Foundation, 2022) and the statsmodels package version 0.14.0 [93].

ERG data were analyzed using Generalized Linear Mixed Models (GLMMs; Python function: statsmodels.api.MixedLM) for data analysis due to their flexibility in handling fixed and random effects, accounting for complex data structure. We evaluated several configurations, including models with and without interaction terms between the fixed effects, as well as models with and without categorizing wavelengths into small groups. Model selection was based on the Akaike Information Criterion (AIC). The model with the lowest AIC, which did not categorize wavelengths and excluded interaction terms involving wavelengths, was chosen so that we can rigorously assess the effects of the variable while minimizing overfitting. The final model was specified as ERG Amplitude ~ Biogenic Amine Treatments * Light Cycle + Wavelengths. In this model, the response variable was the ERG amplitude. Each variable accounting for the fixed effects was treated as a categorical variable. Random effects were calculated for each of the 7–11 mosquitoes from each treatment group, collected from over five different cohorts as random intercepts. The statistical significance of the fixed effects was evaluated.

### Monitoring diel locomotor activity of uninfected *A. stephensi* at different temperatures following provisioning of histamine and 5-HT

Twenty-five 2-day old female *A. stephensi* (n = 25) were aspirated into each of 15 separate cartons, and three cartons were each placed in five different incubators with temperatures set at 21, 24, 28, 31, or 34 °C, respectively, with 80% humidity and a light–dark cycle 12:12 (lights on from 0800–2000 h). The mosquitoes in the three cartons per incubator were provisioned with treatments or water as described above. At the end of the third day of provisioning, eight female *A. stephensi* from each of the groups and each temperature were transferred individually from their respective cartons into vertically stationed Pyrex glass tubes on LAM25H monitors as described above. The LAM25H monitors with eight mosquitoes from each group were placed in their respective incubators at 21 °C, 24 °C, 28 °C, 31 °C, and 34 °C, and locomotor activity was monitored as described above. The procedure was replicated using three separate cohorts of *A. stephensi*.

### Mouse infection and monitoring

To assess the interaction between biogenic amines and infection on the diel locomotor activity of *A. stephensi*, mosquitoes were allowed to feed on uninfected female 9–11-week-old CD-1 mice (Envigo, Indianapolis, IN) or female CD-1 mice infected with *Plasmodium yoelii yoelii* 17XNL (*P. yoelii*). All mice were housed in ventilated micro-isolator caging and provided with food and water *ad libitum*. The Institutional Animal Care and Use Committee of the University of Idaho approved all procedures (IACUC Protocol 2023-08, approved on 2/27/2023). Mouse infections were performed as described [94]. Briefly, mice (n = 4) were infected by intraperitoneal injection (IP) with 150 µL of *P. yoelii*-infected red blood cells (1 × 10^6^) at day 0. Three uninfected mice were used as controls. Microscopic examination of parasitemia on Giemsa-stained thin blood smears was performed daily starting from the second day post infection (PI). On days 3–4 PI, male gametocytes were microscopically evaluated and exflagellation events were counted on slides with a fresh drop of blood. Three mice with similar exflagellation events and groups of uninfected mice (controls) were anesthetized using 50 mg/kg of ketamine and 5 mg/kg of xylazine in sterile saline as previously described [15].

### Provisioning of histamine and 5-HT to *A. stephensi* prior to blood feeding on mice

A total of 120 2-day-old *A. stephensi* were aspirated into each of six labeled 2 L polypropylene cartons with mesh screening. Three cartons were tagged as uninfected and the other three cartons were tagged as infected, and each set of three mosquito cartons was provisioned with biogenic amine treatments or water, as described above, and placed in an incubator at 24°C. Three days after treatment, sugar cubes and cotton balls soaked in treatment or water were removed from the cartons. The three *P. yoelii*-infected mice were each placed on top of three 2 L polypropylene cartons labeled as the infected group, while uninfected mice were placed on top of three 2 L polypropylene cartons labeled as the uninfected group, and mosquitoes were allowed to feed for 30 min. After a successful bloodmeal, partially fed and non-fed mosquitoes from each carton were removed and killed by freezing for 48 h. The fully fed mosquitoes were maintained in an incubator set at 24 °C. To confirm mosquito infection, 8–16 midguts were dissected at 11 days post-infection (dpi) and stained with mercurochrome for oocyst counting. Salivary glands were dissected from 8-16 mosquitoes per group at 15 dpi, and the number of sporozoites was scored as previously described in [16].

### Monitoring diel locomotor activity of *P. yoelii*-infected *A. stephensi*

Locomotor activity was monitored on days 4, 10 and 14 post-feeding on infected or uninfected mice. The selected days coincided with parasite developmental stages in the midgut and salivary glands. Specifically, day 4 coincided with early midgut oocyst development, day 10 corresponded to late midgut oocysts and early sporozoite release from mature oocysts, while day 14 corresponded to the completion of sporozoite invasion of the salivary glands at which time sporozoites are infectious [95]. Diel locomotor activity of female *A. stephensi* from each treatment group that fed on infected or uninfected mice was monitored at 24 °C as described above. On days 11 and 15, following the completion of 24 h activity monitoring, mosquitoes were dissected to confirm infection status. Activity monitoring data from mosquitoes that were not successfully infected were discarded. These procedures were replicated using five separate cohorts of *A. stephensi*.

### Statistical analyses for activity data

Movement counts per minute recorded within 24 hours by the LAM25H monitors were retrieved from DAM System 311 software and verified using the DAMFileScan113 software before conversion from WordPad version (.txt format) to Microsoft Excel Worksheet (.xlsx) format. Data recorded before the onset of the experiment at 1900 h were discarded as these represented hours of acclimatization for *A. stephensi*. Outlier analysis was performed to identify and eliminate individual data points that deviated significantly from the rest of the observations. The effect of the interaction between treatment and age, temperature or infection status on *A. stephensi* movement counts was determined using a Poisson Generalized Linear Mixed Model (GLMM). The Generalized Linear Model with a negative binomial response and a pairwise comparison (Tukey HSD) was used to compare movement count variations across experimental groups in all experiments. Pairwise comparisons were done using Tukey’s post-hoc HSD to compare the diel locomotor activity between control, healthy- and malaria-associated treatment groups across each individual experimental setup. Movement count data were converted into binary to examine the effects of biogenic amines and infection status on the number of active periods during the 12 h light and dark periods and during the periods noted above. Movement counts per minute were converted to a binary format where activity per minute was recorded as “1” and the lack of activity or resting was recorded as “0”. The Chi-square test was used to calculate the variation in the number of active periods during the 12 h light and dark periods within and between the groups, and to compare active peak hours between control, healthy-, and malaria-associated biogenic amine treated, infected and uninfected mosquitoes. Data visualization and analysis were conducted using GraphPad Prism version 10.2.0, JMP Pro version 17.1.0, and SPSS version 25.

## Supporting information

S1 Fig *Anopheles* feeding outcomes from likely human hosts. Malaria vectors can obtain blood from uninfected or healthy individuals (green), individuals with asymptomatic parasitemia (dark green) or those with severe malaria (red). **A.** When mosquitoes feed on the blood of a healthy individual, they would ingest healthy blood levels of biogenic amines (1 nM H + 1.5 µM 5-HT) and remain uninfected. **B.** Optionally, if they feed on asymptomatic individuals with parasitemia and transmissible gametocytes, they may become infected or remain uninfected while ingesting healthy levels of blood histamine and 5-HT. **C.** If they feed on individuals with severe malaria and transmissible gametocytes, they may become infected or remain uninfected, while ingesting blood containing malaria-associated levels of biogenic amines (10 nM H + 0.15 µM 5-HT). This figure was created in BioRender, https://BioRender.com/y92e226.
(TIF)

S2 Fig Median survival of uninfected *A. stephensi* provisioned with histamine + 5-HT. Mosquitoes were provisioned weekly with healthy-associated 1 nM histamine (H) + 1.5 µM 5-HT (green bars), malaria-associated 10 nM H + 0.15 µM 5-HT (red bars), or water-soaked cotton balls (blue bars) from day 0 to day 3, followed by bloodmeals with no supplement. N = 4 biological replicates, Kruskal-Wallis test with Dunn’s multiple comparisons test revealed no significant differences.
(TIF)

S3 Fig Mean proportion of weekly active dark and light periods of uninfected *A. stephensi* provisioned with histamine + 5-HT. The bars represent the mean proportion of active periods ± standard error of the mean of 4 biological replicates at 12 hours light period of week 1: control (n = 27, 0.18 ± 0.01), healthy (n = 31, 0.17 ± 0.01), malaria (n = 27, 0.16 ± 0.01), week 2: control (n = 31, 0.13 ± 0.01), healthy (n = 28, 0.10 ± 0.01), malaria (n = 29, 015 ± 0.01), week 3: control (n = 31, 0.07 ± 0.01), healthy (n = 32, 0.04 ± 0.01), malaria (n = 30, 0.10 ± 0.01), week 4: control (n = 29, 0.07 ± 0.01), healthy (n = 29, 0.05 ± 0.01), malaria (n = 30, 0.09 ± 0.01), and week 5: control (n = 24, 0.08 ± 0.01), healthy (n = 25, 0.03 ± 0.01), malaria (n = 27, 0.10 ± 0.01). At 12 hours dark period of week 1: control (n = 27, 0.48 ± 0.02), healthy (n = 31, 0.61 ± 0.02), malaria (n = 27, 0.51 ± 0.02), week 2: control (n = 31, 0.46 ± 0.02), healthy (n = 28, 0.34 ± 0.02), malaria (n = 29, 0.63 ± 0.02), week 3: control (n = 31, 0.34 ± 0.02), healthy (n = 32, 0.40 ± 0.02), malaria (n = 30, 0.28 ± 0.02), week 4: control (n = 29, 0.16 ± 0.01), healthy (n = 29, 0.11 ± 0.01), malaria (n = 30, 0.32 ± 0.02), and week 5: control (n = 24, 0.07 ± 0.01), healthy (n = 25, 0.20 ± 0.02), malaria (n = 27, 0.12 ± 0.01). Light-shaded bars reflect the 12-hour light period (L), whereas dark bars represent the 12-hour dark period (D). Mosquitoes treated with malaria-associated biogenic amines (10 nM H + 0.15 µM 5-HT; red bars) and controls (blue bars) had significantly more active periods in dark cycles (dark bars) compared to light cycles (light bars) in weeks 1, 2, 3, and 4. Mosquitoes treated with healthy-associated biogenic amines (1 nM H + 1.5 µM 5-HT) were significantly more active in dark cycles (dark green bars) compared to light cycles (light green bars) in weeks 1, 2, 3 and 5. Chi-square test. P values ≤ 0.05 were considered significant. * P ≤ 0.05, ** P ≤ 0.01, ***P ≤ 0.001, **** P ≤ 0.00001.
(TIF)

S4 Fig The density plot of movement counts among treatment groups, by week, and time discretized into 3-hour categories. The X-axis represents log of movement counts while the Y-axis represents densities.
(TIF)

S5 Fig Graphical output of results from movement counts data modeled as a function of treatment levels, week, and time discretized into 3-hour categories. The X-axis represents the incident rate ratios (IRRs) while the Y-axis represents weeks, treatment and the 3-hour categories. The IRR quantifies the effect of a predictor variable on the incidence rate or count data for Generalized Linear Models (GLMs) with a Poisson distribution. The IRR represents a multiplicative increase or decrease in the incidence rate based on a change in the predictor variable. It is calculated as the ratio of the incidence rates between two groups or for a unit change in the predictor variable. An IRR that is greater than 1 indicates an increase in the incidence rate associated with an increase in the predictor variable, while an “IRR < 1” indicates a decrease. In comparison to movement counts in week 1, mosquitoes in week 2 exhibited the highest IRR of 0.93. Among treatment groups, the malaria-associated treatment group had the highest IRR of 1.34 compared to the healthy-associated treatment group, while during 2000–2300 h, mosquitoes exhibited the highest IRR of 1.62 relative to the time frame 0000–0300 h.
(TIF)

S6 Fig Graphical output of the frequency of distribution of movement count data by weeks demonstrating zero inflation. The X-axis represents recorded movement count data after 24 hours while the Y-axis represents the distribution of each count data.
(TIF)

S7 Fig The graphical output of conditional and Zero Inflated Model results from movement counts data modeled as a function of treatment levels, week, and time discretized into 3-hour categories. The X-axis represents the incident rate ratios (IRRs) while the Y-axis represents weeks, treatment and the 3-hour categories. The conditional figure is a representation of active period or movement counts while the zero-inflated figure is a representation of resting or period of inactivity.
(TIF)

S8 Fig Mean proportion of active periods of uninfected *Anopheles stephensi* in light and dark cycles at different temperatures. The bars represent the mean proportion of active periods ± standard error of the mean of 3 biological replicates at light period at 21 °C: control (n = 23, 0.11 ± 0.01), healthy (n = 22, 0.09 ± 0.01), malaria (n = 24, 0.07 ± 0.01), 24 °C: control (n = 24, 0.11 ± 0.01), healthy (n = 24, 0.05 ± 0.01), malaria (n = 24, 0.13 ± 0.01), 28 °C: control (n = 23, 0.25 ± 0.02), healthy (n = 21, 0.21 ± 0.02), malaria (n = 22, 0.20 ± 0.01), 31 °C: control (n = 24, 0.26 ± 0.02), healthy (n = 22, 0.12 ± 0.01), malaria (n = 24, 0.28 ± 0.02), and at 34 °C: control (n = 21, 0.13 ± 0.01), healthy (n = 19, 0.11 ± 0.01), malaria (n = 22, 0.25 ± 0.02). At dark period at 21 °C: control (n = 23, 0.58 ± 0.02), healthy (n = 22, 0.47 ± 0.02), malaria (n = 24, 0.60 ± 0.02), 24 °C: control (n = 24, 0.59 ± 0.02), healthy (n = 24, 0.52 ± 0.02), malaria (n = 24, 0.72 ± 0.02), 28 °C: control (n = 23, 0.65 ± 0.02), healthy (n = 21, 0.78 ± 0.02), malaria (n = 22, 0.74 ± 0.02), 31 °C: control (n = 24, 0.57 ± 0.02), healthy (n = 22, 0.44 ± 0.02), malaria (n = 24, 0.87 ± 0.01), and at 34°C: control (n = 21, 0.48 ± 0.02), healthy (n = 19, 0.55 ± 0.02), malaria (n = 22, 0.61 ± 0.02). Light bars reflect 12-hour light periods (L), whereas the dark bars represent 12-hour dark periods (D) at each temperature. Chi-square test. P values ≤ 0.05 were considered significant. * P ≤ 0.05, ** P ≤ 0.01, ***P ≤ 0.001, **** P ≤ 0.00001.
(TIF)

S9 Fig The density plot of movement counts among treatment groups, by temperature range, and time discretized into 3-hour categories. The X-axis represents log of movement counts while the Y-axis represents densities.
(TIF)

S10 Fig Graphical output of results from movement counts data modeled as a function of treatment levels, temperature ranges, and time discretized into 3-hour categories. The X-axis represents the incident rate ratios (IRRs) while the Y-axis represents weeks, treatment and the 3-hour categories. The IRR quantifies the effect of a predictor variable on the incidence rate or count data for Generalized Linear Models (GLMs) with a Poisson distribution. The IRR represents a multiplicative increase or decrease in the incidence rate based on a change in the predictor variable. It is calculated as the ratio of the incidence rates between two groups or for a unit change in the predictor variable. An IRR that is greater than 1 indicates an increase in the incidence rate associated with an increase in the predictor variable, while an “IRR < 1” indicates a decrease. In comparison to movement counts at 21 °C, mosquitoes at higher temperature exhibited the higher IRR. Among treatment groups, the malaria-associated treatment group had the highest IRR of 1.64 compared to the healthy-associated treatment group, while during 2000–2300 h, mosquitoes exhibited the highest IRR of 4.22 relative to the time frame 0000–0300 h.
(TIF)

S11 Fig Graphical output of the frequency of distribution of movement count data showing zero inflation. The X-axis represents recorded movement count data after 24 hours while the Y-axis represents the distribution of each count data.
(TIF)

S12 Fig The graphical output of conditional and Zero Inflated Poisson model results from movement counts data modeled as a function of treatment levels, temperature range, and time discretized into 3-hour categories. The model accounts for the excess zeroes in the data using a two-parts model, a logit for the probability of zeroes and a Poisson GLM for the counts. We use the same systemic functional model, with treatment, temperature and duration as the factors for both the probability of excess zeroes and the Poisson distributed counts. The X-axis represents the incident rate ratios (IRRs) while the Y-axis represents weeks, treatment and the 3-hour categories. The conditional figure is a representation of active period or movement counts while the zero-inflated figure is a representation of resting or period of inactivity.
(TIF)

S1 Table Simple Survival Analysis (Kaplan-Meier) of uninfected *A. stephensi* provisioned weekly with malaria-associated biogenic amine treatment [10nM histamine (H) + 0.15 μM 5-HT], healthy-associated treatment [1nM H + 1.5 μM 5-HT] or water as a control in soaked cotton balls. (DOCX)

S2 Table Pairwise comparison (Tukey HSD) of the effect of provisioned malaria-associated biogenic amine treatment (10nM H + 0.15 μM 5-HT), healthy-associated treatment (1nM H + 1.5 μM 5-HT), or water (control) on locomotor activity across lifespan. (DOCX)

S3 Table Summary table for computed p-values using the Chi-square test in comparison of the number of active periods between treatment groups under light and dark duration. Treatments included malaria-associated biogenic amine treatment (10nM H + 0.15 μM 5-HT), healthy-associated treatment (1nM H + 1.5 μM 5-HT), or water (control).
(DOCX)

S4 Table Pairwise comparison (Tukey HSD) of the effect of provisioned malaria-associated biogenic amine treatment (10nM H + 0.15 μM 5-HT), healthy-associated treatment (1nM H + 1.5 μM 5-HT), or water (control) on locomotor activity over period across lifespan. (DOCX)

S5 Table Output of conditional and zero-inflated model results from movement counts data modeled as a function of treatment levels, week, and time discretized into 3-hour categories. (DOCX)

S6 Table Generalized linear mixed model results for ERG responses of uninfected *A. stephensi*, assessing the effects of biogenic amine treatments (“Treat”: healthy = 1nM H + 1.5 μM 5-HT, malaria = 10nM H + 0.15 μM 5-HT, control = water), time of experiments (“Time”: am = 0800–1100 h, pm = 2000–2300 h), and wavelengths (“Wvlength”: light stimuli from 320 to 700 nm in 10 nm increments). (DOCX)

S7 Table Pairwise comparisons (Tukey HSD) of locomotor activity patterns between time periods by treatment group and temperature. Treatments included malaria-associated biogenic amine treatment (10nM H + 0.15 μM 5-HT), healthy-associated treatment (1nM H + 1.5 μM 5-HT), or water (control).
(DOCX)

S8 Table Pairwise comparisons (Tukey HSD) of locomotor activity patterns between treatment group by period and temperature. Treatments included malaria-associated biogenic amine treatment (10nM H + 0.15 μM 5-HT), healthy-associated treatment (1nM H + 1.5 μM 5-HT), or water (control).
(DOCX)

S9 Table Summary table for computed p-values using the Chi-square test in comparison of the number of active periods between temperature across treatments under light and dark duration. Treatments included malaria-associated biogenic amine treatment (10nM H + 0.15 μM 5-HT), healthy-associated treatment (1nM H + 1.5 μM 5-HT), or water (control).
(DOCX)

S10 Table Output of conditional and zero-inflated model results from movement counts data modeled as a function of treatment levels, week, and time discretized into 3-hour categories. (DOCX)

S11 Table Pairwise comparisons (Tukey HSD) of locomotor activity levels (i) between infected and uninfected mosquitoes by treatment and time, and (ii) comparison between time periods to determine activity patterns among treatment groups of infected and uninfected mosquitoes at days 4, 10, and 14 post-bloodmeal. Treatments included malaria-associated biogenic amine treatment (10nM H + 0.15 μM 5-HT), healthy-associated treatment (1nM H + 1.5 μM 5-HT), or water (control).
(DOCX)

S12 Table Pairwise comparisons (Tukey HSD) of locomotor activity levels between treatments among infected and uninfected mosquitoes at days 4, 10, and 14 post-bloodmeal. Treatments included malaria-associated biogenic amine treatment (10nM H + 0.15 μM 5-HT), healthy-associated treatment (1nM H + 1.5 μM 5-HT), or water (control).
(DOCX)

S13 Table Pairwise comparisons (Tukey HSD) of locomotor activity levels between uninfected and infected mosquitoes among treatments at days 4, 10, and 14 post-bloodmeal. Treatments included malaria-associated biogenic amine treatment (10nM H + 0.15 μM 5-HT), healthy-associated treatment (1nM H + 1.5 μM 5-HT), or water (control).
(DOCX)

S1 Data Source data files. Excel files with separate sheets containing the numerical data for Figs 1–3, 5–8, and S1–S8. (RAR)
