# Supplementary figures and images for: Regulation of diel locomotor activity and retinal responses of Anopheles stephensi by ingested histamine and serotonin is temperature- and infection-dependent
Source: PLoS Pathog. 2025 Apr 28;21(4):e1013139. doi: 10.1371/journal.ppat.1013139 (PMC12058162; doi:10.1371/journal.ppat.1013139)

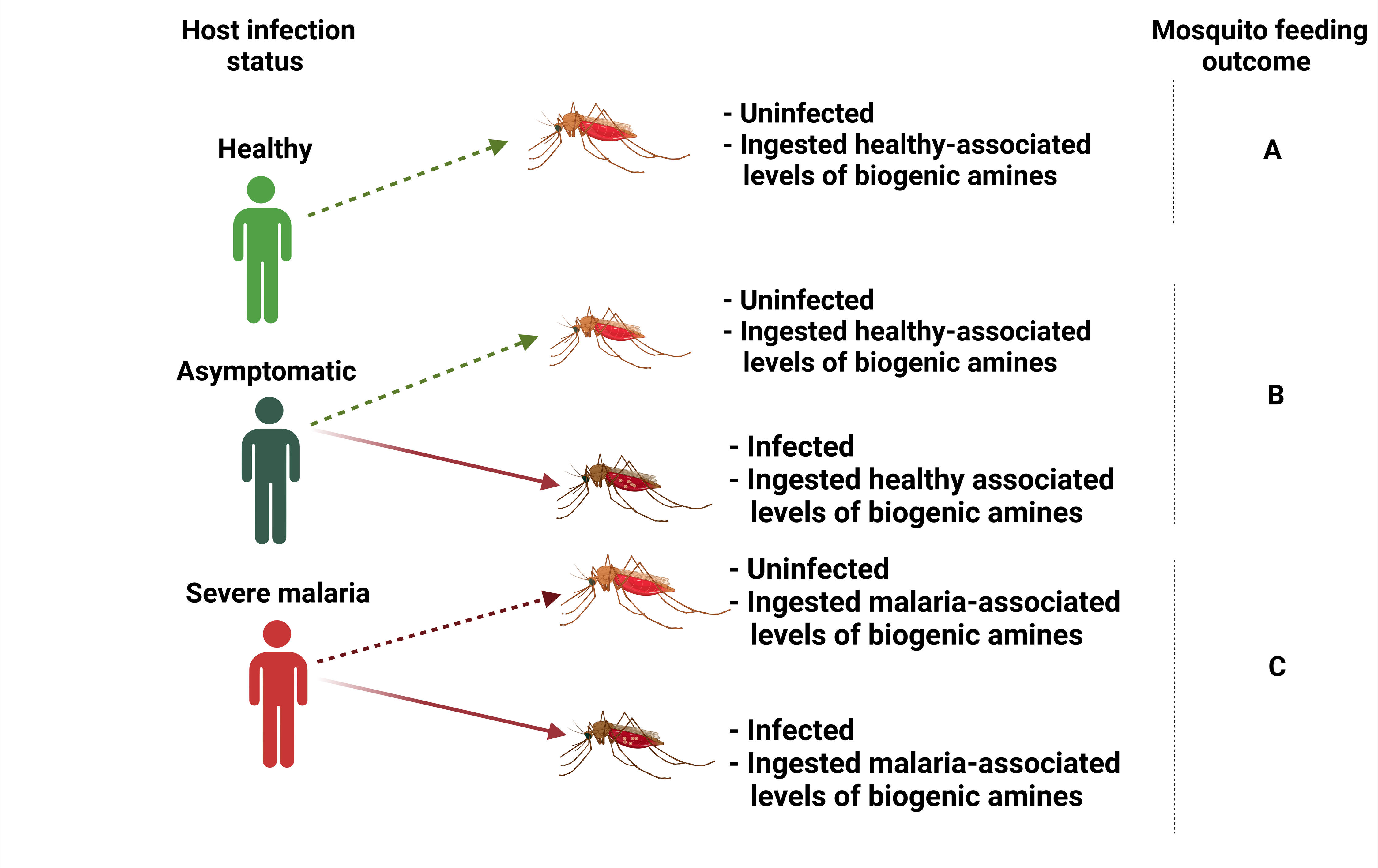

Supplement: S1 Fig — Malaria vectors can obtain blood from uninfected or healthy individuals (green), individuals with asymptomatic parasitemia (dark green) or those with severe malaria (red). A. When mosquitoes feed on the blood of a healthy individual, they would ingest healthy blood levels of biogenic amines (1 nM H + 1.5 µM 5-HT) and remain uninfected. B. Optionally, if they feed on asymptomatic individuals with parasitemia and transmissible gametocytes, they may become infected or remain uninfected while ingesting healthy levels of blood histamine and 5-HT. C. If they feed on individuals with severe malaria and transmissible gametocytes, they may become infected or remain uninfected, while ingesting blood containing malaria-associated levels of biogenic amines (10 nM H + 0.15 µM 5-HT). This figure was created in BioRender, https://BioRender.com/y92e226. (TIF) [file ppat.1013139.s001.tif]

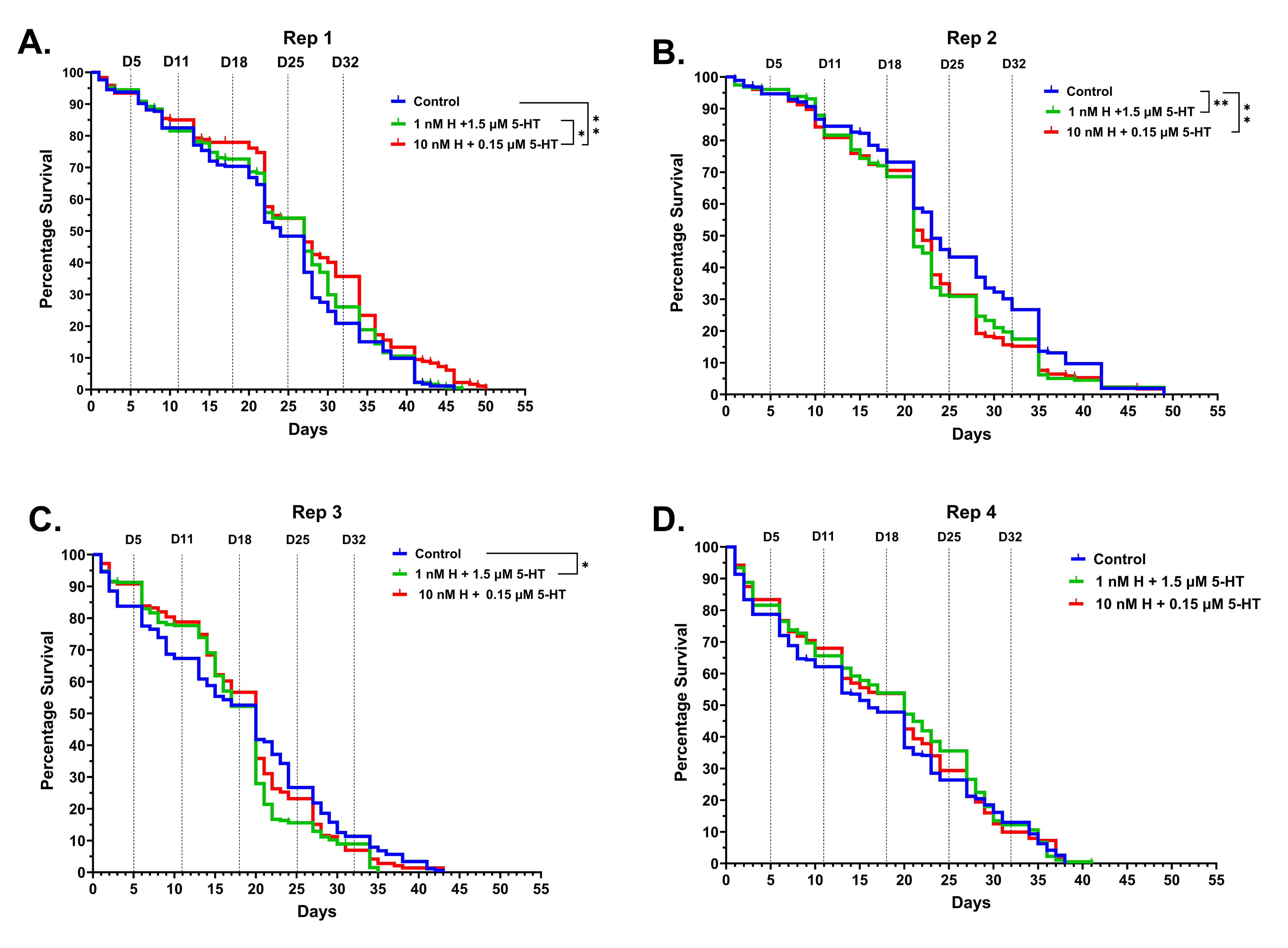

Supplement: S2 Fig — Mosquitoes were provisioned weekly with healthy-associated 1 nM histamine (H) + 1.5 µM 5-HT (green bars), malaria-associated 10 nM H + 0.15 µM 5-HT (red bars), or water-soaked cotton balls (blue bars) from day 0 to day 3, followed by bloodmeals with no supplement. N = 4 biological replicates, Kruskal-Wallis test with Dunn’s multiple comparisons test revealed no significant differences. (TIF) [file ppat.1013139.s002.tif]

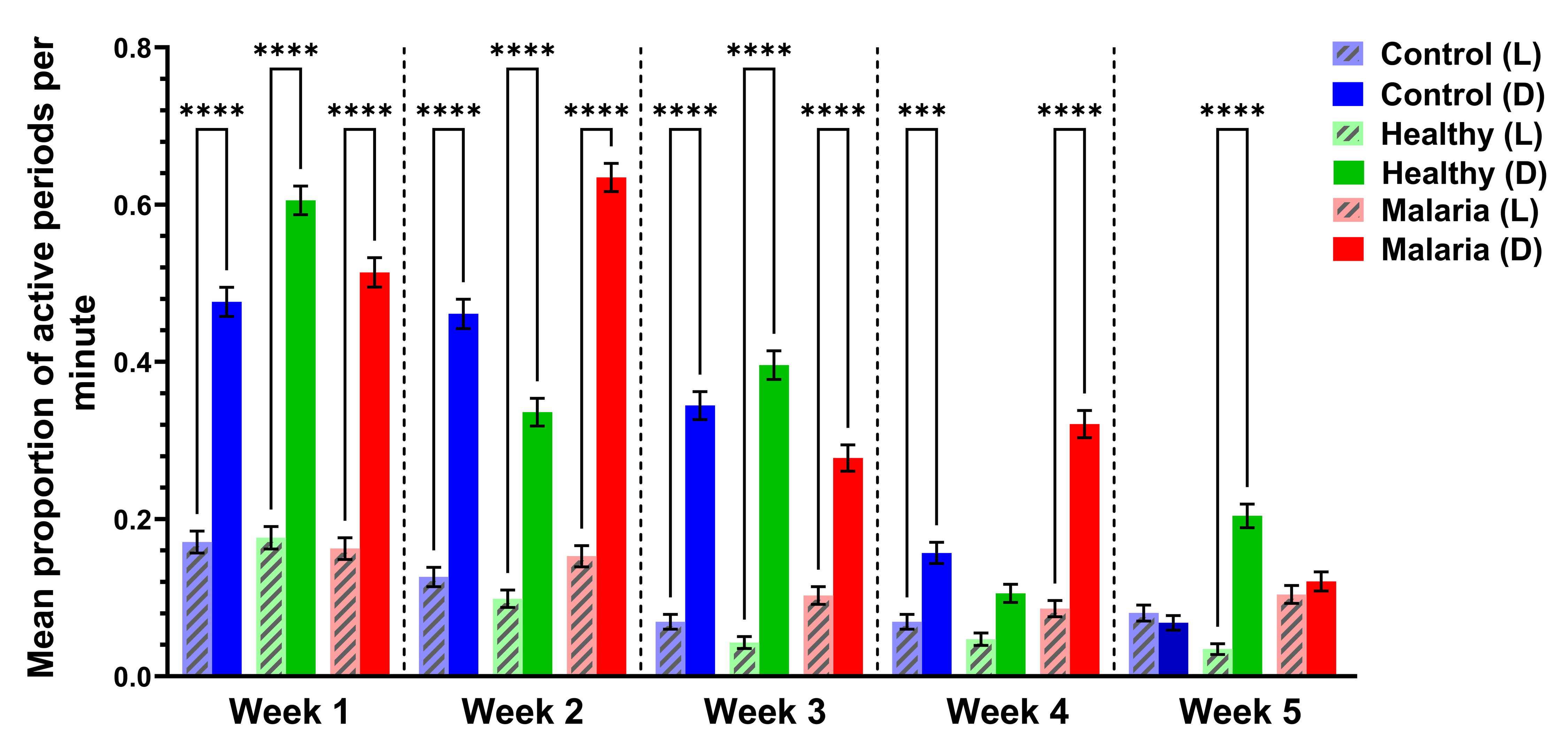

Supplement: S3 Fig — The bars represent the mean proportion of active periods ± standard error of the mean of 4 biological replicates at 12 hours light period of week 1: control (n = 27, 0.18 ± 0.01), healthy (n = 31, 0.17 ± 0.01), malaria (n = 27, 0.16 ± 0.01), week 2: control (n = 31, 0.13 ± 0.01), healthy (n = 28, 0.10 ± 0.01), malaria (n = 29, 015 ± 0.01), week 3: control (n = 31, 0.07 ± 0.01), healthy (n = 32, 0.04 ± 0.01), malaria (n = 30, 0.10 ± 0.01), week 4: control (n = 29, 0.07 ± 0.01), healthy (n = 29, 0.05 ± 0.01), malaria (n = 30, 0.09 ± 0.01), and week 5: control (n = 24, 0.08 ± 0.01), healthy (n = 25, 0.03 ± 0.01), malaria (n = 27, 0.10 ± 0.01). At 12 hours dark period of week 1: control (n = 27, 0.48 ± 0.02), healthy (n = 31, 0.61 ± 0.02), malaria (n = 27, 0.51 ± 0.02), week 2: control (n = 31, 0.46 ± 0.02), healthy (n = 28, 0.34 ± 0.02), malaria (n = 29, 0.63 ± 0.02), week 3: control (n = 31, 0.34 ± 0.02), healthy (n = 32, 0.40 ± 0.02), malaria (n = 30, 0.28 ± 0.02), week 4: control (n = 29, 0.16 ± 0.01), healthy (n = 29, 0.11 ± 0.01), malaria (n = 30, 0.32 ± 0.02), and week 5: control (n = 24, 0.07 ± 0.01), healthy (n = 25, 0.20 ± 0.02), malaria (n = 27, 0.12 ± 0.01). Light-shaded bars reflect the 12-hour light period (L), whereas dark bars represent the 12-hour dark period (D). Mosquitoes treated with malaria-associated biogenic amines (10 nM H + 0.15 µM 5-HT; red bars) and controls (blue bars) had significantly more active periods in dark cycles (dark bars) compared to light cycles (light bars) in weeks 1, 2, 3, and 4. Mosquitoes treated with healthy-associated biogenic amines (1 nM H + 1.5 µM 5-HT) were significantly more active in dark cycles (dark green bars) compared to light cycles (light green bars) in weeks 1, 2, 3 and 5. Chi-square test. P values ≤ 0.05 were considered significant. * P ≤ 0.05, ** P ≤ 0.01, ***P ≤ 0.001, **** P ≤ 0.00001. (TIF) [file ppat.1013139.s003.tif]

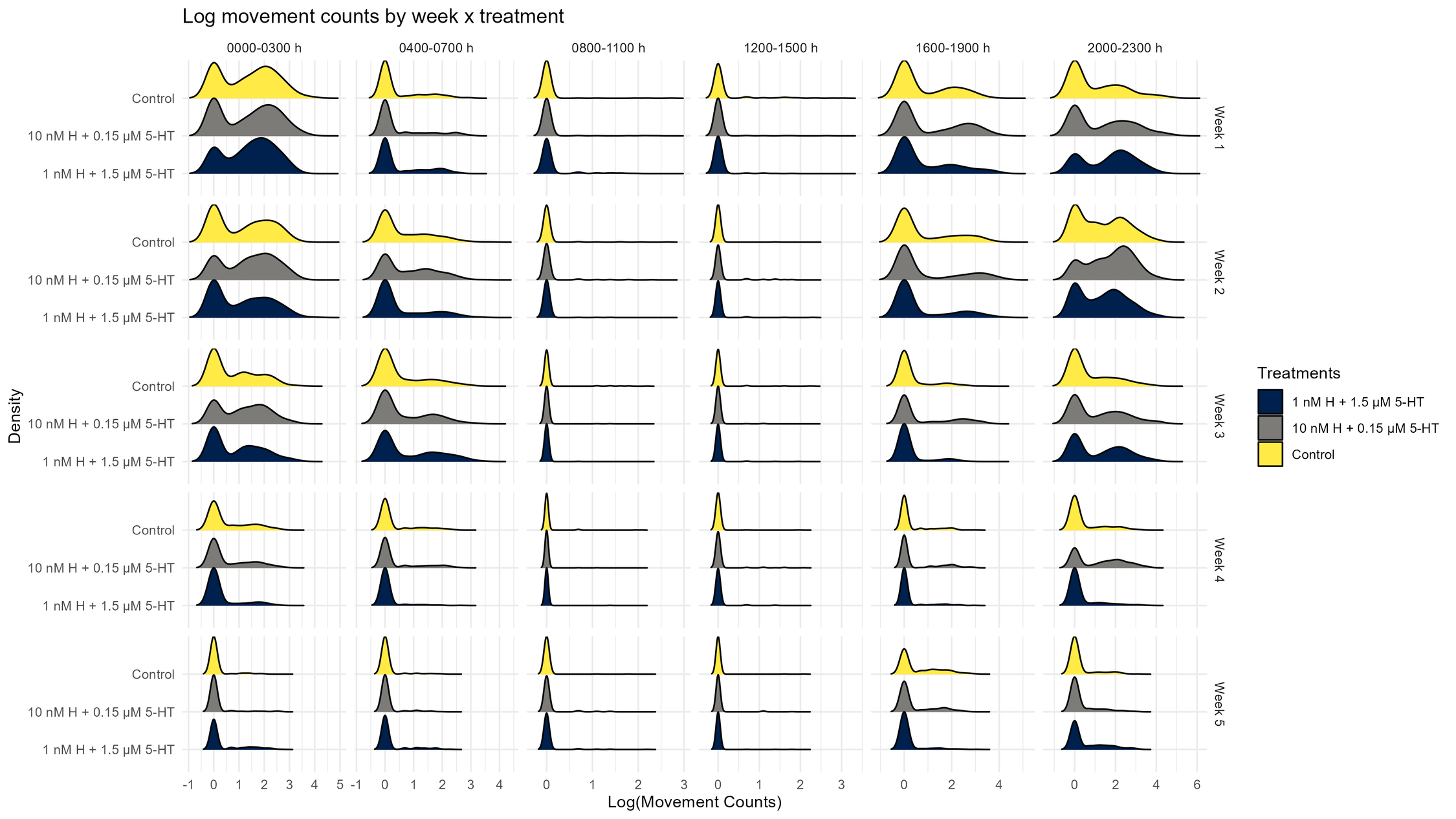

Supplement: S4 Fig — The X-axis represents log of movement counts while the Y-axis represents densities. (TIF) [file ppat.1013139.s004.tif]

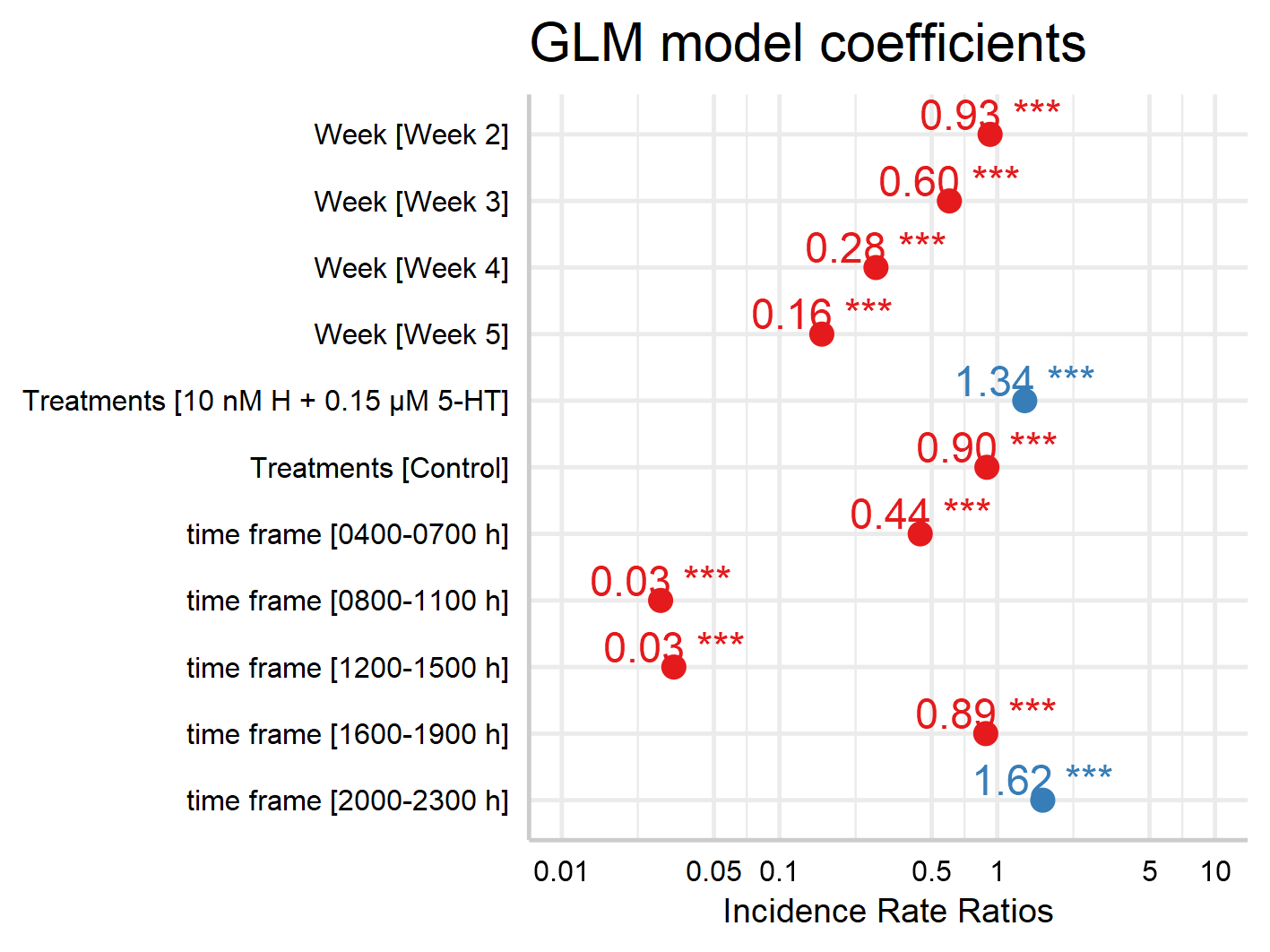

Supplement: S5 Fig — The X-axis represents the incident rate ratios (IRRs) while the Y-axis represents weeks, treatment and the 3-hour categories. The IRR quantifies the effect of a predictor variable on the incidence rate or count data for Generalized Linear Models (GLMs) with a Poisson distribution. The IRR represents a multiplicative increase or decrease in the incidence rate based on a change in the predictor variable. It is calculated as the ratio of the incidence rates between two groups or for a unit change in the predictor variable. An IRR that is greater than 1 indicates an increase in the incidence rate associated with an increase in the predictor variable, while an “IRR < 1” indicates a decrease. In comparison to movement counts in week 1, mosquitoes in week 2 exhibited the highest IRR of 0.93. Among treatment groups, the malaria-associated treatment group had the highest IRR of 1.34 compared to the healthy-associated treatment group, while during 2000–2300 h, mosquitoes exhibited the highest IRR of 1.62 relative to the time frame 0000–0300 h. (TIF) [file ppat.1013139.s005.tif]

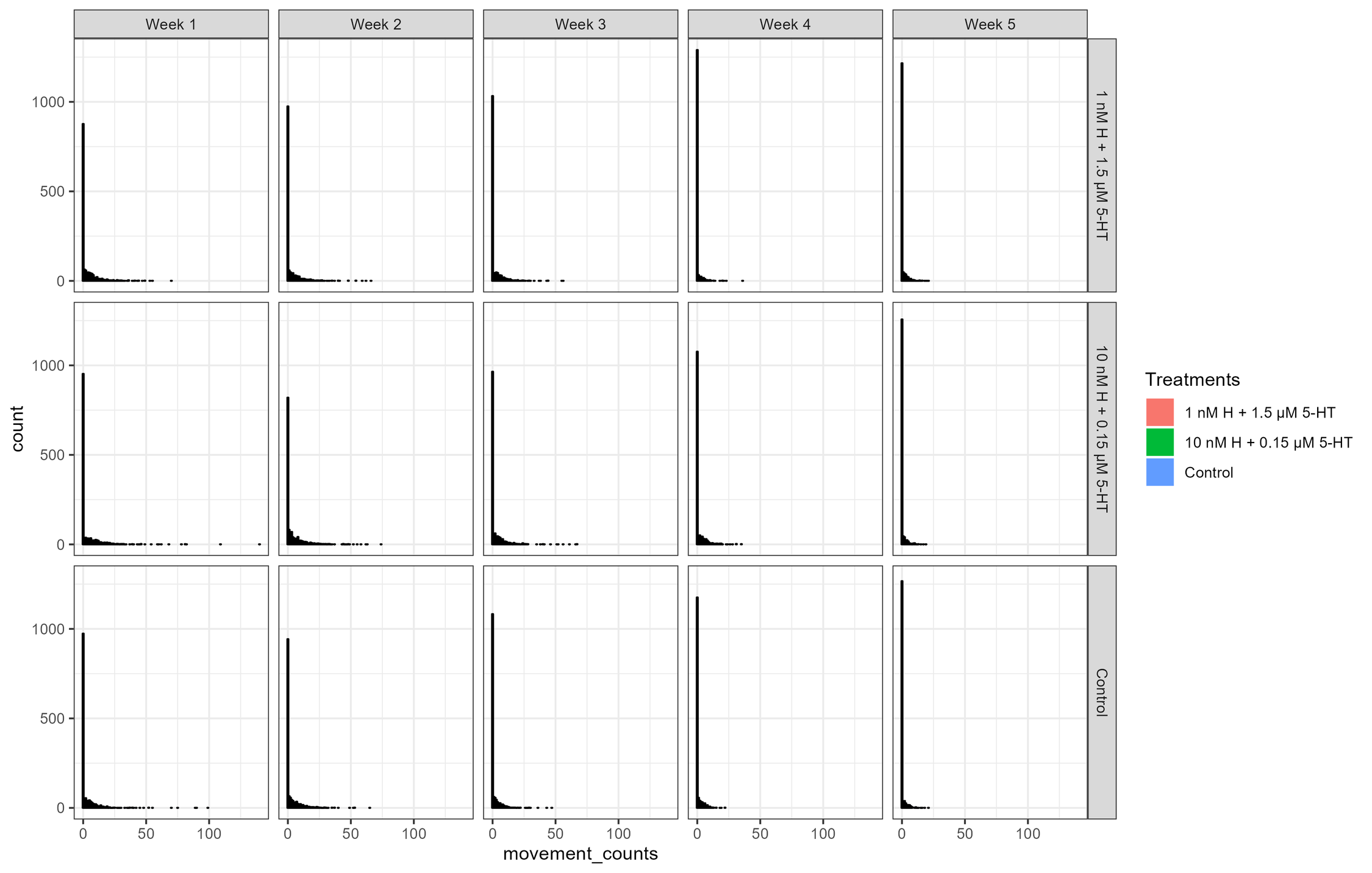

Supplement: S6 Fig — The X-axis represents recorded movement count data after 24 hours while the Y-axis represents the distribution of each count data. (TIF) [file ppat.1013139.s006.tif]

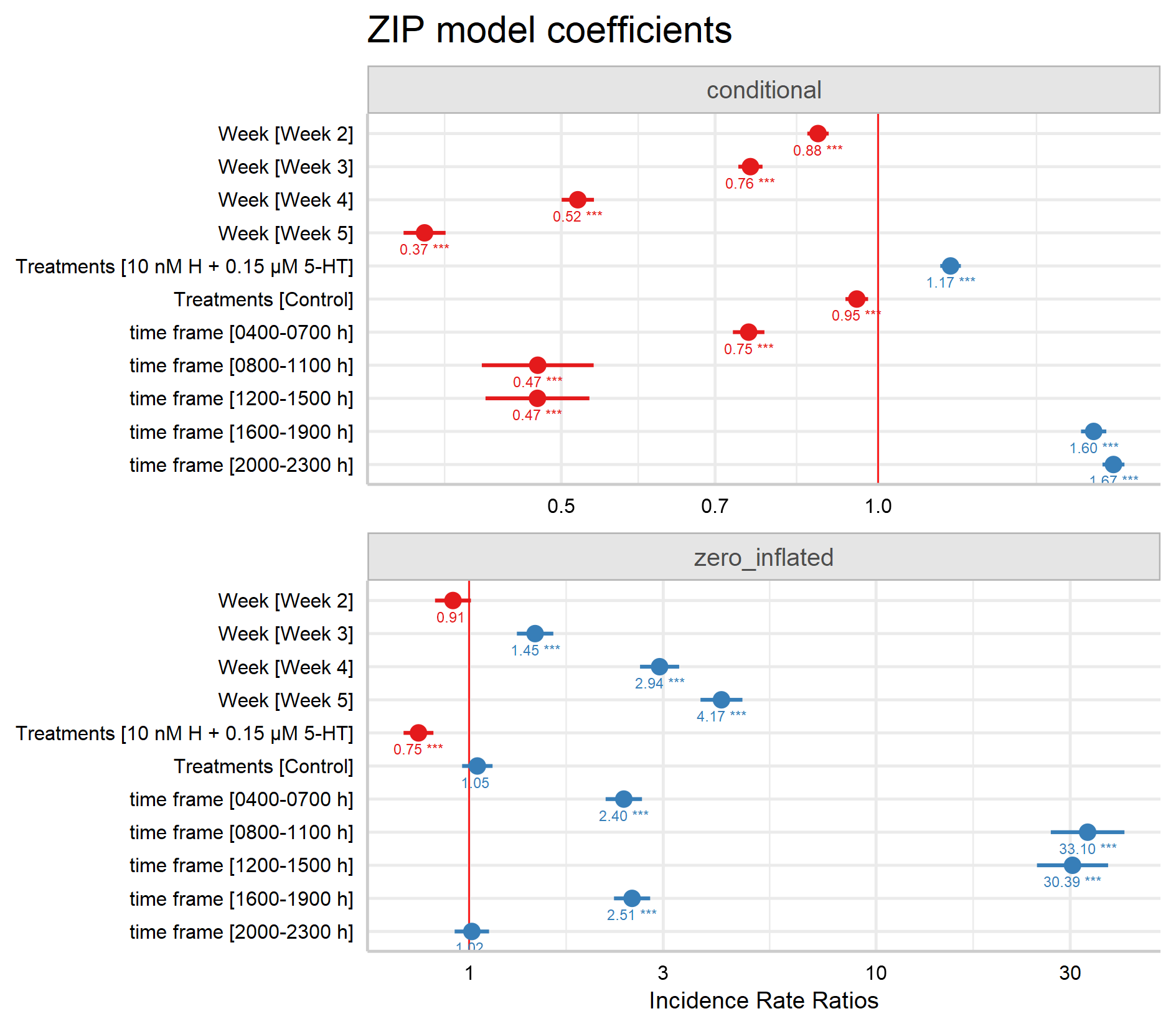

Supplement: S7 Fig — The X-axis represents the incident rate ratios (IRRs) while the Y-axis represents weeks, treatment and the 3-hour categories. The conditional figure is a representation of active period or movement counts while the zero-inflated figure is a representation of resting or period of inactivity. (TIF) [file ppat.1013139.s007.tif]

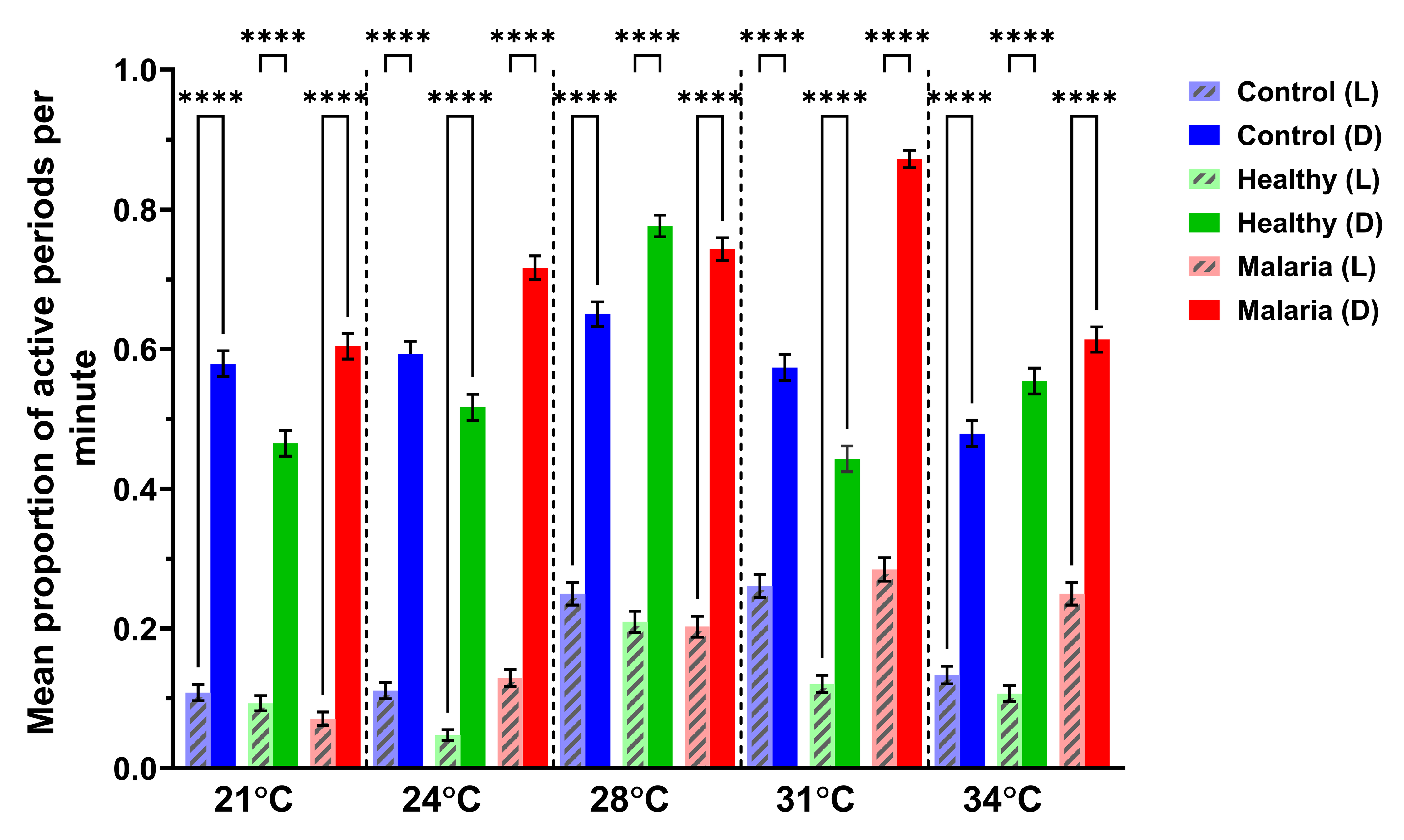

Supplement: S8 Fig — The bars represent the mean proportion of active periods ± standard error of the mean of 3 biological replicates at light period at 21 °C: control (n = 23, 0.11 ± 0.01), healthy (n = 22, 0.09 ± 0.01), malaria (n = 24, 0.07 ± 0.01), 24 °C: control (n = 24, 0.11 ± 0.01), healthy (n = 24, 0.05 ± 0.01), malaria (n = 24, 0.13 ± 0.01), 28 °C: control (n = 23, 0.25 ± 0.02), healthy (n = 21, 0.21 ± 0.02), malaria (n = 22, 0.20 ± 0.01), 31 °C: control (n = 24, 0.26 ± 0.02), healthy (n = 22, 0.12 ± 0.01), malaria (n = 24, 0.28 ± 0.02), and at 34 °C: control (n = 21, 0.13 ± 0.01), healthy (n = 19, 0.11 ± 0.01), malaria (n = 22, 0.25 ± 0.02). At dark period at 21 °C: control (n = 23, 0.58 ± 0.02), healthy (n = 22, 0.47 ± 0.02), malaria (n = 24, 0.60 ± 0.02), 24 °C: control (n = 24, 0.59 ± 0.02), healthy (n = 24, 0.52 ± 0.02), malaria (n = 24, 0.72 ± 0.02), 28 °C: control (n = 23, 0.65 ± 0.02), healthy (n = 21, 0.78 ± 0.02), malaria (n = 22, 0.74 ± 0.02), 31 °C: control (n = 24, 0.57 ± 0.02), healthy (n = 22, 0.44 ± 0.02), malaria (n = 24, 0.87 ± 0.01), and at 34°C: control (n = 21, 0.48 ± 0.02), healthy (n = 19, 0.55 ± 0.02), malaria (n = 22, 0.61 ± 0.02). Light bars reflect 12-hour light periods (L), whereas the dark bars represent 12-hour dark periods (D) at each temperature. Chi-square test. P values ≤ 0.05 were considered significant. * P ≤ 0.05, ** P ≤ 0.01, ***P ≤ 0.001, **** P ≤ 0.00001. (TIF) [file ppat.1013139.s008.tif]

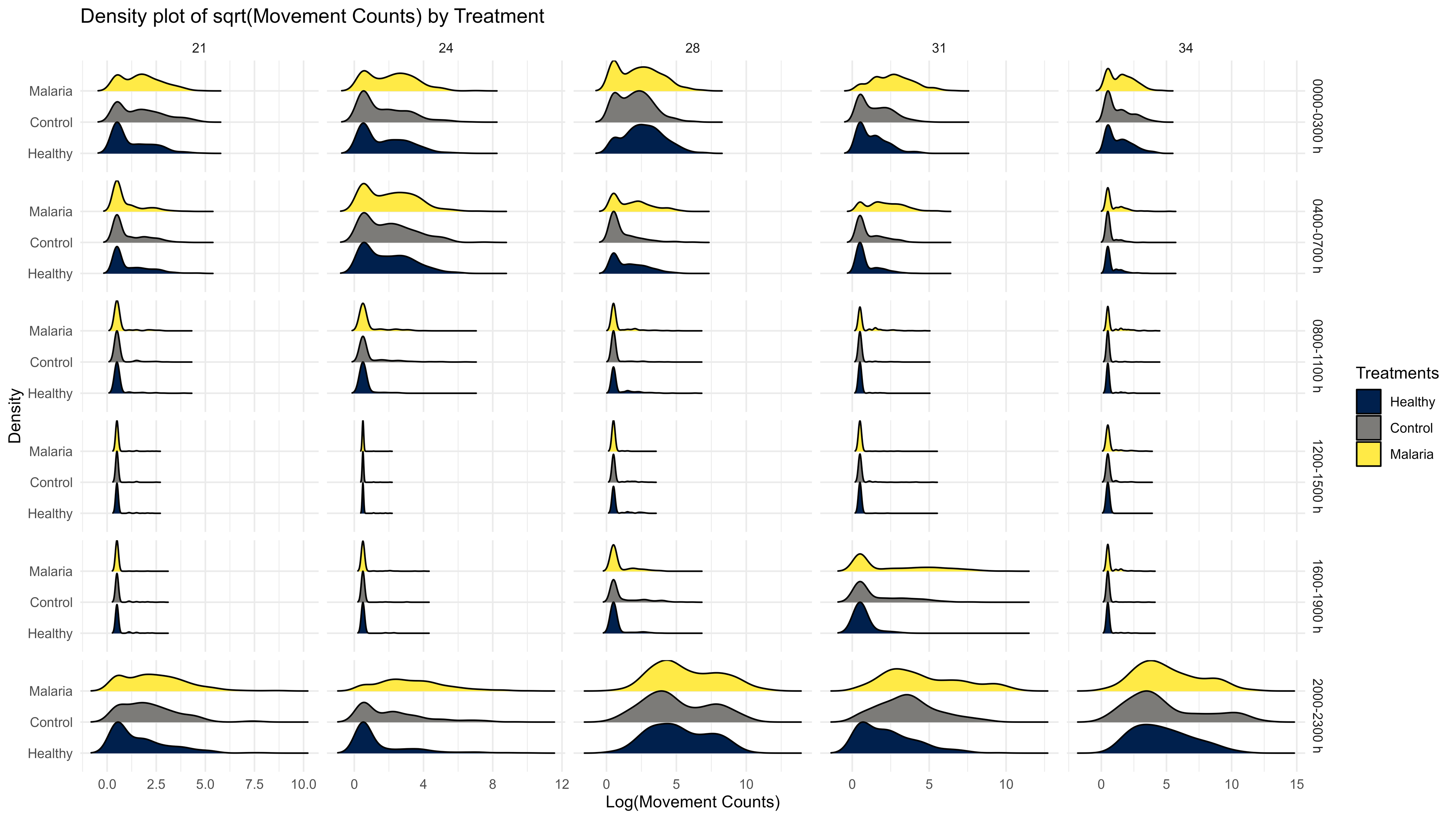

Supplement: S9 Fig — The X-axis represents log of movement counts while the Y-axis represents densities. (TIF) [file ppat.1013139.s009.tif]

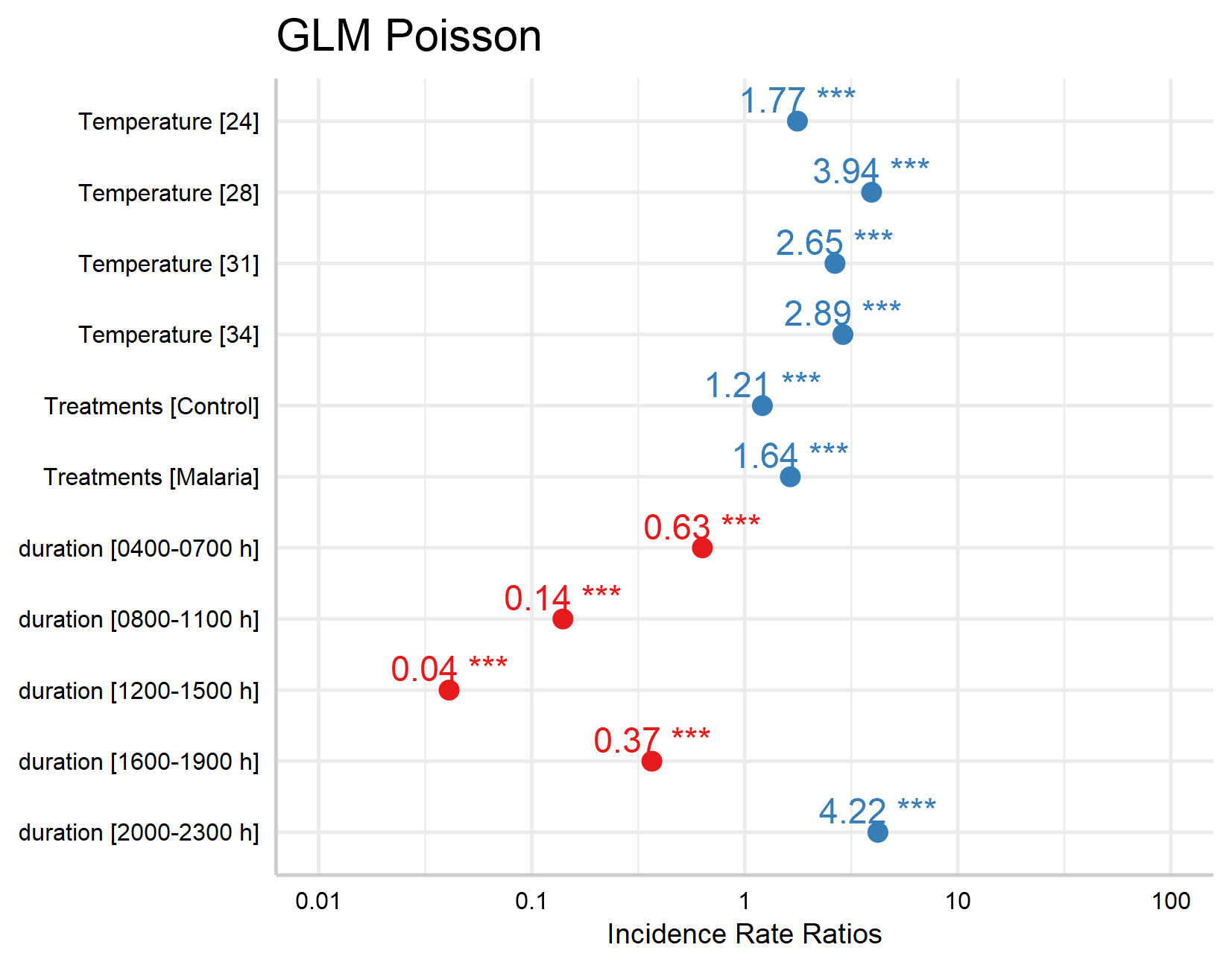

Supplement: S10 Fig — The X-axis represents the incident rate ratios (IRRs) while the Y-axis represents weeks, treatment and the 3-hour categories. The IRR quantifies the effect of a predictor variable on the incidence rate or count data for Generalized Linear Models (GLMs) with a Poisson distribution. The IRR represents a multiplicative increase or decrease in the incidence rate based on a change in the predictor variable. It is calculated as the ratio of the incidence rates between two groups or for a unit change in the predictor variable. An IRR that is greater than 1 indicates an increase in the incidence rate associated with an increase in the predictor variable, while an “IRR < 1” indicates a decrease. In comparison to movement counts at 21 °C, mosquitoes at higher temperature exhibited the higher IRR. Among treatment groups, the malaria-associated treatment group had the highest IRR of 1.64 compared to the healthy-associated treatment group, while during 2000–2300 h, mosquitoes exhibited the highest IRR of 4.22 relative to the time frame 0000–0300 h. (TIF) [file ppat.1013139.s010.tif]

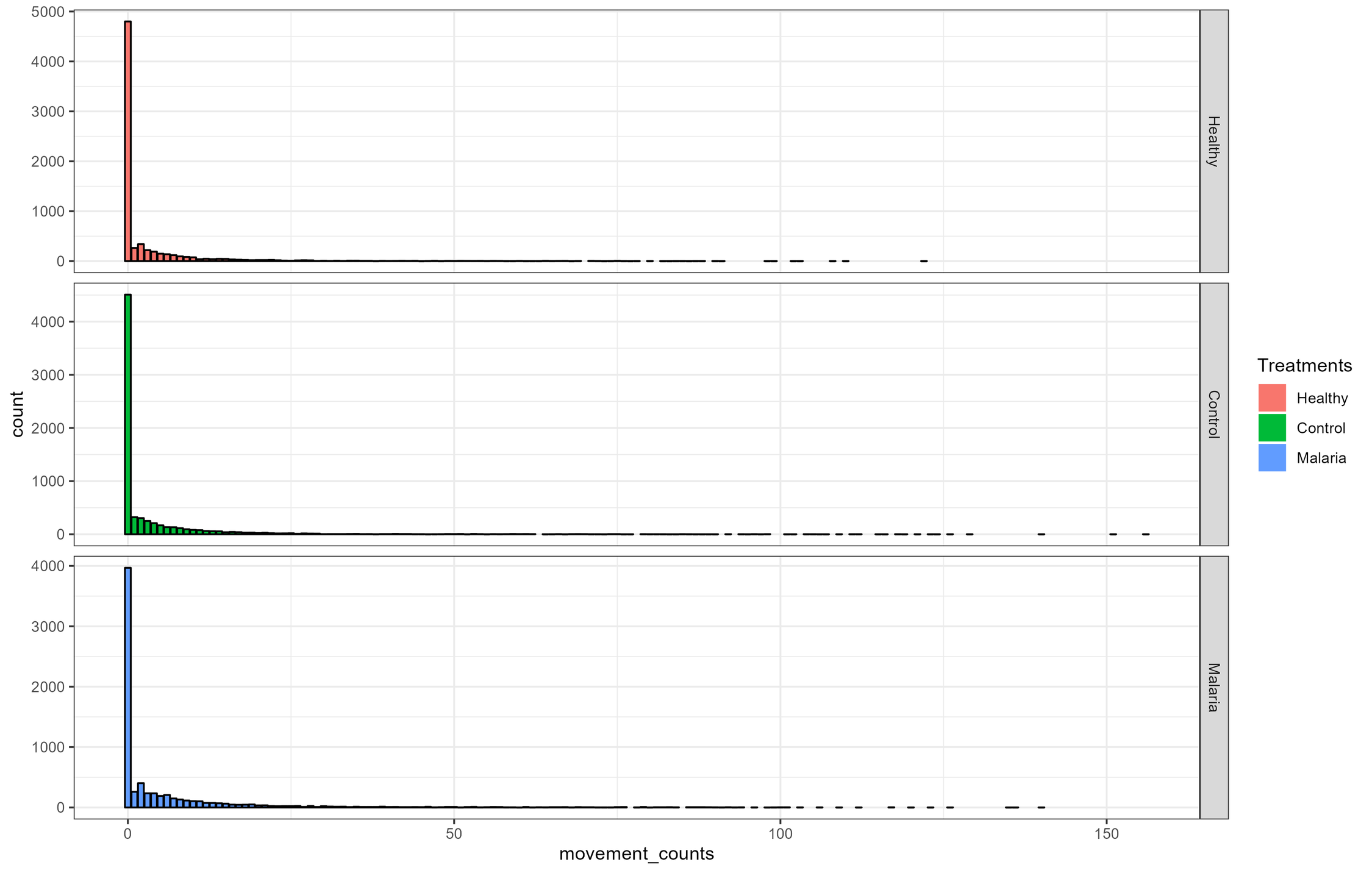

Supplement: S11 Fig — The X-axis represents recorded movement count data after 24 hours while the Y-axis represents the distribution of each count data. (TIF) [file ppat.1013139.s011.tif]

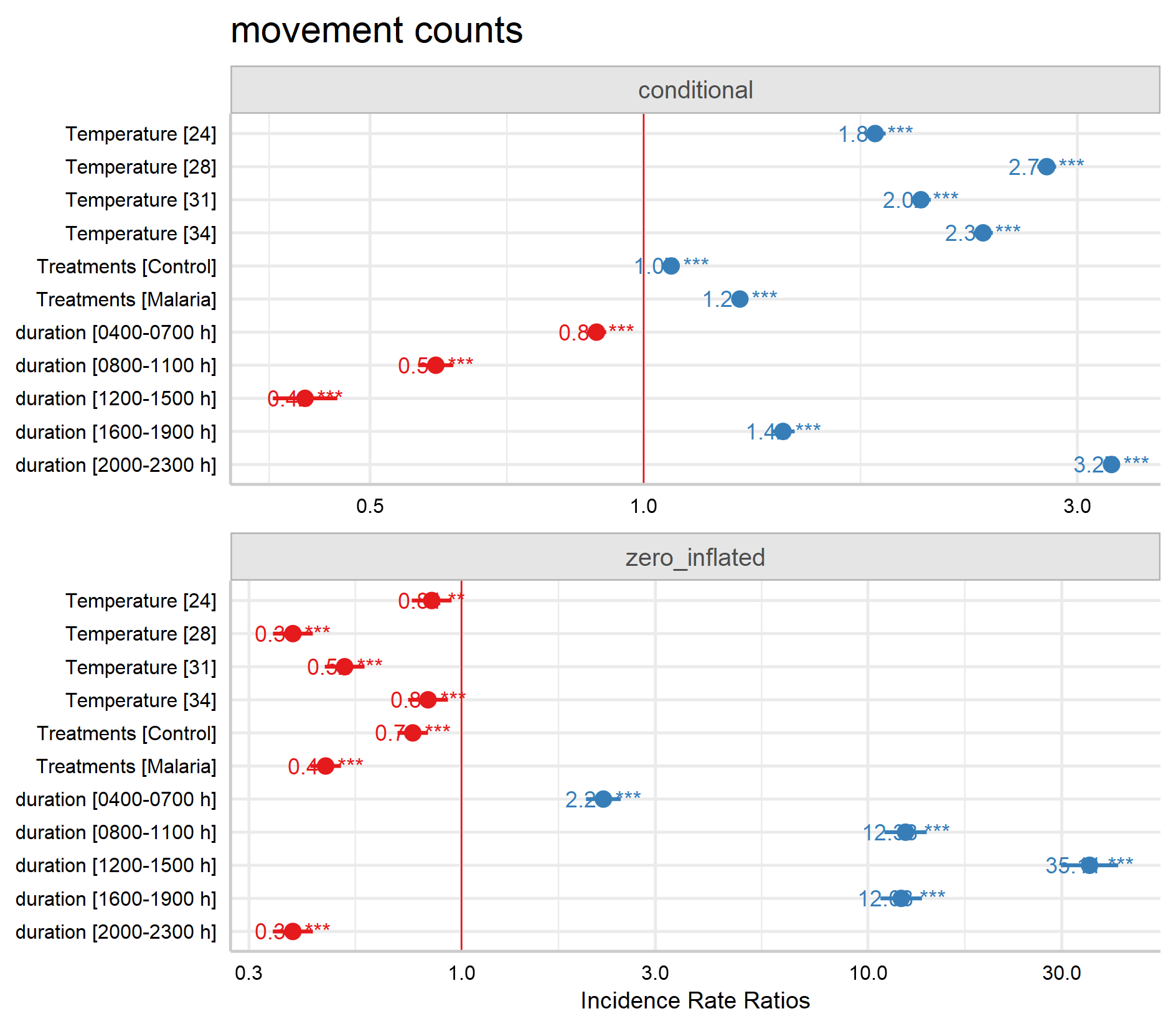

Supplement: S12 Fig — The model accounts for the excess zeroes in the data using a two-parts model, a logit for the probability of zeroes and a Poisson GLM for the counts. We use the same systemic functional model, with treatment, temperature and duration as the factors for both the probability of excess zeroes and the Poisson distributed counts. The X-axis represents the incident rate ratios (IRRs) while the Y-axis represents weeks, treatment and the 3-hour categories. The conditional figure is a representation of active period or movement counts while the zero-inflated figure is a representation of resting or period of inactivity. (TIF) [file ppat.1013139.s012.tif]
