# Supplementary material for: Regulation of diel locomotor activity and retinal responses of Anopheles stephensi by ingested histamine and serotonin is temperature- and infection-dependent
Source: PLoS Pathog. 2025 Apr 28;21(4):e1013139. doi: 10.1371/journal.ppat.1013139 (PMC12058162; doi:10.1371/journal.ppat.1013139)
Supplement: S1 Table — (DOCX) [file ppat.1013139.s013.docx]

**S1 Table.** Simple Survival Analysis (Kaplan-Meier) of uninfected *A. stephensi* provisioned weekly with malaria-associated biogenic amine treatment [10nM histamine (H) + 0.15μM 5-HT], healthy-associated treatment [1nM H + 1.5μM 5-HT] or water as a control in soaked cotton balls.

|  | **Comparison** | **Log-rank (Mantel-Cox)** | **Significant*** | **Gehan-Breslow-Wilcoxon** | **Significant** |
| --- | --- | --- | --- | --- | --- |
| **Rep 1** | Control vs Healthy | 1.209 | N | 1.209 | N |
|  | Control vs Malaria | 9.199 | Y | 5.091 | Y |
|  | Healthy vs Malaria | 4.684 | Y | 1.642 | N |
| **Rep 2** | Control vs Healthy | 8.314 | Y | 7.162 | Y |
|  | Control vs Malaria | 8.885 | Y | 6.588 | Y |
|  | Healthy vs Malaria | 0.00147 | N | 0.04496 | N |
| **Rep 3** | Control vs Healthy | 4.297 | Y | 0.1211 | N |
|  | Control vs Malaria | 0.4365 | N | 0.6812 | N |
|  | Healthy vs Malaria | 2.160 | N | 1.854 | N |
| **Rep 4** | Control vs Healthy | 1.440 | N | 3.606 | N |
|  | Control vs Malaria | 0.5306 | N | 2.075 | N |
|  | Healthy vs Malaria | 0.2396 | N | 0.3036 | N |

*Y = yes, N = no.
