# Supplementary material for: Regulation of diel locomotor activity and retinal responses of Anopheles stephensi by ingested histamine and serotonin is temperature- and infection-dependent
Source: PLoS Pathog. 2025 Apr 28;21(4):e1013139. doi: 10.1371/journal.ppat.1013139 (PMC12058162; doi:10.1371/journal.ppat.1013139)
Supplement: S2 Table — (DOCX) [file ppat.1013139.s014.docx]

**S2 Table.** Pairwise comparison (Tukey HSD) of the effect of provisioned malaria-associated biogenic amine treatment (10nM H + 0.15μM 5-HT), healthy-associated treatment (1nM H + 1.5μM 5-HT), or water (control) on diel locomotor activity across lifespan.

| Comparison of treatment groups across lifespan | | | |
| --- | --- | --- | --- |
| Treatments | **t Ratio** | **Prob>\|t\|** | **Group with higher activity** |
| Healthy vs Malaria | -6.63 | <.0001* | Malaria |
| Healthy vs Control | 2.04 | 0.1029 | Similar |
| Malaria vs Control | 8.67 | <.0001* | Malaria |
| Comparison of treatment groups at specific time periods | | | |
| 0000-0300 h | **t Ratio** | **Prob>\|t\|** | **Group with higher activity** |
| Healthy vs Malaria | -1.88 | 0.1448 | Similar |
| Healthy vs Control | -0.38 | 0.9246 | Similar |
| Malaria vs Control | 1.47 | 0.3077 | Similar |
| 0400-0700 h |  |  |  |
| Healthy vs Malaria | 1.03 | 0.5591 | Similar |
| Healthy vs Control | 2.4 | 0.043* | Healthy |
| Malaria vs Control | 1.47 | 0.3058 | Similar |
| 0800-1100 h |  |  |  |
| Healthy vs Malaria | -0.74 | 0.7413 | Similar |
| Healthy vs Control | -2.29 | 0.0578 | Similar |
| Malaria vs Control | -1.57 | 0.2596 | Similar |
| 1200-1500 h |  |  |  |
| Healthy vs Malaria | -1.73 | 0.1927 | Similar |
| Healthy vs Control | -3.44 | 0.0017* | Control |
| Malaria vs Control | -1.90 | 0.1381 | Similar |
| 1600-1900 h |  |  |  |
| Healthy vs Malaria | -3.56 | 0.0011* | Malaria |
| Healthy vs Control | 2.3 | 0.0565 | Similar |
| Malaria vs Control | 6.51 | <.0001* | Malaria |
| 2000-2300 h |  |  |  |
| Healthy vs Malaria | -2.96 | 0.0088* | Malaria |
| Heathy vs Control | 1.79 | 0.1728 | Similar |
| Malaria vs Control | 4.56 | <.0001* | Malaria |

P values ≤ 0.05 were considered significant and denoted with asterisk (*)
