# Supplementary material for: Regulation of diel locomotor activity and retinal responses of Anopheles stephensi by ingested histamine and serotonin is temperature- and infection-dependent
Source: PLoS Pathog. 2025 Apr 28;21(4):e1013139. doi: 10.1371/journal.ppat.1013139 (PMC12058162; doi:10.1371/journal.ppat.1013139)
Supplement: S3 Table — Treatments included malaria-associated biogenic amine treatment (10nM H + 0.15 μM 5-HT), healthy-associated treatment (1nM H + 1.5 μM 5-HT), or water (control). (DOCX) [file ppat.1013139.s015.docx]

**S3 Table.** Summary table for computed p-values using the Chi-square test in comparison of the number of active periods between treatment groups under light and dark duration. Treatments included malaria-associated biogenic amine treatment (10nM H + 0.15μM 5-HT), healthy-associated treatment (1nM H + 1.5μM 5-HT), or water (control).

|  |  |  |  |  |  | **Light** |  |  |  |  |
| --- | --- | --- | --- | --- | --- | --- | --- | --- | --- | --- |
| Week 1 | | | Week 2 | | Week 3 | | Week 4 | | Week 5 | |
| Treatments | **Control** | **Healthy** | **Control** | **Healthy** | **Control** | **Healthy** | **Control** | **Healthy** | **Control** | **Healthy** |
| Control |  | 0.7808 |  | 0.0953 |  | 0.0298* |  | 0.0720 |  | 0.0002* |
| Malaria | 0.614 | 0.4824 | 0.1485 | 0.0019* | 0.0242* | 0.0912 | 0.2377 | 0.0031* | 0.1218 | <0.0001* |
|  |  |  |  |  |  | **Dark** |  |  |  |  |
| Control |  | <0.0001* |  | <0.0001* |  | 0.0434* |  | 0.0039* |  | <0.0001* |
| Malaria | 0.1547 | 0.0005* | <0.0001* | <0.0001* | 0.0063* | <0.0001* | <0.0001* | <0.0001* | 0.0006* | <0.0001* |

P values ≤ 0.05 were considered significant and denoted with asterisk (*)
