# Supplementary material for: Regulation of diel locomotor activity and retinal responses of Anopheles stephensi by ingested histamine and serotonin is temperature- and infection-dependent
Source: PLoS Pathog. 2025 Apr 28;21(4):e1013139. doi: 10.1371/journal.ppat.1013139 (PMC12058162; doi:10.1371/journal.ppat.1013139)
Supplement: S4 Table — (DOCX) [file ppat.1013139.s016.docx]

**S4 Table.** Pairwise comparison (Tukey HSD) of the effect of provisioned malaria-associated biogenic amine treatment (10nM H + 0.15μM 5-HT), healthy-associated treatment (1nM H + 1.5μM 5-HT), or water (control) on diel locomotor activity over period across lifespan.

| **Week 1** | | | |
| --- | --- | --- | --- |
| Treatments | **t Ratio** | **Prob>\|t\|** | **Higher activity** |
| 0000-0300 |  |  |  |
| Healthy vs Malaria | -2.64 | 0.0228* | Malaria |
| Healthy vs Control | -1.91 | 0.1362 | Similar |
| Malaria vs Control | 0.73 | 0.7463 | Similar |
| 0400-0700 |  |  |  |
| Healthy vs Malaria | -1.62 | 0.2362 | Similar |
| Healthy vs Control | -0.12 | 0.9918 | Similar |
| Malaria vs Control | 1.44 | 0.3211 | Similar |
| 0800-1100 |  |  |  |
| Healthy vs Malaria | -1.7 | 0.2073 | Similar |
| Healthy vs Control | -2.07 | 0.0972 | Similar |
| Malaria vs Control | -0.5 | 0.8708 | Similar |
| 1200-1500 |  |  |  |
| Healthy vs Malaria | -0.71 | 0.758 | Similar |
| Healthy vs Control | -3.35 | 0.0025* | Healthy |
| Malaria vs Control | -2.69 | 0.0198* | Malaria |
| 1600-1900 |  |  |  |
| Healthy vs Malaria | -2.44 | 0.0394* | Malaria |
| Healthy vs Control | 1.7 | 0.2073 | Similar |
| Malaria vs Control | 4.12 | 0.0001* | Malaria |
| 2000-2300 |  |  |  |
| Healthy vs Malaria | -2.43 | 0.0404* | Malaria |
| Healthy vs Control | 0.01 | 1 | Similar |
| Malaria vs Control | 2.21 | 0.0707 | Similar |
| **Week 2** | | | |
| 0000-0300 |  |  |  |
| Healthy vs Malaria | -0.2 | 0.978 | Similar |
| Healthy vs Control | 0.1 | 0.994 | Similar |
| Malaria vs Control | 0.31 | 0.9483 | Similar |
| 0400-0700 |  |  |  |
| Healthy vs Malaria | 0.64 | 0.7956 | Similar |
| Healthy vs Control | 0.99 | 0.5844 | Similar |
| Malaria vs Control | 0.39 | 0.9178 | Similar |
| 0800-1100 |  |  |  |
| Healthy vs Malaria | -2.03 | 0.1049 | Similar |
| Healthy vs Control | -2.3 | 0.0564 | Similar |
| Malaria vs Control | -0.25 | 0.9657 | Similar |
| 1200-1500 |  |  |  |
| Healthy vs Malaria | -1.67 | 0.2181 | Similar |
| Healthy vs Control | 2.26 | 0.062* | Healthy |
| Malaria vs Control | 3.54 | 0.0012* | Malaria |
| 1600-1900 |  |  |  |
| Healthy vs Malaria | -2.19 | 0.0735 | Similar |
| Healthy vs Control | 0.82 | 0.6895 | Similar |
| Malaria vs Control | 3.18 | 0.0043* | Malaria |
| 2000-2300 |  |  |  |
| Healthy vs Malaria | -1.96 | 0.1221 | Similar |
| Healthy vs Control | -0.09 | 0.9959 | Similar |
| Malaria vs Control | 1.85 | 0.1543 | Similar |
| **Week 3** | | | |
| 0000-0300 |  |  |  |
| Healthy vs Malaria | -0.29 | 0.954 | Similar |
| Healthy vs Control | 1.5 | 0.2923 | Similar |
| Malaria vs Control | 1.84 | 0.1562 | Similar |
| 0400-0700 |  |  |  |
| Healthy vs Malaria | 2.35 | 0.0497* | Healthy |
| Healthy vs Control | 2.76 | 0.0164* | Healthy |
| Malaria vs Control | 0.53 | 0.8551 | Similar |
| 0800-1100 |  |  |  |
| Healthy vs Malaria | -1.61 | 0.2433 | Similar |
| Healthy vs Control | -3.06 | 0.0065* | Control |
| Malaria vs Control | -1.56 | 0.2623 | Similar |
| 1200-1500 |  |  |  |
| Healthy vs Malaria | 1.04 | 0.5517 | Similar |
| Healthy vs Control | -1.21 | 0.4495 | Similar |
| Malaria vs Control | -2.18 | 0.0753 | Similar |
| 1600-1900 |  |  |  |
| Healthy vs Malaria | -4.93 | <.0001* | Malaria |
| Healthy vs Control | -0.51 | 0.8661 | Similar |
| Malaria vs Control | 5.06 | <.0001* | Malaria |
| 2000-2300 |  |  |  |
| Healthy vs Malaria | -0.79 | 0.7106 | Similar |
| Healthy vs Control | 2.81 | 0.014* | Healthy |
| Malaria vs Control | 3.51 | 0.0014* | Malaria |
| **Week 4** | | | |
| 0000-0300 |  |  |  |
| Healthy vs Malaria | -1.2 | 0.4518 | Similar |
| Healthy vs Control | -0.66 | 0.7858 | Similar |
| Malaria vs Control | 0.7 | 0.764 | Similar |
| 0400-0700 |  |  |  |
| Healthy vs Malaria | -3.78 | 0.0005* | Malaria |
| Healthy vs Control | -2.95 | 0.0093* | Control |
| Malaria vs Control | 1.28 | 0.4064 | Similar |
| 0800-1100 |  |  |  |
| Healthy vs Malaria | 0 | 1 | Similar |
| Healthy vs Control | -1.76 | 0.1854 | Similar |
| Malaria vs Control | -1.76 | 0.1854 | Similar |
| 1200-1500 |  |  |  |
| Healthy vs Malaria | -1.63 | 0.2352 | Similar |
| Healthy vs Control | 0.11 | 0.9935 | Similar |
| Malaria vs Control | 1.23 | 0.4379 | Similar |
| 1600-1900 |  |  |  |
| Healthy vs Malaria | -2.31 | 0.0553 | Similar |
| Healthy vs Control | 0.03 | 0.9995 | Similar |
| Malaria vs Control | 2.73 | 0.018* | Malaria |
| 2000-2300 |  |  |  |
| Healthy vs Malaria | -3.4 | 0.0021* | Malaria |
| Healthy vs Control | -0.22 | 0.9735 | Similar |
| Malaria vs Control | 3.66 | 0.0008* | Malaria |
| **Week 5** | | | |
| 0000-0300 |  |  |  |
| Healthy vs Malaria | -0.02 | 0.9997 | Similar |
| Healthy vs Control | 2.65 | 0.0221* | Healthy |
| Malaria vs Control | 2.52 | 0.0321* | Malaria |
| 0400-0700 |  |  |  |
| Healthy vs Malaria | 1.94 | 0.1283 | Similar |
| Healthy vs Control | 1.17 | 0.4725 | Similar |
| Malaria vs Control | -0.57 | 0.8375 | Similar |
| 0800-1100 |  |  |  |
| Healthy vs Malaria | 0.07 | 0.997 | Similar |
| Healthy vs Control | 1.76 | 0.1846 | Similar |
| Malaria vs Control | 1.69 | 0.2102 | Similar |
| 1200-1500 |  |  |  |
| Healthy vs Malaria | 0.24 | 0.9687 | Similar |
| Healthy vs Control | -0.93 | 0.6213 | Similar |
| Malaria vs Control | -1.33 | 0.3804 | Similar |
| 1600-1900 |  |  |  |
| Healthy vs Malaria | -1.77 | 0.1814 | Similar |
| Healthy vs Control | -2.17 | 0.0775 | Similar |
| Malaria vs Control | -0.73 | 0.7436 | Similar |
| 2000-2300 |  |  |  |
| Healthy vs Malaria | 1.84 | 0.1584 | Similar |
| Healthy vs Control | 1.85 | 0.1552 | Similar |
| Malaria vs Control | 0.26 | 0.9634 | Similar |

P values ≤ 0.05 were considered significant and denoted with asterisk (*)
