# Supplementary material for: Regulation of diel locomotor activity and retinal responses of Anopheles stephensi by ingested histamine and serotonin is temperature- and infection-dependent
Source: PLoS Pathog. 2025 Apr 28;21(4):e1013139. doi: 10.1371/journal.ppat.1013139 (PMC12058162; doi:10.1371/journal.ppat.1013139)
Supplement: S5 Table — (DOCX) [file ppat.1013139.s017.docx]

**S5 Table.** Output of conditional and zero inflated model results from movement counts data modeled as a function of treatment levels, week, and time discretized into 3-hourly categories.

| **Conditional** | | | | **Zero-Inflated** | | | |
| --- | --- | --- | --- | --- | --- | --- | --- |
| **Characteristic** | **exp(Beta)** | **95% CI** | **p-value** | **Characteristic** | **exp(Beta)** | **95% CI** | **p-value** |
| Week |  |  |  | Week |  |  |  |
| Week 1 | — | — |  | Week 1 | — | — |  |
| Week 2 | 0.88 | 0.86, 0.90 | <0.001 | Week 2 | 0.91 | 0.82, 1.01 | 0.078 |
| Week 3 | 0.76 | 0.74, 0.78 | <0.001 | Week 3 | 1.45 | 1.31, 1.61 | <0.001 |
| Week 4 | 0.52 | 0.50, 0.54 | <0.001 | Week 4 | 2.94 | 2.63, 3.28 | <0.001 |
| Week 5 | 0.37 | 0.35, 0.39 | <0.001 | Week 5 | 4.17 | 3.70, 4.69 | <0.001 |
| Treatments |  |  |  | Treatments |  |  |  |
| Healthy | — | — |  | Healthy | — | — |  |
| Malaria | 1.17 | 1.15, 1.20 | <0.001 | Malaria | 0.75 | 0.69, 0.82 | <0.001 |
| Control | 0.95 | 0.93, 0.98 | <0.001 | Control | 1.05 | 0.96, 1.14 | 0.3 |
| Duration |  |  |  | Duration |  |  |  |
| 0000-0300 h | — | — |  | 0000-0300 h | — | — |  |
| 0400-0700 h | 0.75 | 0.73, 0.78 | <0.001 | 0400-0700 h | 2.40 | 2.17, 2.66 | <0.001 |
| 0800-1100 h | 0.47 | 0.42, 0.54 | <0.001 | 0800-1100 h | 33.1 | 26.9, 40.8 | <0.001 |
| 1200-1500 h | 0.47 | 0.42, 0.53 | <0.001 | 1200-1500 h | 30.4 | 24.9, 37.2 | <0.001 |
| 1600-1900 h | 1.60 | 1.56, 1.65 | <0.001 | 1600-1900 h | 2.51 | 2.27, 2.79 | <0.001 |
| 2000-2300 h | 1.67 | 1.63, 1.71 | <0.001 | 2000-2300 h | 1.02 | 0.92, 1.12 | 0.8 |
