# Supplementary material for: Regulation of diel locomotor activity and retinal responses of Anopheles stephensi by ingested histamine and serotonin is temperature- and infection-dependent
Source: PLoS Pathog. 2025 Apr 28;21(4):e1013139. doi: 10.1371/journal.ppat.1013139 (PMC12058162; doi:10.1371/journal.ppat.1013139)
Supplement: S6 Table — (DOCX) [file ppat.1013139.s018.docx]

**S6 Table.** Generalized linear mixed model results for ERG responses of uninfected *A. stephensi*, assessing the effects of biogenic amine treatments (“Treat”: healthy = 1nM H + 1.5μM 5-HT, malaria = 10nM H + 0.15μM 5-HT, control = water), time of experiments (“Time”: am = 0800-1100 h, pm = 2000-2300 h), and wavelengths (“Wvlength”: light stimuli from 320 to 700 nm in 10 nm increments).

| **Summary** | | | | | | |
| --- | --- | --- | --- | --- | --- | --- |
| Model: | MixedLM | | | | | |
| No. observations: | 1836 | | | | | |
| No. groups: | 52 | | | | | |
| Min. group size: | 13 | | | | | |
| Max. group size: | 50 | | | | | |
| Mean group size: | 35.3 | | | | | |
| Dependent variable: | Amplitude | | | | | |
| Method: | REML | | | | | |
| Scale: | 1.1124 | | | | | |
| Log-Likelihood: | -2841.0008 | | | | | |
| Converged: | Yes | | | | | |
| **Statistics** | | | | | | |
|  | Coef. | Std.Err. | z | P>\|z\| | [0.025 | 0.975] |
| Intercept | 3.166 | 0.74 | 4.279 | 0.000 | 1.716 | 4.617 |
| C(Treat)[T.healthy] | 0.860 | 0.977 | 0.881 | 0.378 | -1.054 | 2.775 |
| C(Treat)[T.malaria] | 1.545 | 1.024 | 1.508 | 0.132 | -0.463 | 3.553 |
| C(Time)[T.pm] | 1.754 | 1.096 | 1.601 | 0.109 | -0.393 | 3.902 |
| C(Wvlength)[T.330] | 0.225 | 0.221 | 1.016 | 0.31 | -0.209 | 0.659 |
| C(Wvlength)[T.340] | -0.086 | 0.220 | -0.392 | 0.695 | -0.518 | 0.345 |
| C(Wvlength)[T.350] | 0.402 | 0.219 | 1.837 | 0.066 | -0.027 | 0.832 |
| C(Wvlength)[T.360] | -0.044 | 0.218 | -0.202 | 0.84 | -0.471 | 0.383 |
| C(Wvlength)[T.370] | -0.237 | 0.222 | -1.072 | 0.284 | -0.672 | 0.197 |
| C(Wvlength)[T.380] | -0.580 | 0.222 | -2.619 | 0.009 | -1.014 | -0.146 |
| C(Wvlength)[T.390] | -0.732 | 0.217 | -3.378 | 0.001 | -1.156 | -0.307 |
| C(Wvlength)[T.400] | -0.996 | 0.217 | -4.597 | 0 | -1.421 | -0.572 |
| C(Wvlength)[T.410] | -0.949 | 0.218 | -4.358 | 0 | -1.376 | -0.522 |
| C(Wvlength)[T.420] | -0.696 | 0.217 | -3.212 | 0.001 | -1.12 | -0.271 |
| C(Wvlength)[T.430] | -0.593 | 0.220 | -2.693 | 0.007 | -1.025 | -0.161 |
| C(Wvlength)[T.440] | -0.487 | 0.217 | -2.245 | 0.025 | -0.911 | -0.062 |
| C(Wvlength)[T.450] | -0.413 | 0.223 | -1.851 | 0.064 | -0.849 | 0.024 |
| C(Wvlength)[T.460] | -0.229 | 0.220 | -1.038 | 0.299 | -0.660 | 0.203 |
| C(Wvlength)[T.470] | -0.106 | 0.219 | -0.485 | 0.628 | -0.536 | 0.323 |
| C(Wvlength)[T.480] | 0.102 | 0.221 | 0.461 | 0.644 | -0.332 | 0.536 |
| C(Wvlength)[T.490] | -0.035 | 0.217 | -0.162 | 0.871 | -0.460 | 0.39 |
| C(Wvlength)[T.500] | 0.130 | 0.218 | 0.598 | 0.55 | -0.297 | 0.557 |
| C(Wvlength)[T.510] | 0.127 | 0.219 | 0.578 | 0.563 | -0.303 | 0.556 |
| C(Wvlength)[T.520] | 0.151 | 0.218 | 0.694 | 0.488 | -0.276 | 0.578 |
| C(Wvlength)[T.530] | 0.193 | 0.217 | 0.89 | 0.374 | -0.232 | 0.618 |
| C(Wvlength)[T.540] | 0.168 | 0.216 | 0.777 | 0.437 | -0.255 | 0.59 |
| C(Wvlength)[T.550] | 0.292 | 0.219 | 1.333 | 0.183 | -0.137 | 0.72 |
| C(Wvlength)[T.560] | -0.025 | 0.218 | -0.116 | 0.907 | -0.452 | 0.401 |
| C(Wvlength)[T.570] | 0.009 | 0.218 | 0.039 | 0.969 | -0.419 | 0.436 |
| C(Wvlength)[T.580] | -0.307 | 0.220 | -1.392 | 0.164 | -0.738 | 0.125 |
| C(Wvlength)[T.590] | -0.671 | 0.216 | -3.112 | 0.002 | -1.093 | -0.248 |
| C(Wvlength)[T.600] | -0.870 | 0.217 | -4.014 | 0 | -1.294 | -0.445 |
| C(Wvlength)[T.610] | -1.452 | 0.216 | -6.732 | 0 | -1.875 | -1.029 |
| C(Wvlength)[T.620] | -1.817 | 0.218 | -8.339 | 0 | -2.244 | -1.39 |
| C(Wvlength)[T.630] | -2.280 | 0.217 | -10.514 | 0 | -2.705 | -1.855 |
| C(Wvlength)[T.640] | -2.705 | 0.214 | -12.669 | 0 | -3.124 | -2.287 |
| C(Wvlength)[T.650] | -3.067 | 0.212 | -14.492 | 0 | -3.482 | -2.652 |
| C(Wvlength)[T.660] | -3.250 | 0.217 | -15.003 | 0 | -3.675 | -2.826 |
| C(Wvlength)[T.670] | -3.445 | 0.218 | -15.811 | 0 | -3.872 | -3.018 |
| C(Wvlength)[T.680] | -3.595 | 0.214 | -16.831 | 0 | -4.014 | -3.176 |
| C(Wvlength)[T.690] | -3.721 | 0.215 | -17.268 | 0 | -4.143 | -3.299 |
| C(Wvlength)[T.700] | -3.654 | 0.218 | -16.77 | 0 | -4.081 | -3.227 |
| C(Treat)[T.healthy]:C(Time)[T.pm] | -1.813 | 1.490 | -1.216 | 0.224 | -4.734 | 1.109 |
| C(Treat)[T.malaria]:C(Time)[T.pm] | -3.158 | 1.522 | -2.075 | 0.038 | -6.14 | -0.175 |
| Group Var | 4.691 | 0.946 |  |  |  |  |
