# Supplementary material for: Regulation of diel locomotor activity and retinal responses of Anopheles stephensi by ingested histamine and serotonin is temperature- and infection-dependent
Source: PLoS Pathog. 2025 Apr 28;21(4):e1013139. doi: 10.1371/journal.ppat.1013139 (PMC12058162; doi:10.1371/journal.ppat.1013139)
Supplement: S7 Table — Treatments included malaria-associated biogenic amine treatment (10nM H + 0.15 μM 5-HT), healthy-associated treatment (1nM H + 1.5 μM 5-HT), or water (control). (DOCX) [file ppat.1013139.s019.docx]

**S7 Table.** Pairwise comparisons (Tukey HSD) of diel locomotor activity patterns between time periods by treatment group and temperature. Treatments included malaria-associated biogenic amine treatment (10nM H + 0.15μM 5-HT), healthy-associated treatment (1nM H + 1.5μM 5-HT), or water (control).

| 21°C | | | | | | | | | | |
| --- | --- | --- | --- | --- | --- | --- | --- | --- | --- | --- |
|  | **Control** | | | **Healthy** | | | **Malaria** | | | |
| **Period (24h)** | **t Ratio** | **Prob>\|t\|** | **Higher activity** | **t Ratio** | **Prob>\|t\|** | **Higher activity** | **t Ratio** | **Prob>\|t\|** | **Higher activity** |  |
| 0000-0300 vs 0400-0700 | 5.02 | <.0001* | 0000-0300 | 0.66 | 0.9866 | Similar | 8.71 | <.0001* | 0000-0300 |  |
| 0000-0300 vs 1600-1900 | 5.29 | <.0001* | 0000-0300 | 4.23 | 0.0004* | 0000-0300 | 11.64 | <.0001* | 0000-0300 |  |
| 0000-0300 vs 2000-2300 | -4.76 | <.0001* | 2000-2300 | -4.39 | 0.0002* | 2000-2300 | -6.36 | <.0001* | 2000-2300 |  |
| 0400-0700 vs 1600-1900 | 0.45 | 0.9977 | Similar | 3.65 | 0.0038* | 0400-0700 | 3.33 | 0.0114* | 0400-0700 |  |
| 0400-0700 vs 2000-2300 | -8.78 | <.0001* | 2000-2300 | -5.05 | <.0001* | 2000-2300 | -14.72 | <.0001* | 2000-2300 |  |
| 1600-1900 vs 2000-2300 | -8.75 | <.0001* | 2000-2300 | -7.75 | <.0001* | 2000-2300 | -17.26 | <.0001* | 2000-2300 |  |
| 24°C | | | | | | | | | | |
| 0000-0300 vs 0400-0700 | -0.13 | 1 | Similar | -0.04 | 1 | Similar | 0.99 | 0.9208 | Similar |  |
| 0000-0300 vs 1600-1900 | 4.74 | <.0001* | 0000-0300 | 9.85 | <.0001* | 0000-0300 | -1.24 | 0.8146 | Similar |  |
| 0000-0300 vs 2000-2300 | -2.79 | 0.0593 | Similar | -3.75 | 0.0025* | 2000-2300 | -5.09 | <.0001* | 2000-2300 |  |
| 0400-0700 vs 1600-1900 | 4.78 | <.0001* | 0400-0700 | 9.86 | <.0001* | 0400-0700 | -1.99 | 0.3475 | Similar |  |
| 0400-0700 vs 2000-2300 | -2.69 | 0.0777 | Similar | -3.6 | 0.0044* | 2000-2300 | -5.85 | <.0001* | 2000-2300 |  |
| 1600-1900 vs 2000-2300 | -6.87 | <.0001* | 2000-2300 | -12.46 | <.0001* | 2000-2300 | -2.3 | 0.1946 | Similar |  |
| 28°C | | | | | | | | | | |
| 0000-0300 vs 0400-0700 | 5.46 | <.0001* | 0000-0300 | 4.06 | 0.0007* | 0000-0300 | 2.84 | 0.0515 | Similar |  |
| 0000-0300 vs 1600-1900 | -9.32 | <.0001* | 1600-1900 | -12.51 | <.0001* | 1600-1900 | -13.11 | <.0001* | 1600-1900 |  |
| 0000-0300 vs 2000-2300 | -8.1 | <.0001* | 2000-2300 | -9.59 | <.0001* | 2000-2300 | -9.4 | <.0001* | 2000-2300 |  |
| 0400-0700 vs 1600-1900 | -14.17 | <.0001* | 1600-1900 | -15.17 | <.0001* | 1600-1900 | -15.1 | <.0001* | 1600-1900 |  |
| 0400-0700 vs 2000-2300 | -12.47 | <.0001* | 2000-2300 | -12.82 | <.0001* | 2000-2300 | -11.79 | <.0001* | 2000-2300 |  |
| 1600-1900 vs 2000-2300 | 1.83 | 0.4476 | Similar | 5.44 | <.0001* | 1600-1900 | 5.68 | <.0001* | 1600-1900 |  |
| 31°C | | | | | | | | | | |
| 0000-0300 vs 0400-0700 | 2.45 | 0.1393 | Similar | 4.19 | 0.0004* | 0000-0300 | 3.89 | 0.0015* | 0000-0300 |  |
| 0000-0300 vs 1600-1900 | -11.98 | <.0001* | 1600-1900 | -6.52 | <.0001* | 1600-1900 | -16.16 | <.0001* | 1600-1900 |  |
| 0000-0300 vs 2000-2300 | -8.32 | <.0001* | 2000-2300 | -4.77 | <.0001* | 2000-2300 | -6.32 | <.0001* | 2000-2300 |  |
| 0400-0700 vs 1600-1900 | -13.66 | <.0001* | 1600-1900 | -9.8 | <.0001* | 1600-1900 | -19.6 | <.0001* | 1600-1900 |  |
| 0400-0700 vs 2000-2300 | -10.1 | <.0001* | 2000-2300 | -8.1 | <.0001* | 2000-2300 | -10.14 | <.0001* | 2000-2300 |  |
| 1600-1900 vs 2000-2300 | 4.13 | 0.0006* | Similar | 2.37 | 0.168 | Similar | 10.44 | <.0001* | 1600-1900 |  |
| 34°C | | | | | | | | | | |
| 0000-0300 vs 0400-0700 | 6.88 | <.0001* | 0000-0300 | 3.91 | 0.0014* | 0000-0300 | 4.48 | 0.0001* | 0000-0300 |  |
| 0000-0300 vs 1600-1900 | -21.04 | <.0001* | 1600-1900 | -20.26 | <.0001* | 1600-1900 | -17.95 | <.0001* | 1600-1900 |  |
| 0000-0300 vs 2000-2300 | -11.81 | <.0001* | 2000-2300 | -14.86 | <.0001* | 2000-2300 | -12.38 | <.0001* | 2000-2300 |  |
| 0400-0700 vs 1600-1900 | -23.95 | <.0001* | 1600-1900 | -21.99 | <.0001* | 1600-1900 | -20.46 | <.0001* | 1600-1900 |  |
| 0400-0700 vs 2000-2300 | -16.58 | <.0001* | 2000-2300 | -17.14 | <.0001* | 2000-2300 | -15.51 | <.0001* | 2000-2300 |  |
| 1600-1900 vs 2000-2300 | 14 | <.0001* | 1600-1900 | 9.73 | <.0001* | 1600-1900 | 8.64 | <.0001* | 1600-1900 |  |

P values ≤ 0.05 were considered significant and denoted with asterisk (*)
