# Supplementary material for: Regulation of diel locomotor activity and retinal responses of Anopheles stephensi by ingested histamine and serotonin is temperature- and infection-dependent
Source: PLoS Pathog. 2025 Apr 28;21(4):e1013139. doi: 10.1371/journal.ppat.1013139 (PMC12058162; doi:10.1371/journal.ppat.1013139)
Supplement: S8 Table — Treatments included malaria-associated biogenic amine treatment (10nM H + 0.15 μM 5-HT), healthy-associated treatment (1nM H + 1.5 μM 5-HT), or water (control). (DOCX) [file ppat.1013139.s020.docx]

**S8 Table.** Pairwise comparisons (Tukey HSD) of diel locomotor activity patterns between treatment group by period and temperature. Treatments included malaria-associated biogenic amine treatment (10nM H + 0.15μM 5-HT), healthy-associated treatment (1nM H + 1.5μM 5-HT), or water (control).

| 21°C | | | |
| --- | --- | --- | --- |
| Treatments | **t Ratio** | **Prob>\|t\|** | **Higher activity** |
| 0000-0300  Healthy vs Malaria |  |  |  |
|  | -2.19 | 0.0736 | Similar |
| Heathy vs Control | -2.25 | 0.0641 | Similar |
| Malaria vs Control | -0.07 | 0.9976 | Similar |
| 0400-0700 |  |  |  |
| Healthy vs Malaria | 3.04 | 0.007* | Healthy |
| Heathy vs Control | 1.19 | 0.4591 | Similar |
| Malaria vs Control | -1.89 | 0.1425 | Similar |
| 0800-1100 |  |  |  |
| Healthy vs Malaria | -1.21 | 0.4458 | Similar |
| Heathy vs Control | 0.24 | 0.9683 | Similar |
| Malaria vs Control | 1.33 | 0.3815 | Similar |
| 1200-1500 |  |  |  |
| Healthy vs Malaria | 0.81 | 0.6952 | Similar |
| Heathy vs Control | 0.25 | 0.9654 | Similar |
| Malaria vs Control | -0.63 | 0.8059 | Similar |
| 1600-1900 |  |  |  |
| Healthy vs Malaria | 1.26 | 0.4193 | Similar |
| Heathy vs Control | -1.01 | 0.5715 | Similar |
| Malaria vs Control | -2.26 | 0.063 | Similar |
| 2000-2300 |  |  |  |
| Healthy vs Malaria | -3.64 | 0.0009* | Malaria |
| Heathy vs Control | -1.89 | 0.1416 | Similar |
| Malaria vs Control | 1.93 | 0.1306 | Similar |
| 24°C | | | |
| 000-0300 |  |  |  |
| Healthy vs Malaria | -1.58 | 0.2533 | Similar |
| Heathy vs Control | -1.04 | 0.5532 | Similar |
| Malaria vs Control | 0.48 | 0.883 | Similar |
| 0400-0700 |  |  |  |
| Healthy vs Malaria | -0.26 | 0.9632 | Similar |
| Heathy vs Control | -0.72 | 0.7488 | Similar |
| Malaria vs Control | -0.49 | 0.8782 | Similar |
| 0800-1100 |  |  |  |
| Healthy vs Malaria | -2.86 | 0.0122* | Malaria |
| Heathy vs Control | -4.29 | <.0001* | Control |
| Malaria vs Control | -1.5 | 0.291 | Similar |
| 1200-1500 |  |  |  |
| Healthy vs Malaria | 0.61 | 0.8159 | Similar |
| Heathy vs Control | -0.13 | 0.9903 | Similar |
| Malaria vs Control | -0.98 | 0.5867 | Similar |
| 1600-1900 |  |  |  |
| Healthy vs Malaria | -4.47 | <.0001* | Malaria |
| Heathy vs Control | -2.2 | 0.0712* | Control |
| Malaria vs Control | 3.38 | 0.0022* | Malaria |
| 2000-2300 |  |  |  |
| Healthy vs Malaria | -1.64 | 0.2295 | Similar |
| Heathy vs Control | -0.2 | 0.979 | Similar |
| Malaria vs Control | 1.65 | 0.2256 | Similar |
| 28°C | | | |
| 0000-0300 |  |  |  |
| Healthy vs Malaria | -0.54 | 0.852 | Similar |
| Heathy vs Control | 2.17 | 0.0774 | Similar |
| Malaria vs Control | 2.64 | 0.023* | Malaria |
| 0400-0700 |  |  |  |
| Healthy vs Malaria | -1.44 | 0.3213 | Similar |
| Heathy vs Control | 2.67 | 0.0211* | Healthy |
| Malaria vs Control | 3.89 | 0.0003* | Malaria |
| 0800-1100 |  |  |  |
| Healthy vs Malaria | 0.77 | 0.724 | Similar |
| Heathy vs Control | 0.05 | 0.9985 | Similar |
| Malaria vs Control | -0.31 | 0.9495 | Similar |
| 1200-1500 |  |  |  |
| Healthy vs Malaria | 0.35 | 0.9331 | Similar |
| Heathy vs Control | -1.38 | 0.3523 | Similar |
| Malaria vs Control | -1.56 | 0.2619 | Similar |
| 1600-1900 |  |  |  |
| Healthy vs Malaria | -0.75 | 0.7317 | Similar |
| Heathy vs Control | 1.61 | 0.2413 | Similar |
| Malaria vs Control | 2.55 | 0.0293* | Malaria |
| 2000-2300 |  |  |  |
| Healthy vs Malaria | -1.05 | 0.5426 | Similar |
| Heathy vs Control | 1.31 | 0.3873 | Similar |
| Malaria vs Control | 2.36 | 0.0489* | Malaria |
| 31°C | | | |
| 0000-0300 |  |  |  |
| Healthy vs Malaria | -8.26 | <.0001* | Malaria |
| Heathy vs Control | -0.55 | 0.8459 | Similar |
| Malaria vs Control | 7.55 | <.0001* | Malaria |
| 0400-0700 |  |  |  |
| Healthy vs Malaria | -8.1 | <.0001* | Malaria |
| Heathy vs Control | -3.81 | 0.0004* | Control |
| Malaria vs Control | 5.22 | <.0001* | Malaria |
| 0800-1100 |  |  |  |
| Healthy vs Malaria | -3.88 | 0.0003* | Malaria |
| Heathy vs Control | -3.47 | 0.0016* | Control |
| Malaria vs Control | 0.54 | 0.8495 | Similar |
| 1200-1500 |  |  |  |
| Healthy vs Malaria | -1.83 | 0.1611 | Similar |
| Heathy vs Control | -3.69 | 0.0007* | Control |
| Malaria vs Control | -2.02 | 0.1078 | Similar |
| 1600-1900 |  |  |  |
| Healthy vs Malaria | -9.54 | <.0001* | Malaria |
| Heathy vs Control | -4.08 | 0.0001* | Control |
| Malaria vs Control | 6.75 | <.0001* | Malaria |
| 2000-2300 |  |  |  |
| Healthy vs Malaria | -8.26 | <.0001* | Malaria |
| Heathy vs Control | -5.62 | <.0001* | Control |
| Malaria vs Control | 3.24 | 0.0036* | Malaria |
| 34°C | | | |
| 000-0300 |  |  |  |
| Healthy vs Malaria | -2.86 | 0.012* | Malaria |
| Heathy vs Control | 0.31 | 0.9472 | Similar |
| Malaria vs Control | 2.99 | 0.0081* | Malaria |
| 0400-0700 |  |  |  |
| Healthy vs Malaria | -1.58 | 0.2528 | Similar |
| Heathy vs Control | 3.32 | 0.0027* | Healthy |
| Malaria vs Control | 4.64 | <.0001* | Malaria |
| 0800-1100 |  |  |  |
| Healthy vs Malaria | -6.34 | <.0001* | Malaria |
| Heathy vs Control | -1.88 | 0.144 | Similar |
| Malaria vs Control | 4.97 | <.0001* | Malaria |
| 1200-1500 |  |  |  |
| Healthy vs Malaria | -5.06 | <.0001* | Malaria |
| Heathy vs Control | -4.35 | <.0001* | Control |
| Malaria vs Control | 1.5 | 0.2893 | Similar |
| 1600-1900 |  |  |  |
| Healthy vs Malaria | -0.2 | 0.9788 | Similar |
| Heathy vs Control | -2.98 | 0.0085* | Control |
| Malaria vs Control | -2.92 | 0.01* | Control |
| 2000-2300 |  |  |  |
| Healthy vs Malaria | -0.8 | 0.7012 | Similar |
| Heathy vs Control | 2.22 | 0.068 | Similar |
| Malaria vs Control | 3.02 | 0.0073* | Malaria |

P values ≤ 0.05 were considered significant and denoted with asterisk (*)
