# Supplementary material for: Regulation of diel locomotor activity and retinal responses of Anopheles stephensi by ingested histamine and serotonin is temperature- and infection-dependent
Source: PLoS Pathog. 2025 Apr 28;21(4):e1013139. doi: 10.1371/journal.ppat.1013139 (PMC12058162; doi:10.1371/journal.ppat.1013139)
Supplement: S9 Table — Treatments included malaria-associated biogenic amine treatment (10nM H + 0.15 μM 5-HT), healthy-associated treatment (1nM H + 1.5 μM 5-HT), or water (control). (DOCX) [file ppat.1013139.s021.docx]

**S9 Table.** Summary table for computed p-values using the Chi-square test in comparison of the number of active periods between temperature across treatments under light and dark duration. Treatments included malaria-associated biogenic amine treatment (10nM H + 0.15μM 5-HT), healthy-associated treatment (1nM H + 1.5μM 5-HT), or water (control).

| **Light** | | | | | | |
| --- | --- | --- | --- | --- | --- | --- |
| **Temperature (°C)** | **Control** | | **Healthy** | | **Malaria** | |
|  | **P-value** | **Higher movement** | **P-value** | **Higher movement** | **P-value** | **Higher movement** |
| 21 vs 24 | 0.8661 | Similar | 0.0007* | 21°C | 0.0002* | 24°C |
| 24 vs 28 | <0.0001* | 28°C | <0.0001* | 28°C | 0.0002* | 28°C |
| 28 vs 31 | 0.6289 | Similar | <0.0001* | 28°C | 0.0003* | 31°C |
| 31 vs 34 | <0.0001* | 31°C | 0.4068 | Similar | 0.1366 | Similar |
| **Dark** | | | | | | |
| 21 vs 24 | 0.5926 | Similar | 0.0511 | Similar | <0.0001* | 24°C |
| 24 vs 28 | 0.0259* | 28°C | <0.0001* | 28°C | 0.2595 | Similar |
| 28 vs 31 | 0.0029* | 28°C | <0.0001* | 28°C | <0.0001* | 31°C |
| 31 vs 34 | 0.0003* | 31°C | <0.0001* | 34°C | <0.0001* | 31°C |

P values ≤ 0.05 considered significant by Chi-square are denoted with asterisk (*)
