# Supplementary material for: Regulation of diel locomotor activity and retinal responses of Anopheles stephensi by ingested histamine and serotonin is temperature- and infection-dependent
Source: PLoS Pathog. 2025 Apr 28;21(4):e1013139. doi: 10.1371/journal.ppat.1013139 (PMC12058162; doi:10.1371/journal.ppat.1013139)
Supplement: S10 Table — (DOCX) [file ppat.1013139.s022.docx]

**S10 Table.** Output of conditional and zero inflated model results from movement counts data modeled as a function of treatment levels, week, and time discretized into 3-hourly categories.

| **Conditional** | | | | **Zero inflated** | | | |
| --- | --- | --- | --- | --- | --- | --- | --- |
| **Characteristic** | **exp(Beta)** | **95% CI** | **p-value** | **Characteristic** | **exp(Beta)** | **95% CI** | **p-value** |
| Temperature (°C) |  |  |  | Temperature (°C) |  |  |  |
| 21 | — | — |  | 21 | — | — |  |
| 24 | 1.8 | 1.75, 1.85 | <0.001 | 24 | 0.84 | 0.75, 0.95 | 0.003 |
| 28 | 2.78 | 2.71, 2.84 | <0.001 | 28 | 0.38 | 0.34, 0.43 | <0.001 |
| 31 | 2.02 | 1.97, 2.07 | <0.001 | 31 | 0.52 | 0.46, 0.58 | <0.001 |
| 34 | 2.36 | 2.31, 2.42 | <0.001 | 34 | 0.83 | 0.74, 0.93 | <0.001 |
| Treatments |  |  |  | Treatments |  |  |  |
| Healthy | — | — |  | Healthy | — | — |  |
| Control | 1.07 | 1.06, 1.09 | <0.001 | Control | 0.76 | 0.70, 0.83 | <0.001 |
| Malaria | 1.28 | 1.26, 1.30 | <0.001 | Malaria | 0.46 | 0.42, 0.51 | <0.001 |
| Duration |  |  |  | Duration |  |  |  |
| 0000-0300 h | — | — |  | 0000-0300 h | — | — |  |
| 0400-0700 h | 0.89 | 0.87, 0.91 | <0.001 | 0400-0700 h | 2.23 | 2.02, 2.47 | <0.001 |
| 0800-1100 h | 0.59 | 0.57, 0.62 | <0.001 | 0800-1100 h | 12.4 | 11.0, 14.0 | <0.001 |
| 1200-1500 h | 0.42 | 0.39, 0.46 | <0.001 | 1200-1500 h | 35.1 | 29.9, 41.3 | <0.001 |
| 1600-1900 h | 1.42 | 1.38, 1.47 | <0.001 | 1600-1900 h | 12.1 | 10.7, 13.6 | <0.001 |
| 2000-2300 h | 3.27 | 3.22, 3.33 | <0.001 | 2000-2300 h | 0.38 | 0.34, 0.43 | <0.001 |
