# Supplementary material for: Regulation of diel locomotor activity and retinal responses of Anopheles stephensi by ingested histamine and serotonin is temperature- and infection-dependent
Source: PLoS Pathog. 2025 Apr 28;21(4):e1013139. doi: 10.1371/journal.ppat.1013139 (PMC12058162; doi:10.1371/journal.ppat.1013139)
Supplement: S11 Table — Treatments included malaria-associated biogenic amine treatment (10nM H + 0.15 μM 5-HT), healthy-associated treatment (1nM H + 1.5 μM 5-HT), or water (control). (DOCX) [file ppat.1013139.s023.docx]

**S11 Table.** Pairwise comparisons (Tukey HSD) of diel locomotor activity levels (i) between infected and uninfected mosquitoes by treatment and time, and (ii) comparison between time periods to determine activity patterns among treatment groups of infected and uninfected mosquitoes at days 4, 10, and 14 post-bloodmeal. Treatments included malaria-associated biogenic amine treatment (10nM H + 0.15μM 5-HT), healthy-associated treatment (1nM H + 1.5μM 5-HT), or water (control).

| Comparison of diel locomotor activity levels between treatment groups of infected and uninfected mosquitoes | | | |
| --- | --- | --- | --- |
| **Infected** | | | |
| **Treatments** | **t Ratio** | **Prob>\|t\|** | **Higher activity** |
| Healthy vs Malaria | -5.47 | <.0001* | Malaria |
| Heathy vs Control | 2.17 | 0.0758 | Healthy |
| Malaria vs Control | 7.78 | <.0001* | Malaria |
| **Uninfected** | | | |
| Healthy vs Malaria | -4.15 | 0.0001* | Malaria |
| Heathy vs Control | 2.21 | 0.0689 | Healthy |
| Malaria vs Control | 6.4 | <.0001* | Malaria |
| Comparison of diel locomotor activity between time periods among treatment groups of infected and uninfected mosquitoes at days 4, 10, and 14 post-bloodmeal | | | |
| **Infected** | | | |
| **Day 4 Healthy** | | | |
| **Period (24h)** | **t Ratio** | **Prob>\|t\|** | **Higher activity** |
| 0000-0300 vs 0400-0700 | 2.42 | 0.1098 | Similar |
| 0000-0300 vs 0800-1500 | 22.88 | <.0001* | 0000-0300 |
| 0000-0300 vs 1600-1900 | -10.57 | <.0001* | 1600-1900 |
| 0000-0300 vs 2000-2300 | -10.13 | <.0001* | 2000-2300 |
| 0400-0700 vs 0800-1500 | 20.33 | <.0001* | 0400-0700 |
| 0400-0700 vs 1600-1900 | -12.04 | <.0001* | 1600-1900 |
| 0400-0700 vs 2000-2300 | -11.69 | <.0001* | 2000-2300 |
| 0800-1500 vs 1600-1900 | -28.11 | <.0001* | 1600-1900 |
| 0800-1500 vs 2000-2300 | -30.16 | <.0001* | 2000-2300 |
| 1600-1900 vs 2000-2300 | -3.48 | 0.0047* | 2000-2300 |
| **Malaria** | | | |
| 0000-0300 vs 0400-0700 | 4.33 | 0.0002* | 0000-0300 |
| 0000-0300 vs 0800-1500 | 22.08 | <.0001* | 0000-0300 |
| 0000-0300 vs 1600-1900 | -3.2 | 0.0123* | 1600-1900 |
| 0000-0300 vs 2000-2300 | -7.62 | <.0001* | 2000-2300 |
| 0400-0700 vs 0800-1500 | 17.9 | <.0001* | 0400-0700 |
| 0400-0700 vs 1600-1900 | -6.82 | <.0001* | 1600-1900 |
| 0400-0700 vs 2000-2300 | -11.78 | <.0001* | 2000-2300 |
| 0800-1500 vs 1600-1900 | -23.69 | <.0001* | 1600-1900 |
| 0800-1500 vs 2000-2300 | -29.1 | <.0001* | 2000-2300 |
| 1600-1900 vs 2000-2300 | -3.01 | 0.022* | 2000-2300 |
| **Control** | | | |
| 0000-0300 vs 0400-0700 | 4.62 | <.0001* | 0000-0300 |
| 0000-0300 vs 0800-1500 | 24.68 | <.0001* | 0000-0300 |
| 0000-0300 vs 1600-1900 | -5.37 | <.0001* | 1600-1900 |
| 0000-0300 vs 2000-2300 | -6.12 | <.0001* | 2000-2300 |
| 0400-0700 vs 0800-1500 | 20.52 | <.0001* | 0400-0700 |
| 0400-0700 vs 1600-1900 | -9.61 | <.0001* | 1600-1900 |
| 0400-0700 vs 2000-2300 | -10.43 | <.0001* | 2000-2300 |
| 0800-1500 vs 1600-1900 | -28.13 | <.0001* | 1600-1900 |
| 0800-1500 vs 2000-2300 | -29.48 | <.0001* | 2000-2300 |
| 1600-1900 vs 2000-2300 | -0.1 | 1 | Similar |
| **Day 10 Healthy** | | | |
| 0000-0300 vs 0400-0700 | 9.55 | <.0001* | 0000-0300 |
| 0000-0300 vs 0800-1500 | 30.39 | <.0001* | 0000-0300 |
| 0000-0300 vs 1600-1900 | -7.72 | <.0001* | 1600-1900 |
| 0000-0300 vs 2000-2300 | -12.63 | <.0001* | 2000-2300 |
| 0400-0700 vs 0800-1500 | 21.24 | <.0001* | 0400-0700 |
| 0400-0700 vs 1600-1900 | -16.3 | <.0001* | 1600-1900 |
| 0400-0700 vs 2000-2300 | -21.44 | <.0001* | 2000-2300 |
| 0800-1500 vs 1600-1900 | -35.57 | <.0001* | 1600-1900 |
| 0800-1500 vs 2000-2300 | -41.05 | <.0001* | 2000-2300 |
| 1600-1900 vs 2000-2300 | -3.49 | 0.0046* | 2000-2300 |
| **Malaria** | | | |
| 0000-0300 vs 0400-0700 | 7.41 | <.0001* | 0000-0300 |
| 0000-0300 vs 0800-1500 | 30.71 | <.0001* | 0000-0300 |
| 0000-0300 vs 1600-1900 | -6.63 | <.0001* | 1600-1900 |
| 0000-0300 vs 2000-2300 | -14.16 | <.0001* | 2000-2300 |
| 0400-0700 vs 0800-1500 | 22.92 | <.0001* | 0400-0700 |
| 0400-0700 vs 1600-1900 | -13.6 | <.0001* | 1600-1900 |
| 0400-0700 vs 2000-2300 | -21.04 | <.0001* | 2000-2300 |
| 0800-1500 vs 1600-1900 | -36.16 | <.0001* | 1600-1900 |
| 0800-1500 vs 2000-2300 | -44.24 | <.0001* | 2000-2300 |
| 1600-1900 vs 2000-2300 | -6.57 | <.0001* | 2000-2300 |
| **Control** | | | |
| 0000-0300 vs 0400-0700 | 8.89 | <.0001* | 0000-0300 |
| 0000-0300 vs 0800-1500 | 33.24 | <.0001* | 0000-0300 |
| 0000-0300 vs 1600-1900 | -8.22 | <.0001* | 1600-1900 |
| 0000-0300 vs 2000-2300 | -11.46 | <.0001* | 2000-2300 |
| 0400-0700 vs 0800-1500 | 25.09 | <.0001* | 0400-0700 |
| 0400-0700 vs 1600-1900 | -16.3 | <.0001* | 1600-1900 |
| 0400-0700 vs 2000-2300 | -19.95 | <.0001* | 2000-2300 |
| 0800-1500 vs 1600-1900 | -38.8 | <.0001* | 1600-1900 |
| 0800-1500 vs 2000-2300 | -42.8 | <.0001* | 2000-2300 |
| 1600-1900 vs 2000-2300 | -2.25 | 0.1608 | Similar |
| **Day 14 Healthy** | | | |
| 0000-0300 vs 0400-0700 | 3.28 | 0.0093* | 0000-0300 |
| 0000-0300 vs 0800-1500 | 20.41 | <.0001* | 0000-0300 |
| 0000-0300 vs 1600-1900 | -6.78 | <.0001* | 1600-1900 |
| 0000-0300 vs 2000-2300 | -9.3 | <.0001* | 2000-2300 |
| 0400-0700 vs 0800-1500 | 16.98 | <.0001* | 0400-0700 |
| 0400-0700 vs 1600-1900 | -9.25 | <.0001* | 1600-1900 |
| 0400-0700 vs 2000-2300 | -11.95 | <.0001* | 2000-2300 |
| 0800-1500 vs 1600-1900 | -23.67 | <.0001* | 1600-1900 |
| 0800-1500 vs 2000-2300 | -28.21 | <.0001* | 2000-2300 |
| 1600-1900 vs 2000-2300 | -0.05 | 1 | Similar |
| **Malaria** | | | |
| 0000-0300 vs 0400-0700 | 5.96 | <.0001* | 0000-0300 |
| 0000-0300 vs 0800-1500 | 21.45 | <.0001* | 0000-0300 |
| 0000-0300 vs 1600-1900 | -9.92 | <.0001* | 1600-1900 |
| 0000-0300 vs 2000-2300 | -12.36 | <.0001* | 2000-2300 |
| 0400-0700 vs 0800-1500 | 14.54 | <.0001* | 0400-0700 |
| 0400-0700 vs 1600-1900 | -14.25 | <.0001* | 1600-1900 |
| 0400-0700 vs 2000-2300 | -16.2 | <.0001* | 2000-2300 |
| 0800-1500 vs 1600-1900 | -28.12 | <.0001* | 1600-1900 |
| 0800-1500 vs 2000-2300 | -31.55 | <.0001* | 2000-2300 |
| 1600-1900 vs 2000-2300 | -0.12 | 0.9999 | Similar |
| **Control** | | | |
| 0000-0300 vs 0400-0700 | 2.68 | 0.0569 | Similar |
| 0000-0300 vs 0800-1500 | 9.06 | <.0001* | 0000-0300 |
| 0000-0300 vs 1600-1900 | -8.59 | <.0001* | 1600-1900 |
| 0000-0300 vs 2000-2300 | -9.18 | <.0001* | 2000-2300 |
| 0400-0700 vs 0800-1500 | 6.39 | <.0001* | 0400-0700 |
| 0400-0700 vs 1600-1900 | -10.41 | <.0001* | 1600-1900 |
| 0400-0700 vs 2000-2300 | -11.04 | <.0001* | 2000-2300 |
| 0800-1500 vs 1600-1900 | -15.89 | <.0001* | 1600-1900 |
| 0800-1500 vs 2000-2300 | -16.57 | <.0001* | 2000-2300 |
| 1600-1900 vs 2000-2300 | 2.15 | 0.1981 | Similar |
| **Uninfected** | | | |
| **Day 4 Healthy** | | | |
| 0000-0300 vs 0400-0700 | 3.65 | 0.0025 | Similar |
| 0000-0300 vs 0800-1500 | 24.07 | <.0001* | 0000-0300 |
| 0000-0300 vs 1600-1900 | -11.23 | <.0001* | 1600-1900 |
| 0000-0300 vs 2000-2300 | -12.08 | <.0001* | 2000-2300 |
| 0400-0700 vs 0800-1500 | 20.28 | <.0001* | 0400-0700 |
| 0400-0700 vs 1600-1900 | -14.21 | <.0001* | 1600-1900 |
| 0400-0700 vs 2000-2300 | -15.16 | <.0001* | 2000-2300 |
| 0800-1500 vs 1600-1900 | -32.59 | <.0001* | 1600-1900 |
| 0800-1500 vs 2000-2300 | -34.71 | <.0001* | 2000-2300 |
| 1600-1900 vs 2000-2300 | 0.73 | 0.9485 | Similar |
| **Malaria** | | | |
| 0000-0300 vs 0400-0700 | 4.55 | <.0001* | 0000-0300 |
| 0000-0300 vs 0800-1500 | 25.25 | <.0001* | 0000-0300 |
| 0000-0300 vs 1600-1900 | -5.64 | <.0001* | 1600-1900 |
| 0000-0300 vs 2000-2300 | -10.44 | <.0001* | 2000-2300 |
| 0400-0700 vs 0800-1500 | 20.73 | <.0001* | 0400-0700 |
| 0400-0700 vs 1600-1900 | -9.56 | <.0001* | 1600-1900 |
| 0400-0700 vs 2000-2300 | -14.82 | <.0001* | 2000-2300 |
| 0800-1500 vs 1600-1900 | -28.76 | <.0001* | 1600-1900 |
| 0800-1500 vs 2000-2300 | -35.06 | <.0001* | 2000-2300 |
| 1600-1900 vs 2000-2300 | -3.27 | 0.0097* | 2000-2300 |
| **Control** | | | |
| 0000-0300 vs 0400-0700 | 7 | <.0001* | 0000-0300 |
| 0000-0300 vs 0800-1500 | 23.09 | <.0001* | 0000-0300 |
| 0000-0300 vs 1600-1900 | -7.13 | <.0001* | 1600-1900 |
| 0000-0300 vs 2000-2300 | -9.39 | <.0001* | 2000-2300 |
| 0400-0700 vs 0800-1500 | 15.9 | <.0001* | 0400-0700 |
| 0400-0700 vs 1600-1900 | -13.34 | <.0001* | 1600-1900 |
| 0400-0700 vs 2000-2300 | -15.59 | <.0001* | 2000-2300 |
| 0800-1500 vs 1600-1900 | -28.47 | <.0001* | 1600-1900 |
| 0800-1500 vs 2000-2300 | -31.29 | <.0001* | 2000-2300 |
| 1600-1900 vs 2000-2300 | -0.92 | 0.8882 | Similar |
| **Day 10 Healthy** | | | |
| 0000-0300 vs 0400-0700 | 8.9 | <.0001* | 0000-0300 |
| 0000-0300 vs 0800-1500 | 32.02 | <.0001* | 0000-0300 |
| 0000-0300 vs 1600-1900 | -6.03 | <.0001* | 1600-1900 |
| 0000-0300 vs 2000-2300 | -12.89 | <.0001* | 2000-2300 |
| 0400-0700 vs 0800-1500 | 23.43 | <.0001* | 0400-0700 |
| 0400-0700 vs 1600-1900 | -13.89 | <.0001* | 1600-1900 |
| 0400-0700 vs 2000-2300 | -20.87 | <.0001* | 2000-2300 |
| 0800-1500 vs 1600-1900 | -35.2 | <.0001* | 1600-1900 |
| 0800-1500 vs 2000-2300 | -42.53 | <.0001* | 2000-2300 |
| 1600-1900 vs 2000-2300 | -5.33 | <.0001* | 2000-2300 |
| **Malaria** | | | |
| 0000-0300 vs 0400-0700 | 9.52 | <.0001* | 0000-0300 |
| 0000-0300 vs 0800-1500 | 27.16 | <.0001* | 0000-0300 |
| 0000-0300 vs 1600-1900 | -2.56 | 0.0789 | 1600-1900 |
| 0000-0300 vs 2000-2300 | -11.63 | <.0001* | 2000-2300 |
| 0400-0700 vs 0800-1500 | 17.63 | <.0001* | 0400-0700 |
| 0400-0700 vs 1600-1900 | -11.15 | <.0001* | 1600-1900 |
| 0400-0700 vs 2000-2300 | -20.81 | <.0001* | 2000-2300 |
| 0800-1500 vs 1600-1900 | -28.07 | <.0001* | 1600-1900 |
| 0800-1500 vs 2000-2300 | -38.12 | <.0001* | 2000-2300 |
| 1600-1900 vs 2000-2300 | -7.84 | <.0001* | 2000-2300 |
| **Control** | | | |
| 0000-0300 vs 0400-0700 | 8.39 | <.0001* | 0000-0300 |
| 0000-0300 vs 0800-1500 | 23.25 | <.0001* | 0000-0300 |
| 0000-0300 vs 1600-1900 | -7.1 | <.0001* | 1600-1900 |
| 0000-0300 vs 2000-2300 | -12.72 | <.0001* | 2000-2300 |
| 0400-0700 vs 0800-1500 | 14.36 | <.0001* | 0400-0700 |
| 0400-0700 vs 1600-1900 | -14.28 | <.0001* | 1600-1900 |
| 0400-0700 vs 2000-2300 | -20.19 | <.0001* | 2000-2300 |
| 0800-1500 vs 1600-1900 | -28.06 | <.0001* | 1600-1900 |
| 0800-1500 vs 2000-2300 | -35.03 | <.0001* | 2000-2300 |
| 1600-1900 vs 2000-2300 | -3.73 | 0.0019* | 2000-2300 |
| **Day 14 Healthy** | | | |
| 0000-0300 vs 0400-0700 | 4.7 | <.0001* | 0000-0300 |
| 0000-0300 vs 0800-1500 | 25.07 | <.0001* | 0000-0300 |
| 0000-0300 vs 1600-1900 | -8.22 | <.0001* | 1600-1900 |
| 0000-0300 vs 2000-2300 | -13.19 | <.0001* | 2000-2300 |
| 0400-0700 vs 0800-1500 | 20.24 | <.0001* | 0400-0700 |
| 0400-0700 vs 1600-1900 | -12.38 | <.0001* | 1600-1900 |
| 0400-0700 vs 2000-2300 | -17.46 | <.0001* | 2000-2300 |
| 0800-1500 vs 1600-1900 | -31.4 | <.0001* | 1600-1900 |
| 0800-1500 vs 2000-2300 | -37.31 | <.0001* | 2000-2300 |
| 1600-1900 vs 2000-2300 | -3.41 | 0.006* | 2000-2300 |
| **Malaria** | | | |
| 0000-0300 vs 0400-0700 | 8.9 | <.0001* | 0000-0300 |
| 0000-0300 vs 0800-1500 | 21.72 | <.0001* | 0000-0300 |
| 0000-0300 vs 1600-1900 | -7.97 | <.0001* | 1600-1900 |
| 0000-0300 vs 2000-2300 | -13.17 | <.0001* | 2000-2300 |
| 0400-0700 vs 0800-1500 | 12.7 | <.0001* | 0400-0700 |
| 0400-0700 vs 1600-1900 | -15.25 | <.0001* | 1600-1900 |
| 0400-0700 vs 2000-2300 | -20.62 | <.0001* | 2000-2300 |
| 0800-1500 vs 1600-1900 | -26.92 | <.0001* | 1600-1900 |
| 0800-1500 vs 2000-2300 | -32.82 | <.0001* | 2000-2300 |
| 1600-1900 vs 2000-2300 | -2.92 | 0.0291* | 2000-2300 |
| **Control** | | | |
| 0000-0300 vs 0400-0700 | 6.28 | <.0001* | 0000-0300 |
| 0000-0300 vs 0800-1500 | 31.97 | <.0001* | 0000-0300 |
| 0000-0300 vs 1600-1900 | -7.43 | <.0001* | 1600-1900 |
| 0000-0300 vs 2000-2300 | -11.61 | <.0001* | 2000-2300 |
| 0400-0700 vs 0800-1500 | 26.32 | <.0001* | 0400-0700 |
| 0400-0700 vs 1600-1900 | -13.11 | <.0001* | 1600-1900 |
| 0400-0700 vs 2000-2300 | -17.33 | <.0001* | 2000-2300 |
| 0800-1500 vs 1600-1900 | -36.8 | <.0001* | 1600-1900 |
| 0800-1500 vs 2000-2300 | -40.98 | <.0001* | 2000-2300 |
| 1600-1900 vs 2000-2300 | -3.2 | 0.0123* | 2000-2300 |

P values ≤ 0.05 were considered significant and denoted with asterisk (*)
