# Supplementary material for: Regulation of diel locomotor activity and retinal responses of Anopheles stephensi by ingested histamine and serotonin is temperature- and infection-dependent
Source: PLoS Pathog. 2025 Apr 28;21(4):e1013139. doi: 10.1371/journal.ppat.1013139 (PMC12058162; doi:10.1371/journal.ppat.1013139)
Supplement: S12 Table — Treatments included malaria-associated biogenic amine treatment (10nM H + 0.15 μM 5-HT), healthy-associated treatment (1nM H + 1.5 μM 5-HT), or water (control). (DOCX) [file ppat.1013139.s024.docx]

**S12 Table.** Pairwise comparisons (Tukey HSD) of diel locomotor activity levels between treatments among infected and uninfected mosquitoes at days 4, 10, and 14 post-bloodmeal. Treatments included malaria-associated biogenic amine treatment (10nM H + 0.15μM 5-HT), healthy-associated treatment (1nM H + 1.5μM 5-HT), or water (control).

| **Infected** | | | | | | | | | |
| --- | --- | --- | --- | --- | --- | --- | --- | --- | --- |
|  | **Day 4** | | |  | **Day 10** |  |  | **Day 14** |  |
| **Treatments** | **t Ratio** | **Prob>\|t\|** | **Higher activity** | **t Ratio** | **Prob>\|t\|** | **Higher activity** | **t Ratio** | **Prob>\|t\|** | **Higher activity** |
| 0000-0300 |  |  |  |  |  |  |  |  |  |
| Healthy vs Malaria | -7.46 | <.0001* | Malaria | -2.55 | 0.0297* | Malaria | -0.24 | 0.9696 | Similar |
| Healthy vs Control | -1.48 | 0.3029 | Similar | -1.3 | 0.395 | Similar | 1.96 | 0.1235 | Similar |
| Malaria vs Control | 5.95 | <.0001* | Malaria | 1.24 | 0.4304 | Similar | 2.21 | 0.0699 | Similar |
| 0400-0700 |  |  |  |  |  |  |  |  |  |
| Healthy vs Malaria | -4.06 | 0.0002* | Malaria | -4.71 | <.0001 | Malaria | 2.09 | 0.0916 | Similar |
| Healthy vs Control | 0.91 | 0.6312 | Similar | -2.8 | 0.0144* | Control | 1.24 | 0.4309 | Similar |
| Malaria vs Control | 5.14 | <.0001* | Malaria | 1.96 | 0.123 | Similar | -0.87 | 0.6594 | Similar |
| 0800-1100 |  |  |  |  |  |  |  |  |  |
| Healthy vs Malaria | -7.6 | <.0001* | Malaria | -3.04 | 0.0068* | Malaria | 0.27 | 0.96 | Similar |
| Healthy vs Control | -2.23 | 0.0676 | Similar | -1.92 | 0.1329 | Similar | -2.41 | 0.0433* | Control |
| Malaria vs Control | 5.73 | <.0001* | Malaria | 1.13 | 0.494 | Similar | -2.44 | 0.0393* | Control |
| 1200-1500 |  |  |  |  |  |  |  |  |  |
| Healthy vs Malaria | 2.34 | 0.0515 | Similar | -1 | 0.5802 | Similar | -1.8 | 0.1709 | Similar |
| Healthy vs Control | 2.12 | 0.0866 | Similar | 2.35 | 0.0494* | Healthy | -3.02 | 0.0074* | Control |
| Malaria vs Control | 0.07 | 0.9972 | Similar | 3.28 | 0.0032* | Malaria | -1.51 | 0.2845 | Similar |
| 1600-1900 |  |  |  |  |  |  |  |  |  |
| Healthy vs Malaria | 2.31 | 0.0549 | Similar | -0.29 | 0.9543 | Similar | -1.36 | 0.3625 | Similar |
| Healthy vs Control | 3.86 | 0.0004* | Healthy | 0.02 | 0.9998 | Similar | -1.11 | 0.5067 | Similar |
| Malaria vs Control | 1.8 | 0.1717 | Similar | 0.32 | 0.9464 | Similar | 0.11 | 0.9936 | Similar |
| 2000-2300 |  |  |  |  |  |  |  |  |  |
| Healthy vs Malaria | -3.78 | 0.0005* | Malaria | -4.13 | 0.0001* | Malaria | -3.75 | 0.0006* | Malaria |
| Healthy vs Control | 2.55 | 0.0299* | Healthy | 2.08 | 0.0955 | Similar | 1.24 | 0.4313 | Similar |
| Malaria vs Control | 6.32 | <.0001* | Malaria | 6.2 | <.0001* | Malaria | 5 | <.0001* | Malaria |
| **Uninfected** | | | | | | | | | |
| 0000-0300 |  |  |  |  |  |  |  |  |  |
| Healthy vs Malaria | -6.7 | <.0001* | Malaria | -4.29 | <.0001* | Malaria | -3.7 | 0.0007* | Malaria |
| Healthy vs Control | -1.85 | 0.1558 | Similar | 2.44 | 0.0395* | Healthy | -2.17 | 0.0777 | Similar |
| Malaria vs Control | 4.91 | <.0001* | Malaria | 6.7 | <.0001* | Malaria | 1.57 | 0.2581 | Similar |
| 0400-0700 |  |  |  |  |  |  |  |  |  |
| Healthy vs Malaria | -4.68 | <.0001* | Malaria | -3.24 | 0.0036* | Malaria | 2.32 | 0.0543 | Similar |
| Healthy vs Control | 2.91 | 0.0105* | Healthy | 1.94 | 0.1289 | Similar | -0.83 | 0.6858 | Similar |
| Malaria vs Control | 7.37 | <.0001* | Malaria | 5.19 | <.0001* | Malaria | -3.1 | 0.0057* | Control |
| 0800-1100 |  |  |  |  |  |  |  |  |  |
| Healthy vs Malaria | -0.96 | 0.6021 | Similar | -3.86 | 0.0004* | Malaria | -2.82 | 0.0136* | Malaria |
| Healthy vs Control | -2.73 | 0.0176* | Control | -3.05 | 0.0066* | Malaria | 1.54 | 0.272 | Similar |
| Malaria vs Control | -2.21 | 0.0699 | Similar | 1.15 | 0.4861 | Similar | 3.84 | 0.0004* | Malaria |
| 1200-1500 |  |  |  |  |  |  |  |  |  |
| Healthy vs Malaria | -0.79 | 0.7067 | Similar | -4.85 | <.0001* | Malaria | -0.27 | 0.9616 | Similar |
| Healthy vs Control | -0.76 | 0.7305 | Similar | -2.9 | 0.0108* | Control | 2.35 | 0.0502 | Similar |
| Malaria vs Control | -0.03 | 0.9995 | Similar | 2.8 | 0.0146* | Malaria | 2.63 | 0.0235* | Malaria |
| 1600-1900 |  |  |  |  |  |  |  |  |  |
| Healthy vs Malaria | 0.37 | 0.9283 | Similar | 0.29 | 0.9539 | Similar | -0.51 | 0.8657 | Similar |
| Healthy vs Control | 0.78 | 0.7152 | Similar | 0.19 | 0.9793 | Similar | 0.42 | 0.9078 | Similar |
| Malaria vs Control | 0.42 | 0.9083 | Similar | -0.09 | 0.9956 | Similar | 0.92 | 0.6306 | Similar |
| 2000-2300 |  |  |  |  |  |  |  |  |  |
| Healthy vs Malaria | -4.19 | <.0001* | Malaria | -2.47 | 0.0369* | Malaria | -2.66 | 0.0216* | Malaria |
| Healthy vs Control | 0.26 | 0.9643 | Similar | 1.97 | 0.1203 | Similar | 2.54 | 0.0305* | Healthy |
| Malaria vs Control | 4.41 | <.0001* | Malaria | 4.44 | <.0001* | Malaria | 5.21 | <.0001* | Malaria |

P values ≤ 0.05 were considered significant and denoted with asterisk (*)
