# Supplementary material for: Regulation of diel locomotor activity and retinal responses of Anopheles stephensi by ingested histamine and serotonin is temperature- and infection-dependent
Source: PLoS Pathog. 2025 Apr 28;21(4):e1013139. doi: 10.1371/journal.ppat.1013139 (PMC12058162; doi:10.1371/journal.ppat.1013139)
Supplement: S13 Table — Treatments included malaria-associated biogenic amine treatment (10nM H + 0.15 μM 5-HT), healthy-associated treatment (1nM H + 1.5 μM 5-HT), or water (control). (DOCX) [file ppat.1013139.s025.docx]

**S13 Table.** Pairwise comparisons (Tukey HSD) of diel locomotor activity levels between uninfected and infected mosquitoes among treatments at days 4, 10, and 14 post-bloodmeal. Treatments included malaria-associated biogenic amine treatment (10nM H + 0.15μM 5-HT), healthy-associated treatment (1nM H + 1.5μM 5-HT), or water (control).

| **Day 4** | | | | | | | | | |
| --- | --- | --- | --- | --- | --- | --- | --- | --- | --- |
|  | **Control** | | | **Healthy** | | | **Malaria** | | |
| **Infection status** | **t Ratio** | **Prob>\|t\|** | **Higher activity** | **t Ratio** | **Prob>\|t\|** | **Higher activity** | **t Ratio** | **Prob>\|t\|** | **Higher activity** |
| 0000-0300 |  |  |  |  |  |  |  |  |  |
| Infected vs Uninfected | -2.28 | 0.0229* | Uninfected | -1.8 | 0.073 | Similar | -1.31 | 0.1902 | Similar |
| 0400-0700 |  |  |  |  |  |  |  |  |  |
| Infected vs Uninfected | 1.11 | 0.2675 | Similar | -0.44 | 0.6578 | Similar | -1.11 | 0.2676 | Similar |
| 0800-1100 |  |  |  |  |  |  |  |  |  |
| Infected vs Uninfected | -2.19 | 0.0288* | Uninfected | -1.24 | 0.2144 | Similar | 1.48 | 0.1406 | Similar |
| 1200-1500 |  |  |  |  |  |  |  |  |  |
| Infected vs Uninfected | -1.34 | 0.1803 | Similar | 1.13 | 0.2603 | Similar | -2.34 | 0.0198* | Uninfected |
| 1600-1900 |  |  |  |  |  |  |  |  |  |
| Infected vs Uninfected | -3.45 | 0.0006* | Uninfected | 0.03 | 0.9775 | Similar | -2.29 | 0.0227* | Uninfected |
| 2000-2300 |  |  |  |  |  |  |  |  |  |
| Infected vs Uninfected | -5.84 | <.0001* | Uninfected | -4.49 | <.0001* | Uninfected | -4.21 | <.0001* | Uninfected |
| **Day 10** | | | | | | | | | |
| 0000-0300 |  |  |  |  |  |  |  |  |  |
| Infected vs Uninfected | 2.16 | 0.0309* | Infected | -1.47 | 0.1424 | Similar | -3.3 | 0.001* | Uninfected |
| 0400-0700 |  |  |  |  |  |  |  |  |  |
| Infected vs Uninfected | 2.36 | 0.0187* | Infected | -2.36 | 0.0184* | Uninfected | -1.01 | 0.313 | Similar |
| 0800-1100 |  |  |  |  |  |  |  |  |  |
| Infected vs Uninfected | -2.72 | 0.0067* | Uninfected | -0.58 | 0.5604 | Similar | -4.51 | <.0001* | Uninfected |
| 1200-1500 |  |  |  |  |  |  |  |  |  |
| Infected vs Uninfected | -3.18 | 0.0016* | Uninfected | 1.43 | 0.1534 | Similar | -2.64 | 0.0085* | Uninfected |
| 1600-1900 |  |  |  |  |  |  |  |  |  |
| Infected vs Uninfected | 1.33 | 0.1854 | Similar | 1.27 | 0.2059 | Similar | 2.14 | 0.0326* | Infected |
| 2000-2300 |  |  |  |  |  |  |  |  |  |
| Infected vs Uninfected | -0.48 | 0.6315 | Similar | -0.02 | 0.9853 | Similar | 2.32 | 0.0205* | Infected |
| **Day 14** | | | | | | | | | |
| 0000-0300 |  |  |  |  |  |  |  |  |  |
| Infected vs Uninfected | -8.31 | <.0001* | Uninfected | -4.88 | <.0001* | Uninfected | -7.98 | <.0001* | Uninfected |
| 0400-0700 |  |  |  |  |  |  |  |  |  |
| Infected vs Uninfected | -5.13 | <.0001* | Uninfected | -3.08 | 0.0022* | Uninfected | -3.06 | 0.0023* | Uninfected |
| 0800-1100 |  |  |  |  |  |  |  |  |  |
| Infected vs Uninfected | 3.59 | 0.0004* | Infected | 0.31 | 0.7574 | Similar | -2.29 | 0.0224* | Uninfected |
| 1200-1500 |  |  |  |  |  |  |  |  |  |
| Infected vs Uninfected | 1.92 | 0.0553 | Similar | -3.42 | 0.0007* | Uninfected | -1.65 | 0.0996 | Similar |
| 1600-1900 |  |  |  |  |  |  |  |  |  |
| Infected vs Uninfected | -0.86 | 0.3901 | Similar | -2.8 | 0.0053* | Uninfected | -1.86 | 0.0639 | Similar |
| 2000-2300 |  |  |  |  |  |  |  |  |  |
| Infected vs Uninfected | -9.26 | <.0001* | Uninfected | -9.12 | <.0001* | Uninfected | -10.36 | <.0001* | Uninfected |

P values ≤ 0.05 were considered significant and denoted with asterisk (*)
